# Differential isoform-specific control of KCC2 function in developing and mature cortical neurons

**DOI:** 10.1038/s44319-026-00920-3

**Published:** 2026-09-05

**Authors:** Carla Pagan, Pauline Weinzettl, Marika A Markkanen, Marion Russeau, Matti S Airaksinen, Jean-Christophe Poncer

**Affiliations:** 1 Institut du Fer à Moulin, Inserm, Sorbonne Université, Paris, France; 2 Paris Brain Institute, AP-HP, CNRS, Inserm, Sorbonne Université, Paris, France; 3 Department of Anatomy, Faculty of Medicine, University of Helsinki, Helsinki, Finland

**Keywords:** Neuroscience

## Abstract

The K-Cl cotransporter KCC2 is essential for fast synaptic inhibition in the mature brain. It is encoded by a single gene and expressed as two isoforms: KCC2a and KCC2b, which differ in their N-terminal domains. While KCC2b is predominant, the function of the weakly expressed KCC2a isoform remains unclear. Here, we reveal that KCC2a is a potent, bidirectional regulator of KCC2b membrane stability and function in cortical neurons. In immature neurons, where WNK-SPAK kinase activity is high, KCC2a promotes SPAK-dependent phosphorylation of KCC2b at Thr1007, which likely contributes to hindering its membrane expression and function. Conversely, in mature neurons with low basal WNK-SPAK activity, KCC2a promotes KCC2b expression, clustering, and function. At this stage, although accounting for less than 5% of total KCC2 mRNA, KCC2a is enriched in dendrites and within KCC2 clusters, where it prevents clathrin-mediated KCC2b endocytosis, as well as polyubiquitination and proteasomal degradation. Thus, KCC2a acts as a developmental switch that first inhibits KCC2b during early development and then ensures its membrane stability to support effective synaptic inhibition in the adult brain.

## Synopsis

Despite its low expression in mature neurons, the minor KCC2a isoform acts as a developmental switch that first restrains and later stabilizes the predominant KCC2b isoform, controlling neuronal chloride transport in an age-dependent manner.

- Although it accounts for less than 5% of total KCC2 transcripts in mature neurons, KCC2a is enriched within dendritic KCC2 clusters, yet absent from dendritic spines, and exerts strong, non-stoichiometric control over the stability and function of the major KCC2b isoform.
- In mature neurons, KCC2a stabilizes KCC2b by preventing its clathrin-mediated endocytosis and proteasomal degradation, thereby sustaining KCC2 clustering and dendritic chloride extrusion.
- In immature neurons, where WNK-SPAK activity is high, KCC2a has the opposite effect: it couples KCC2b to the WNK-SPAK pathway and promotes its SPAK-dependent phosphorylation at Thr1007, limiting membrane expression.

## Abstract (web summary)

Despite its low expression in mature neurons, the minor KCC2a isoform acts as a developmental switch that first restrains and later stabilizes the predominant KCC2b isoform, controlling neuronal chloride transport in an age-dependent manner.

## Graphical abstract

**Figure:**
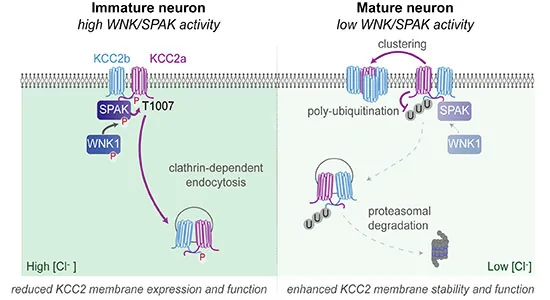

## Introduction

The KCC2 neuronal K-Cl cotransporter, which is encoded by the *SLC12A5* gene, is essential for maintaining the low intracellular chloride concentration required for fast hyperpolarizing synaptic responses mediated by inhibitory neurotransmitters, such as gamma-aminobutyric acid (GABA) and glycine, in the mature central nervous system. Upregulation of KCC2 expression is a hallmark of neuronal maturation (Rivera et al, 1999; Virtanen et al, 2021), mediating the developmental shift in GABAergic signaling from depolarizing to hyperpolarizing (Ben-Ari, 2002). Due to its pivotal role in chloride homeostasis and GABA signaling, KCC2 dysfunction has been associated with many neurological and psychiatric disorders (Kaila et al, 2014), including epilepsy (Donneger et al, 2026; Huberfeld et al, 2007), neuropathic pain (Coull et al, 2003), and Alzheimer’s disease (Keramidis et al, 2023).

The *SLC12A5* gene generates two full-length protein isoforms, KCC2a and KCC2b, via alternative promoters and splicing (Uvarov et al, 2007). These isoforms only differ in their unique N-terminal sequences. Whereas the fundamental role of KCC2b in chloride transport is well-established and primarily drives the developmental switch in GABA signaling in cortical neurons (Rivera et al, 1999; Uvarov et al, 2009), the regulatory profile and physiological function of KCC2a remain elusive.

Genetic studies suggest a critical and transient role of the KCC2a isoform, particularly in the early stages of postnatal development. Complete genetic ablation of *Slc12a5* in mice results in death at birth due to severe motor deficits (Hübner et al, 2001). However, mice lacking only KCC2b (while retaining KCC2a) survive for 2–3 weeks, suggesting that KCC2a expression alone is sufficient to support vital functions during the immediate postnatal period (Woo et al, 2002). Conversely, KCC2a-deficient mice exhibit transient respiratory anomalies at birth, including low breathing rates and apneas, resulting from abnormally strong inhibition from the pons (Dubois et al, 2018). This functional divergence is mirrored by the distinct developmental expression patterns of KCC2 isoforms. KCC2a accounts for a substantial fraction (50–65%) of total KCC2 RNA in the cortex, brainstem, and spinal cord on embryonic day 17 (Uvarov et al, 2007; Uvarov et al, 2009). However, its relative contribution decreases drastically in the adult forebrain, where KCC2b becomes the predominant isoform (> 90%) (Markkanen et al, 2014; Uvarov et al, 2007). Therefore, the differential regulation and developmental switch of KCC2 isoforms appear critical for circuit function, especially during the perinatal period.

Both KCC2a and KCC2b were shown to function as K-Cl cotransporters (Markkanen et al, 2017; Uvarov et al, 2007) and interact to form heterodimers in both heterologous cells and rat neurons (Uvarov et al, 2009). However, their distinct N-terminal sequences dictate different regulatory mechanisms, enabling the fine-tuning of K-Cl transport activity. In particular, the N-terminus of KCC2a, but not KCC2b, contains a conserved (RFxV/I) binding motif for the Ste20-related proline-alanine-rich kinase (SPAK) (Uvarov et al, 2009), an effector of the WNK (with-no-lysine) kinase pathway known to regulate cation-chloride cotransporters (Friedel et al, 2015; Heubl et al, 2017). Activation of the WNK-SPAK pathway results in the phosphorylation of KCC2b at Thr1007, leading to a subsequent decrease in membrane stability and chloride extrusion function (Friedel et al, 2015; Heubl et al, 2017). In heterologous cells, SPAK was found to co-immunoprecipitate specifically with recombinant KCC2a, but not with KCC2b (Markkanen et al, 2017). Functionally, the overexpression of SPAK significantly decreased the transport activity of KCC2a, yet it had no inhibitory effect on KCC2b in the absence of KCC2a (Markkanen et al, 2017). These results suggest that KCC2a may be essential for the inhibitory regulation of KCC2b by the WNK-SPAK signaling pathway.

Taken together, these data suggest that the N-terminal heterogeneity of KCC2 isoforms provides a mechanism for distinct regulation and developmental expression patterns. However, it remains unknown whether KCC2a maintains a key role in KCC2 function in the adult brain despite its low expression levels. If so, this isoform could be a prime target for new KCC2-modulating therapeutic compounds (Donneger et al, 2026). Here, we investigated the age-dependent function of KCC2a in hippocampal and neocortical neurons. In primary hippocampal cultures, we found that endogenous KCC2a is enriched within dendritic KCC2 clusters, likely promoting its interaction with KCC2b. Knockdown of KCC2a in mature neurons revealed that KCC2a stabilizes KCC2b protein levels and expression at the cell membrane by preventing endocytosis and proteasomal degradation. Using KCC2a knockout mice, we demonstrated that this stabilizing function only prevails in mature neurons with low WNK-SPAK activity. In contrast, in immature neurons with high WNK-SPAK activity, KCC2a acts oppositely by promoting the phosphorylation of KCC2b at Thr1007, thereby reducing its membrane expression. This effect is associated with increased WNK1-mediated SPAK phosphorylation at Ser383. This suggests that KCC2a may recruit a functional protein unit, which includes both KCC2b and SPAK, to regulate chloride transport in dendrites. Taken together, our results reveal a novel mechanism by which KCC2a acts as a context-dependent regulator of KCC2b. Despite its weak expression in mature neurons, KCC2a transitions from a negative regulator to a stabilizing partner of KCC2b in response to developmental shifts in WNK-SPAK signaling.

## Results

### KCC2 isoform expression and subcellular localization in mature cortical neurons

**Figure 1:**
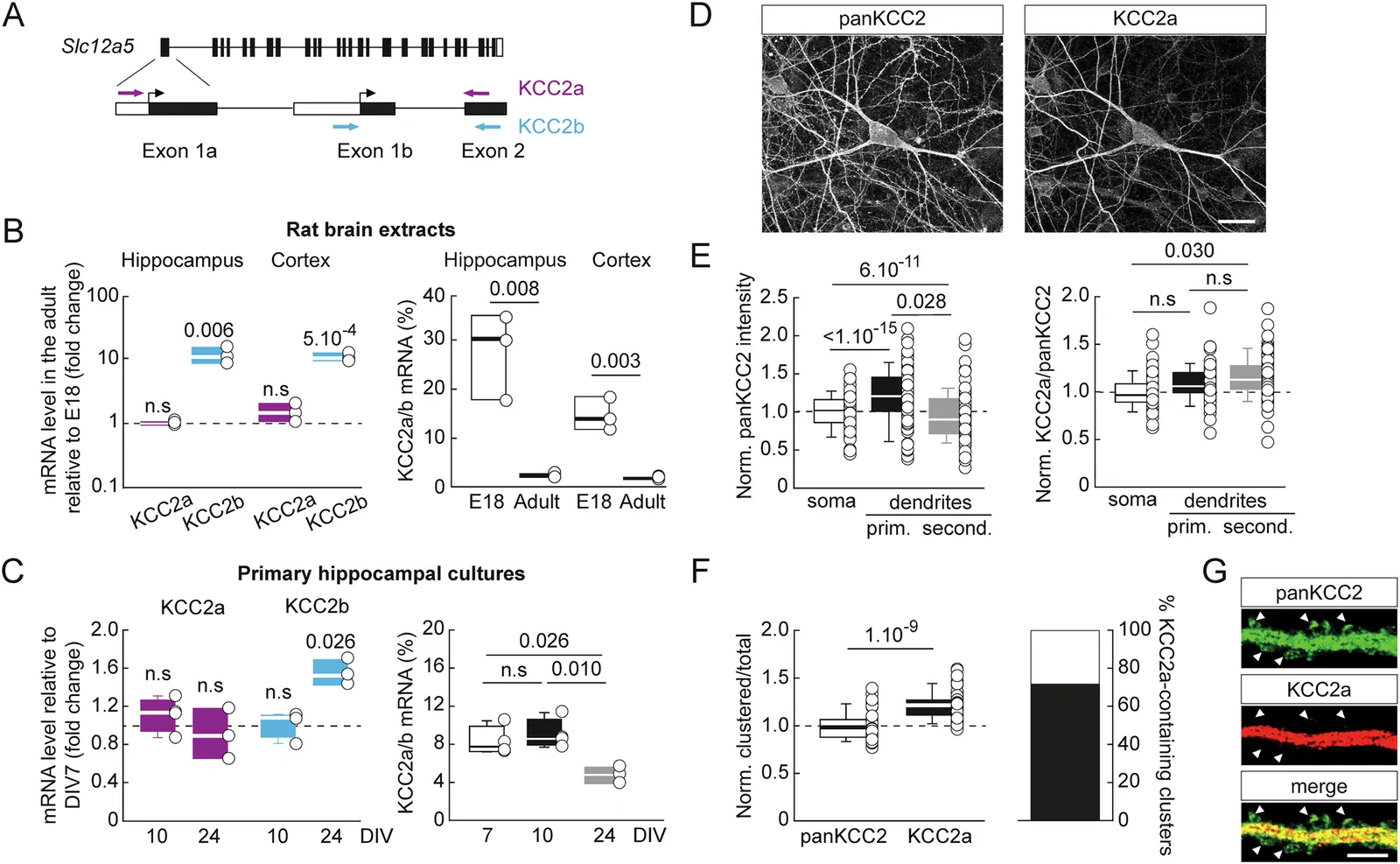
Expression pattern of KCC2 isoforms in rat hippocampal and cortical neurons. (**A**) Schematic representation of the complementary sequences of KCC2a- and KCC2b-specific primers. (**B**) Summary boxplots showing the quantitative real-time PCR analysis of KCC2a and KCC2b mRNA expression in the hippocampus and cortex of E18 and adult rats. Left: Isoform-specific mRNA expression relative to Ywhaz in adults, normalized to expression at E18. Right: KCC2a/KCC2b mRNA ratio in E18 embryos and in adults. *n* = 3 animals/age and 3 technical replicates. Two-tailed unpaired *t* test. Box plots show the median (centre line) and the 25th and 75th percentiles (box bounds); with *n* = 3, no whiskers are displayed. (**C**) Same as (**B**) in primary hippocampal cultures. Left: Isoform-specific mRNA expression relative to Ywhaz, normalized by expression at DIV7, one-way Kruskal–Wallis rank-sum test and Dunn’s post-hoc test. Right: KCC2a/KCC2b mRNA ratio as a function of time in vitro, one-way ANOVA and Tukey post-hoc test. *n* = 3–4 cultures and three technical replicates. Box plots show the median (centre line) and the 25th and 75th percentiles (box bounds); whiskers, displayed only for datasets with *n* = 4, extend to the minimum and maximum values. (**D**) Representative confocal maximum projection images of hippocampal pyramidal neurons immunostained with panKCC2 and KCC2a-specific antibodies. Scale: 25 µm. (**E**) Summary boxplots showing the intensity of panKCC2 immunofluorescence (left, one-way Kruskal–Wallis rank-sum test and Dunn’s post-hoc test) and the ratio of KCC2a to panKCC2 immunofluorescence (right, one-way ANOVA and Tukey post-hoc test), along primary (prim.) and secondary (second.) dendrites, normalized to the soma. *n* = 58 neurons from 2 cultures. (**F**) Left: summary boxplots showing the ratio of immunofluorescence intensity within clusters to total intensity in dendritic sections, for panKCC2 or KCC2a immunostainings, normalized to that of panKCC2. *n* = 46 neurons from 2 cultures, Mann–Whitney rank-sum test. Right: bar plot showing the percentage of panKCC2 clusters colocalizing (black) or not (white) with KCC2a-immunostained clusters. (**G**) Representative confocal images of a dendritic section immunostained with panKCC2 (green) and KCC2a (red) antibodies. Note the complete and specific lack of KCC2a immunostaining in dendritic spines (arrowheads). Scale: 5 µm. (**E**, **F**, left) Box plots show the median (centre line), the 25th and 75th percentiles (box bounds) and the 10th and 90th percentiles (whiskers); all individual data points, including minima and maxima, are displayed alongside the box (see “Methods”). *P* values are provided on the graphs.

First, we characterized and compared the expression of the two KCC2 isoforms, KCC2a and KCC2b, in maturing rat hippocampal neurons in vitro and in ex vivo samples from rat hippocampus and cortex. In both in vitro (from 7 to 24 DIV) and ex vivo (hippocampus and cortex from E18.5 and adult) samples, KCC2a mRNA levels remained stable throughout maturation (Fig. 1A–C). In contrast, KCC2b expression increased significantly by ≈12-fold in the hippocampus and cortex and by ≈1.5-fold in vitro between 7 and 24 DIV (Fig. 1B,C). This differential regulation resulted in a marked decrease in the KCC2a/KCC2b mRNA ratio both ex vivo and in vitro (Fig. 1B,C). These results confirm that primary hippocampal cultures recapitulate the dynamic developmental shift in KCC2 expression, with KCC2a accounting for less than 5% of total KCC2 mRNA expression in mature neurons.

**Figure EV1:**
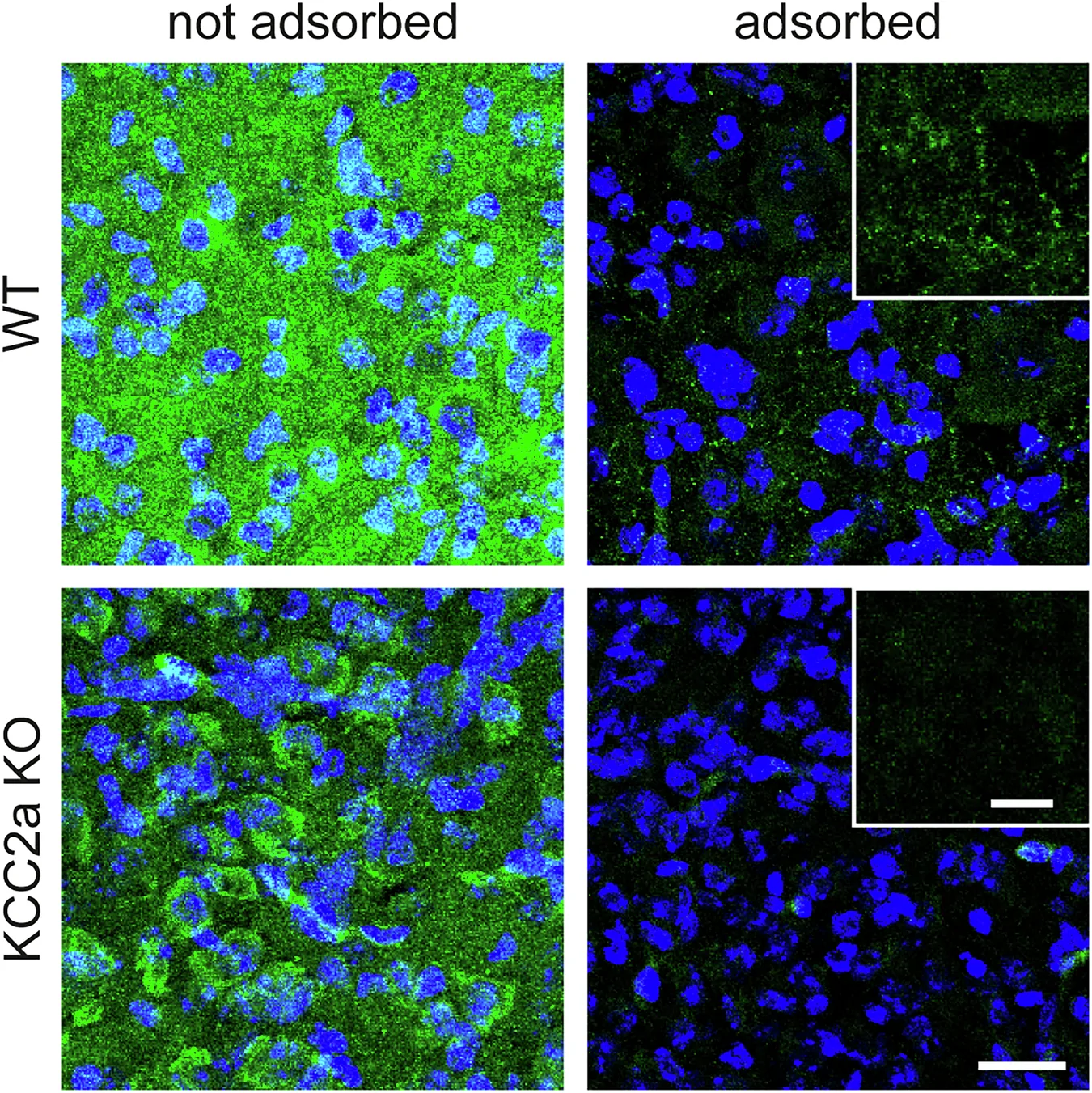
Specificity of the anti-KCC2a antibody (related to Fig. 1). Representative confocal maximum projection images of the brainstem from wild-type (WT) and KCC2a-knockout (KCC2a-KO) P9 pups. The sections were immunostained with our anti-KCC2a antibody (green), which was either pre-adsorbed on KCC2a-KO mice brainstems (right) or not pre-adsorbed (left), and then stained with DAPI (blue). Using pre-adsorbed anti-KCC2a antibody greatly reduces nonspecific background staining compared to non-pre-adsorbed antibody. The insets show KCC2a-specific immunostaining in WT but not KCC2a KO samples, at higher magnification. Scale: 20 µm and 10 µm (insets). Two animals per condition, 2 technical replicates.

Previous work in adult mouse brain suggested KCC2a may be preferentially enriched in distal dendrites compared to KCC2b (Markkanen et al, 2014). We therefore compared the subcellular localization of KCC2a and KCC2b in mature (24 DIV) hippocampal neurons using a panKCC2 antibody (recognizing both isoforms) and a KCC2a-specific antibody (Uvarov et al, 2009) (Fig. EV1). PanKCC2 immunostaining was 20% stronger in primary dendrites compared to the soma (*P* < 1.10^−15^; Fig. 1D,E). In addition, the KCC2a/panKCC2 fluorescence ratio was significantly increased in secondary dendrites compared to the soma (*P* = 0.030; Fig. 1E), demonstrating that KCC2a is relatively enriched in dendrites compared to KCC2b. As previously demonstrated (Al Awabdh et al, 2022; Chamma et al, 2013; Gauvain et al, 2011), KCC2 immunostaining exhibited heterogeneity, with clusters present in both the soma and dendrites of hippocampal neurons. Notably, a greater fraction of KCC2a immunofluorescence was found within clusters compared to panKCC2 (*P* = 1.10^−9^; Fig. 1F). Colocalization analysis showed that more than 70% of KCC2 clusters contain the KCC2a isoform (Fig. 1F). However, KCC2a-immunoreactivity was notably absent from dendritic spines (Fig. 1G), where KCC2 has been shown to form large clusters implicated in non-canonical, ion transport-independent functions (Gauvain et al, 2011). These results are consistent with an enrichment of KCC2a within subcellular domains critical for Cl^−^ extrusion and GABA signaling in mature hippocampal neurons (Raimondo et al, 2012).

### KCC2a controls KCC2b stability and function in mature hippocampal neurons

**Figure 2:**
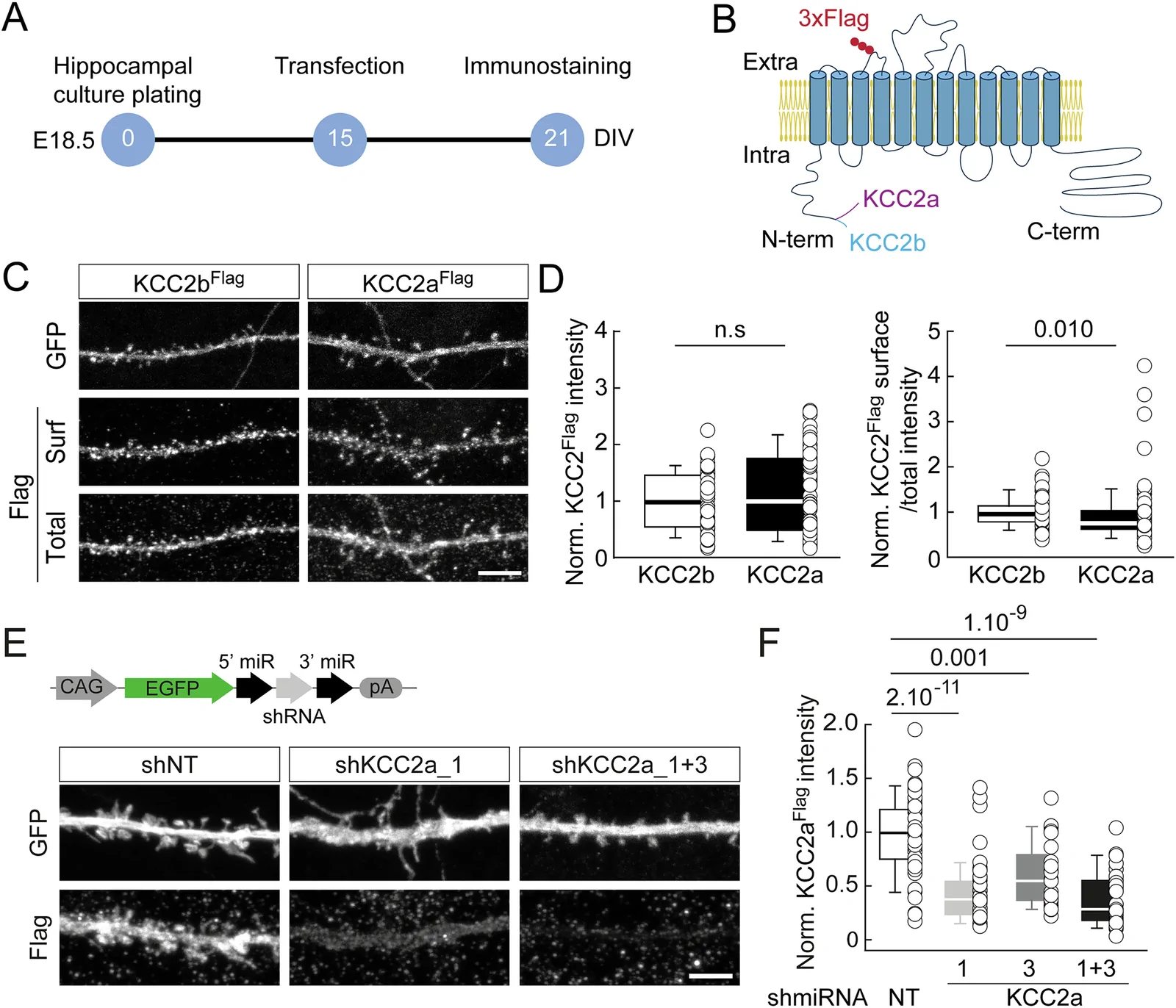
KCC2a-specific knockdown by RNA interference in mature hippocampal neurons. (**A**) Experiment timeline. (**B**) Schematic structure of the recombinant KCC2a-Flag and KCC2b-Flag proteins. (**C**) Representative confocal maximum projection images of dendritic sections of hippocampal neurons transfected with plasmids encoding for GFP and either KCC2b-Flag or KCC2a-Flag. Neurons were immunostained for plasmalemmal and total Flag (see “Methods”) as well as GFP. Scale: 5 µm. (**D**) Summary boxplots showing the immunofluorescence intensity of recombinant KCC2-Flag isoforms (left) and the ratio of membrane-inserted (surf.) to the total (total) KCC2-Flag immunofluorescence intensity normalized to that of KCC2b-Flag (right). KCC2b, *n* = 58; KCC2a, *n* = 65 cells from five independent cultures. Mann–Whitney rank-sum tests. (**E**) Top: Design of the plasmids encoding the shRNAs targeting KCC2a. Bottom: Representative confocal maximum projection images of dendritic sections of neurons transfected with plasmids encoding either for a control scrambled shRNA (shNT) or KCC2a-specific shRNAs (shKCC2a_1 and shKCC2a_3), as well as a plasmid encoding recombinant KCC2a-Flag. Neurons were immunostained for Flag and GFP. Scale: 5 µm. (**F**) Summary boxplot showing the immunofluorescence intensity of recombinant KCC2a-Flag, normalized to shNT. shNT, *n* = 53; shKCC2a_1, *n* = 50; shKCC2a_3, *n* = 25; shKCC2a1 + 3, *n* = 27 cells; from 4 (shNT and shKCC2a_1) or 2 (shKCC2a_3 and shKCC2a_1 + 3) independent cultures. One-way Kruskal–Wallis rank-sum test and Dunn’s post-hoc test. Box plots show the median (centre line), the 25th and 75th percentiles (box bounds) and the 10th and 90th percentiles (whiskers); all individual data points, including minima and maxima, are displayed alongside the box (see “Methods”). *P* values are provided on the graphs.

Despite its low expression levels, our data suggest that KCC2a may be optimally positioned to regulate chloride transport in mature hippocampal neurons. To evaluate the localization of each KCC2 isoform at the plasma membrane, we expressed recombinant KCC2a and KCC2b with a 3xFlag tag in their second extracellular loop, thereafter referred to as KCC2-Flag (Chamma et al, 2013; Fig. 2A,B). This enabled us to specifically detect KCC2 membrane pools using live immunostaining (Fig. 2A–C). The recombinant proteins showed no significant difference in total expression (*P* = 0.206; Fig. 2D). However, the relative membrane expression of recombinant KCC2a was slightly but significantly lower than that of KCC2b (*P* = 0.010; Fig. 2D), consistent with prior studies (Markkanen et al, 2014). These results suggest that, despite a small difference in membrane enrichment the overexpressed isoforms have similar inherent stability.

**Figure EV2:**
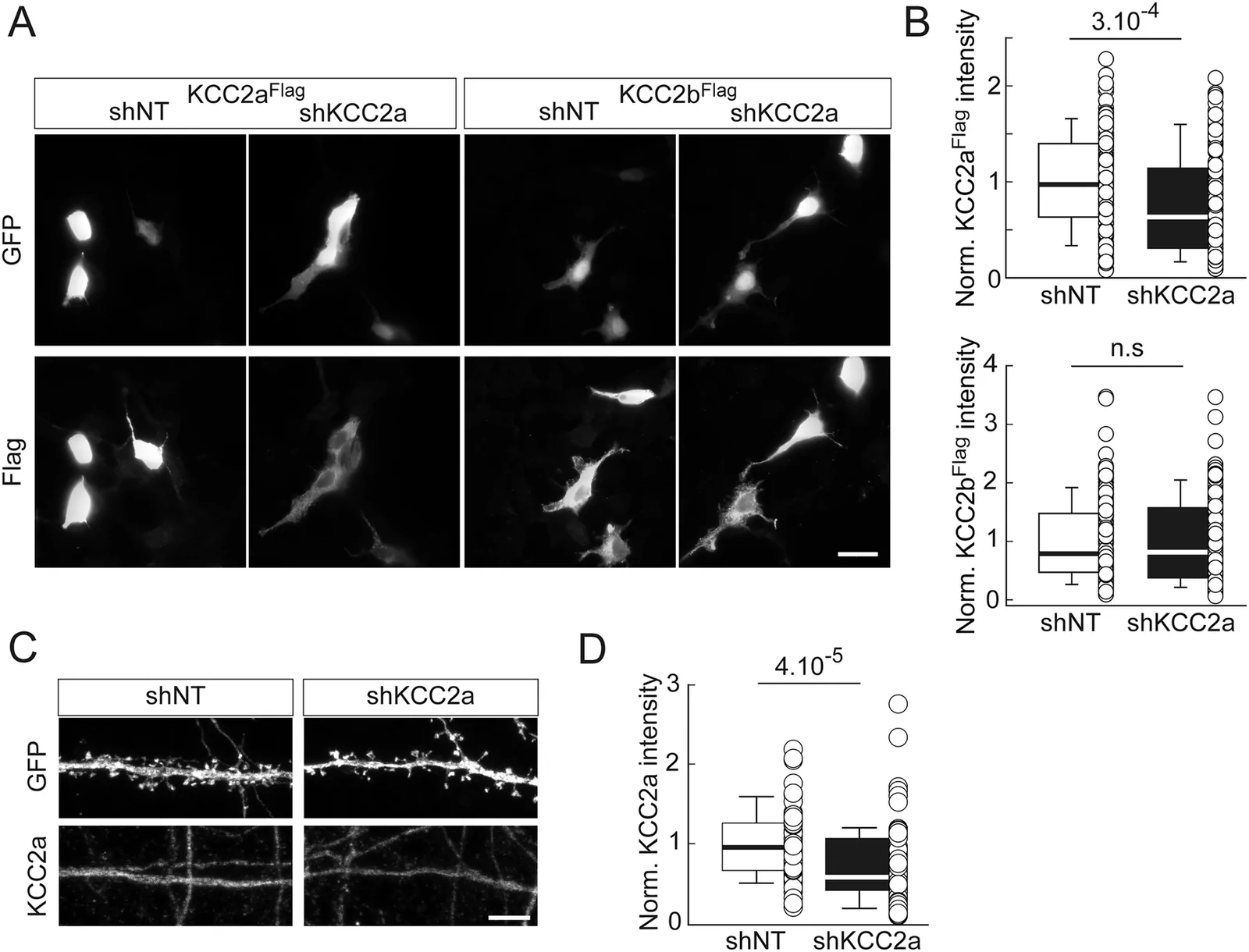
Specificity of KCC2a knockdown in heterologous cells and hippocampal neurons (related to Fig. 2). (**A**) Representative epifluorescence images of HEK-293 cells transfected with plasmids encoding either shNT or shKCC2a and either KCC2a-Flag or KCC2b-Flag. Cells were immunostained for Flag and GFP. Scale: 20 µm. (**B**) Summary boxplot showing the intensity of Flag immunostaining in HEK-293 cells expressing scrambled (shNT) or KCC2a-specific (shKCC2a) shRNAs and recombinant KCC2a-Flag (left) or KCC2b-Flag (right). shNT KCC2a-Flag, *n* = 131; shKCC2a KCC2a-Flag, *n* = 126; shNT KCC2b-Flag, *n* = 155; shKCC2a KCC2b-Flag, *n* = 153 cells, from three independent cultures. Mann–Whitney rank-sum test. All data are normalized to the corresponding shNT. (**C**) Representative confocal maximum projection images of dendritic sections of neurons transfected with plasmids encoding shNT or shKCC2a and immunostained for endogenous KCC2a. Scale: 5 µm. (**D**) Boxplot showing the intensity of endogenous KCC2a immunofluorescence in neurons transfected with shNT or shKCC2a_1 + 3. shNT, *n* = 73; shKCC2a, *n* = 74 cells, from five independent cultures (mean effect per culture: −38%, +17%, −13%, −41%, −76%). Mann–Whitney rank-sum test. Box plots show the median (centre line), the 25th and 75th percentiles (box bounds) and the 10th and 90th percentiles (whiskers); all individual data points, including minima and maxima, are displayed alongside the box (see “Methods”). *P* values are provided on the graphs.

To investigate the functional role of KCC2a in hippocampal neurons, we performed an isoform-specific knockdown using a combination of two shRNAs (shKCC2a_1 + 3). In HEK-293 cells expressing either recombinant KCC2a or KCC2b, these shRNAs specifically reduced KCC2a expression within 48 h, with no significant effect on KCC2b (Fig. EV2). In hippocampal neurons, chronic KCC2a knockdown (from DIV15) with the two shRNAs reduced recombinant KCC2a expression by over 60% (*P* = 1.10^−9^; Fig. 2E,F) and endogenous KCC2a levels by 25% (*P* = 4.10^−5^; Fig. EV2). We note, however, that this reduction was variable across the five independent hippocampal cultures examined (range: +17% to −76%), likely reflecting the limited sensitivity of the immunostaining assay for this low-abundance isoform.

**Figure 3:**
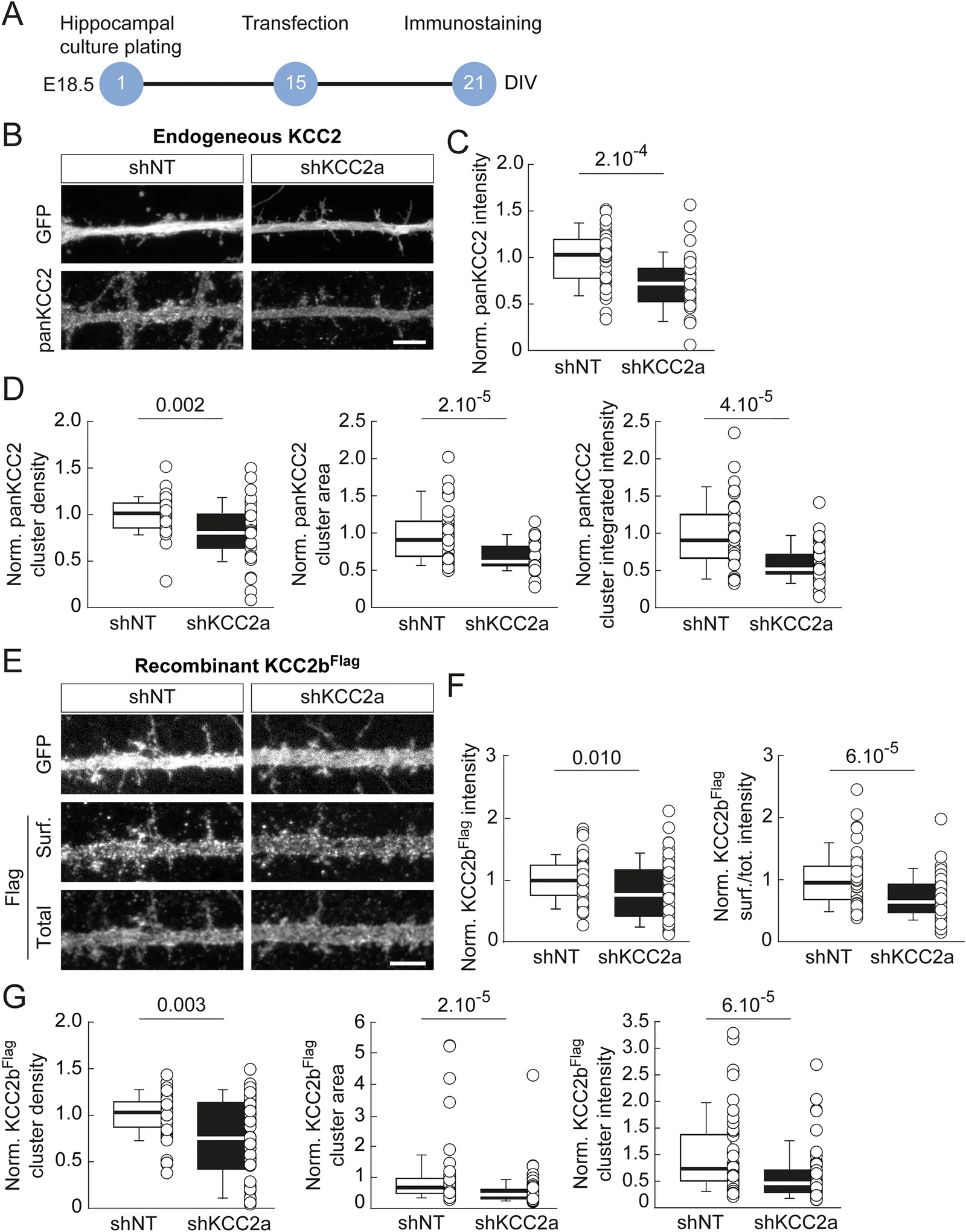
Specific KCC2a knockdown reduces KCC2b protein expression, membrane expression, and clustering. (**A**) Experiment timeline. (**B**) Representative confocal maximum projection images of dendritic sections of neurons transfected with plasmids encoding scrambled (shNT) or KCC2a-specific shRNAs (shKCC2a), immunostained for endogenous KCC2 and GFP. Scale: 5 µm. (**C**) Boxplot showing the intensity of endogenous KCC2 immunofluorescence in neurons transfected with shNT or shKCC2a_1 + 3. Two-tailed unpaired *t* test. (**D**) Boxplots showing the properties of endogenous panKCC2-immunopositive clusters. Left: cluster density/10 µm^2^, middle: mean cluster surface, right: mean integrated intensity of cluster fluorescence. shNT, *n* = 35; shKCC2a, *n* = 35 cells, from three independent cultures. Mann–Whitney rank-sum tests. (**E**) Same as (**B**) with neurons expressing recombinant KCC2b-Flag and immunostained for Flag and GFP. Scale: 5 µm. (**F**) Boxplots showing the intensity of recombinant KCC2b-Flag immunofluorescence (left) and the ratio of plasmalemmal (surf.) to the overall (total) Flag immunofluorescence intensity (right). Mann–Whitney rank-sum tests. (**G**) Same as (**D**), for neurons co-transfected with recombinant KCC2b-Flag and shRNA-expressing plasmids. shNT, *n* = 53; shKCC2a, *n* = 54 cells; from four independent cultures. Mann–Whitney rank-sum tests. All data are normalized to shNT. Box plots show the median (centre line), the 25th and 75th percentiles (box bounds) and the 10th and 90th percentiles (whiskers); all individual data points, including minima and maxima, are displayed alongside the box (see “Methods”). *P* values are provided on the graphs.

**Figure EV3:**
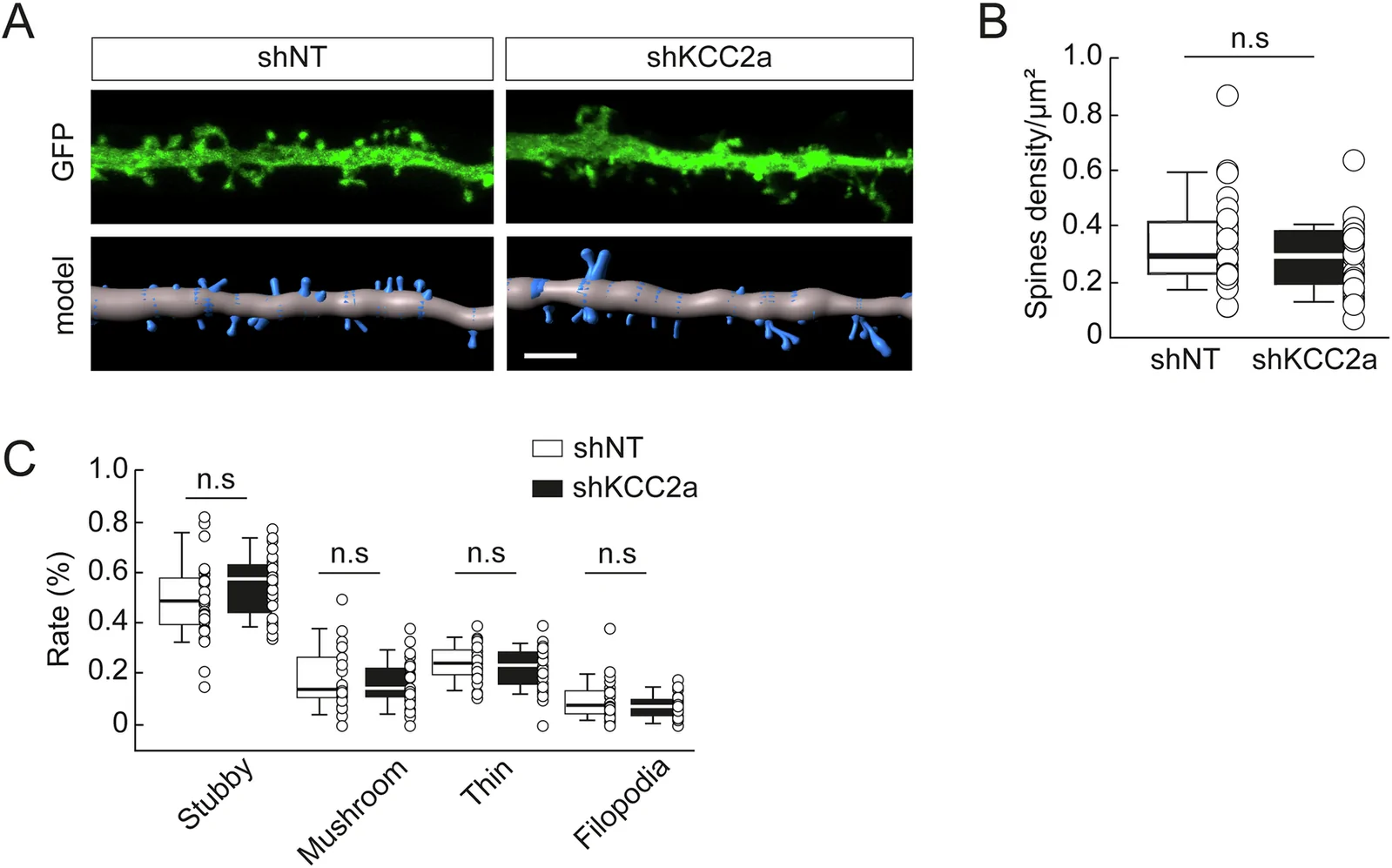
KCC2a knockdown does not affect dendritic spines in mature hippocampal neurons (related to Fig. 3). (**A**) Representative confocal maximum projection images and 3D reconstruction of dendritic sections of neurons transfected with plasmids encoding shNT or shKCC2a and immunostained for GFP. The 3D reconstruction was used for spine analysis. Scale: 3 µm. (**B**) Boxplot showing the spine density per dendrite surface area of neurons transfected with shNT or shKCC2a_1 + 3. Mann–Whitney rank-sum test. (**C**) Boxplot showing the proportion of each spine type (stubby, mushroom, long, and filopodia) within neurons transfected with plasmids encoding shNT or shKCC2a. Mann–Whitney rank-sum test, *P* value corrected for multiple comparisons using the Benjamini–Hochberg false discovery rate (FDR) procedure. Complementary linear mixed-model analyses were also performed with independent cultures as a random intercept. shNT, *n* = 28; shKCC2a, *n* = 28, from three independent cultures. Box plots show the median (centre line), the 25th and 75th percentiles (box bounds) and the 10th and 90th percentiles (whiskers); all individual data points, including minima and maxima, are displayed alongside the box (see “Methods”).

Surprisingly, despite KCC2a accounting for approximately 5% of total KCC2 mRNA, its suppression resulted in a 28% decrease in the total level of endogenous KCC2 protein (*P* = 2.10^−4^; Fig. 3A–C). This effect was accompanied by a significant decrease in the density of KCC2 clusters, as well as in their mean area and intensity (*P* ≤ 0.002 for all; Fig. 3D). We also examined whether KCC2a knockdown affects the density and morphology of dendritic spines in hippocampal neurons. We compared the spine density per surface area and the proportion of the four spine types—stubby, mushroom, long, and filopodia—between the shNT and shKCC2a conditions. No statistically significant differences in the density (*P* = 0.386) or in the proportion of each spine type (stubby: *P* = 0.326, mushroom: *P* = 0.801, long: *P* = 0.741, and filopodia: *P* = 0.624) were observed between conditions (Fig. EV3).

These results suggested that KCC2a controls the expression of KCC2b in mature hippocampal neurons. To test this hypothesis, we measured recombinant KCC2b expression upon KCC2a knockdown (Fig. 3E–G). Consistent with its effects on endogenous KCC2, KCC2a knockdown reduced the expression of recombinant KCC2b as well as its relative membrane enrichment by 20% and 30%, respectively (*P* = 0.010 and *P* = 6.10^−5^; Fig. 3F). This reduction was accompanied by a significant decrease in KCC2b cluster density, area, and fluorescence intensity (*P* ≤ 0.003 for all; Fig. 3G). Together, these data demonstrate that KCC2a exerts strong, non-stoichiometric control over KCC2b protein expression, membrane availability, and clustering.

**Figure 4:**
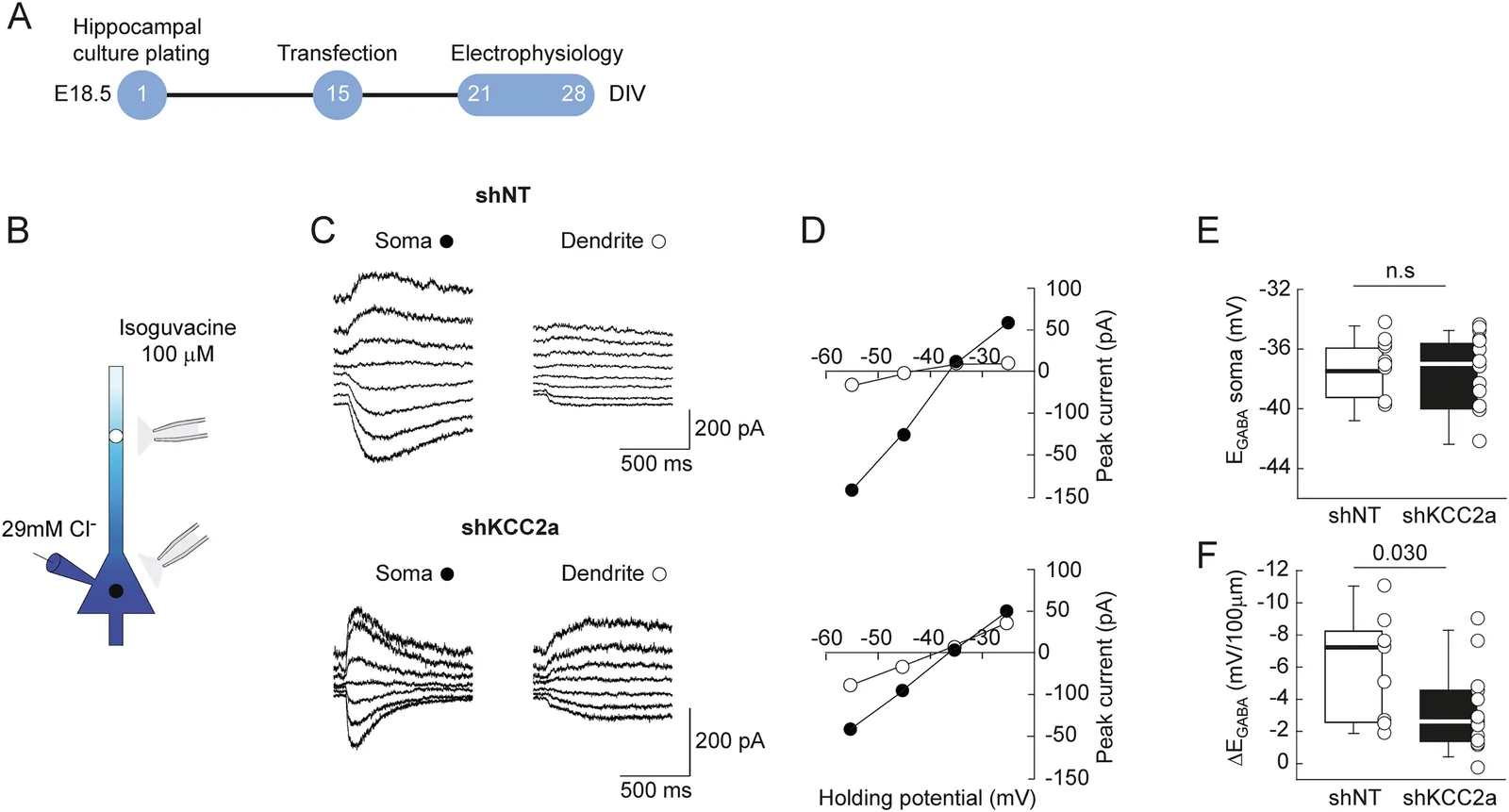
Specific KCC2a knockdown impairs chloride export in hippocampal neurons. (**A**) Experiment timeline. (**B**) Schematic representation of the experiment. Isoguvacine (100 µM) was locally released onto the soma or dendrite of neuron in whole-cell patch-clamp configuration. The patch pipette was filled with a high-chloride (29 mM) intracellular solution. (**C**) Representative recordings of isoguvacine-evoked currents at varying potentials. Currents were evoked in neurons transfected with plasmids encoding either scrambled (shNT) or KCC2a-specific (shKCC2a) shRNAs. (**D**) I–V curves corresponding to traces shown in (**C**). (**E**, **F**) Summary boxplots showing the reversal potential of isoguvacine-evoked currents at the soma (E_GABA_ soma, **E**) and the somato-dendritic E_GABA_ gradient (ΔE_GABA_, **F**) in transfected neurons expressing either shNT or shKCC2a_1 + 3. shNT, *n* = 9; shKCC2a, *n* = 14 cells; from five independent cultures. Two-tailed unpaired *t* tests. Box plots show the median (centre line), the 25th and 75th percentiles (box bounds) and the 10th and 90th percentiles (whiskers); all individual data points, including minima and maxima, are displayed alongside the box (see “Methods”). *P* values are provided on the graphs.

Since membrane stability and clustering are essential for KCC2 ion transport function (Chamma et al, 2012; Chamma et al, 2013; Côme et al, 2019), we investigated whether KCC2a regulates transmembrane chloride transport in hippocampal neurons. We assessed neuronal chloride extrusion efficiency by measuring the difference in the GABA_A_ R reversal potential (E_GABA_) between the soma and the dendrite (ΔE_GABA_) (Al Awabdh et al, 2022; Gauvain et al, 2011; Khirug et al, 2008) (Fig. 4A–D). As expected, KCC2a knockdown did not significantly impact somatic E_GABA_ (*P* = 0.755; Fig. 4E). However, it resulted in a 41% decrease in ΔE_GABA_ (6.0±1.1 versus 3.2±0.7 mV/100 μm; *P* = 0.030; Fig. 4F), indicating a significant reduction in KCC2-mediated chloride export along dendrites. Taken together, our results demonstrate the unsuspected role of the minor KCC2a isoform in controlling both KCC2b membrane expression and function in mature hippocampal neurons.

### KCC2a stabilizes KCC2b by hindering clathrin-mediated endocytosis and proteasomal degradation

**Figure 5:**
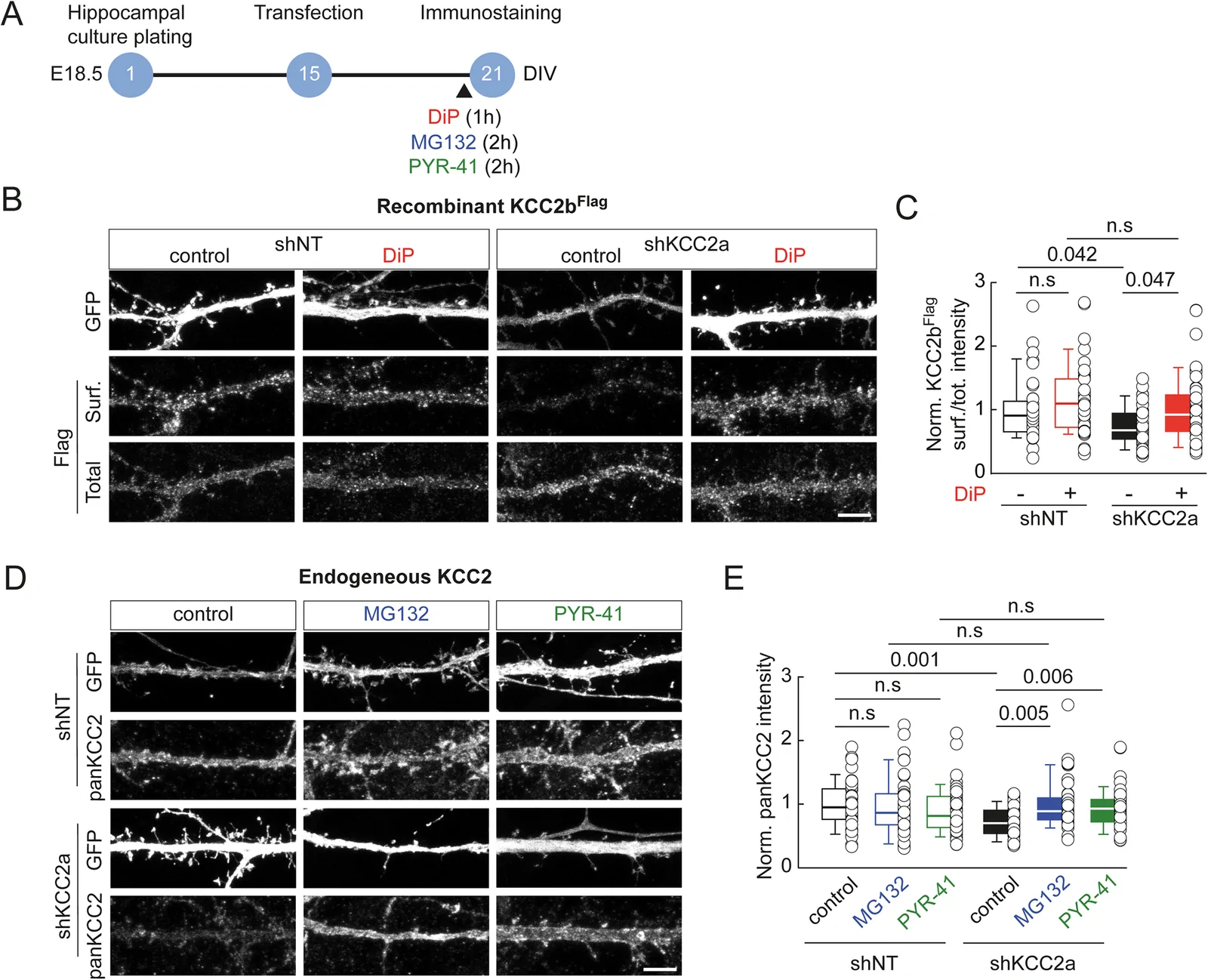
KCC2a prevents KCC2b endocytosis and degradation via the proteasomal pathway. (**A**) Timeline of the experiments. (**B**) Representative confocal maximum projection images of dendritic sections of mature hippocampal neurons expressing scrambled (shNT) or KCC2a-specific (shKCC2a) shRNA, treated with a dynamin inhibitory peptide (DiP, 50 µM) 40 min before and during live Flag immunostaining. Scale: 5 µm. (**C**) Summary boxplot showing the ratio of plasmalemmal (surf.) to the overall (total) KCC2b-Flag immunofluorescence intensity, normalized to control shNT condition. shNT control, *n* = 37; shNT +DiP, *n* = 39; shKCC2a control, *n* = 42; shKCC2a +DiP, *n* = 41 cells, from four independent cultures. Mann–Whitney rank-sum tests, *P* value corrected for multiple comparisons using the Holm–Šídák method. (**D**) Representative confocal maximum projection images of dendritic sections of hippocampal neurons expressing scrambled (shNT) or KCC2a-specific shRNA. Two hours before immunostaining with a panKCC2 antibody, neurons were treated with either a proteasome blocker (MG132, 2.5 µM), an inhibitor of the ubiquitin-activating enzyme (PYR-41, 50 µM), or untreated (control). Scale: 5 µm. (**E**) Summary boxplot showing the intensity of endogenous KCC2 immunofluorescence in the different conditions, normalized to shNT control. shNT control, *n* = 44; shNT +MG132, *n* = 38; shNT +PYR41, *n *= 43; shKCC2a control, *n* = 43; shKCC2a + MG132, *n* = 40; shKCC2a + PYR41, *n* = 44 cells, from four independent cultures. Mann–Whitney rank-sum tests, *P* value corrected for multiple comparisons using the Holm–Šídák method. Box plots show the median (centre line), the 25th and 75th percentiles (box bounds) and the 10th and 90th percentiles (whiskers); all individual data points, including minima and maxima, are displayed alongside the box (see “Methods”). *P* values are provided on the graphs.

Next, we explored the molecular mechanisms by which KCC2a stabilizes KCC2b in hippocampal neurons. Previous studies have shown that KCC2b undergoes constitutive internalization via a dynamin- and clathrin-dependent pathway (Chamma et al, 2013; Friedel et al, 2017; Zhao et al, 2008). Therefore, we investigated the involvement of KCC2a in KCC2b endocytosis. To this end, we blocked clathrin-dependent endocytosis using a dynamin inhibitory peptide (DiP) (Kittler et al, 2000) for 40 min prior to and during the 20-min live staining (Fig. 5A–C). In neurons expressing a scrambled shRNA, dynamin inhibition resulted in a modest, though not significant, increase in recombinant KCC2b membrane expression (*P* = 0.257; Fig. 5C). However, KCC2a knockdown significantly decreased the relative membrane expression of recombinant KCC2b (*P* = 0.042; Fig. 5C). This reduction was fully reversed in KCC2a knocked-down neurons treated with DiP (*P* = 0.257 compared to shNT+DiP; Fig. 5C). These results demonstrate that KCC2a stabilizes KCC2b at the membrane by preventing its clathrin-mediated endocytosis.

KCC2 may be subject to recycling at the plasma membrane following endocytosis (Côme et al, 2019). Alternatively, it may undergo degradation through the lysosomal (Lee et al, 2010) or ubiquitin-proteasomal pathway (Hu et al, 2023). Next, we investigated whether KCC2a may be preventing KCC2 degradation, which could account for its reduced total expression upon KCC2a knockdown (Fig. 5D-E). Under basal conditions (scrambled shRNA), specifically blocking the proteasome with MG132 (2.5 µM) or polyubiquitination with PYR-41 (50 µM) for 2 h did not significantly reduce endogenous KCC2 expression (*P* = 0.854 and 0.410 respectively) (Fig. 5E). However, the decrease in endogenous KCC2 expression after KCC2a knockdown was fully abolished when either the proteasome or polyubiquitination was blocked (*P* = 0.854 for both, compared to shNT with the corresponding drug; Fig. 5E). These results suggest that KCC2a protects KCC2b from polyubiquitination and subsequent degradation by the proteasome, a degradation pathway that becomes engaged upon KCC2a loss. The effect of MG132, which also inhibits calpain at this concentration, suggests the proteasomal pathway is primarily involved, though a contribution from calpain inhibition cannot be fully excluded.

### KCC2a couples KCC2b to the WNK-SPAK regulatory pathway

The expression and function of the plasmalemmal KCC2 pool are tightly regulated by various mechanisms, including protein-protein interactions, calpain-mediated cleavage, and the phosphorylation of key residues in its C-terminal domain (Côme et al, 2019; Hartmann and Nothwang, 2022; Kahle et al, 2013; Pressey et al, 2023). In particular, the WNK-SPAK kinase pathway modulates KCC2 membrane stability in a chloride-dependent manner through phosphorylation of its Thr1007 residue (Friedel et al, 2015; Heubl et al, 2017). KCC2a, but not KCC2b, was shown to contain a conserved binding motif for SPAK and co-immunoprecipitate with SPAK in heterologous cells (Uvarov et al, 2009). We therefore asked whether KCC2a is required for the WNK-SPAK-dependent modulation of KCC2b.

**Figure 6:**
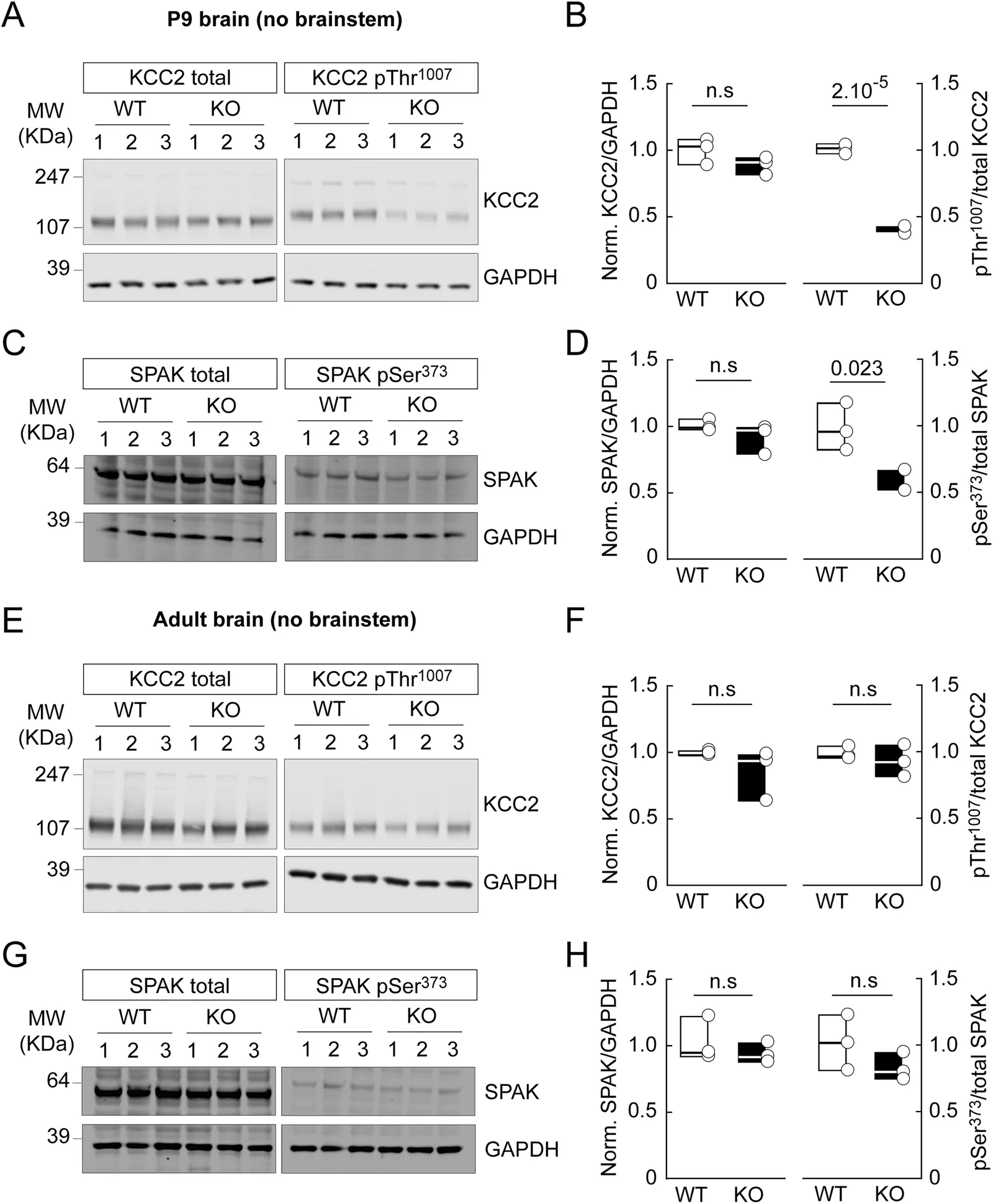
KCC2a gates WNK-SPAK-mediated KCC2 Thr1007 phosphorylation in the immature, but not adult postnatal brain. (**A**) Western blots of brain extracts (without brainstem) from P9 KCC2a-KO pups and WT littermates. Each membrane was probed for GAPDH, as well as either endogenous KCC2, KCC2 Thr^1007P^. (**B**) Summary boxplots showing the ratio of total panKCC2 immunofluorescence to that of GAPDH (left) or phosphorylated KCC2 immunofluorescence to that of GAPDH and normalized by the corresponding total protein (right). All results are normalized to WT. WT, *n* = 3; KO, *n *= 3 animals; 3 technical replicates from the same brain extracts. Two-tailed unpaired *t* test. (**C**, **D**), same as (**A**, **B**), for SPAK or hSPAK Ser^373P^ (which detect the mouse SPAK Ser^383P^) immunoblots. (**E**, **F**), Same as (**A**, **B**) for adult KCC2a-KO and WT littermates. (**G**, **H**) Same as (**C**, **D**) for adult brain extracts. WT, *n* = 3; KO, *n* = 3 animals; three technical replicates from the same brain extracts. Two-tailed unpaired *t* test. Samples being compared derive from the same experiment and membranes were processed in parallel. Box plots show the median (centre line) and the 25th and 75th percentiles (box bounds). With *n* = 3 per group, no whiskers are displayed and all individual data points are shown alongside the box. *P* values are provided on the graphs.

**Figure EV4:**
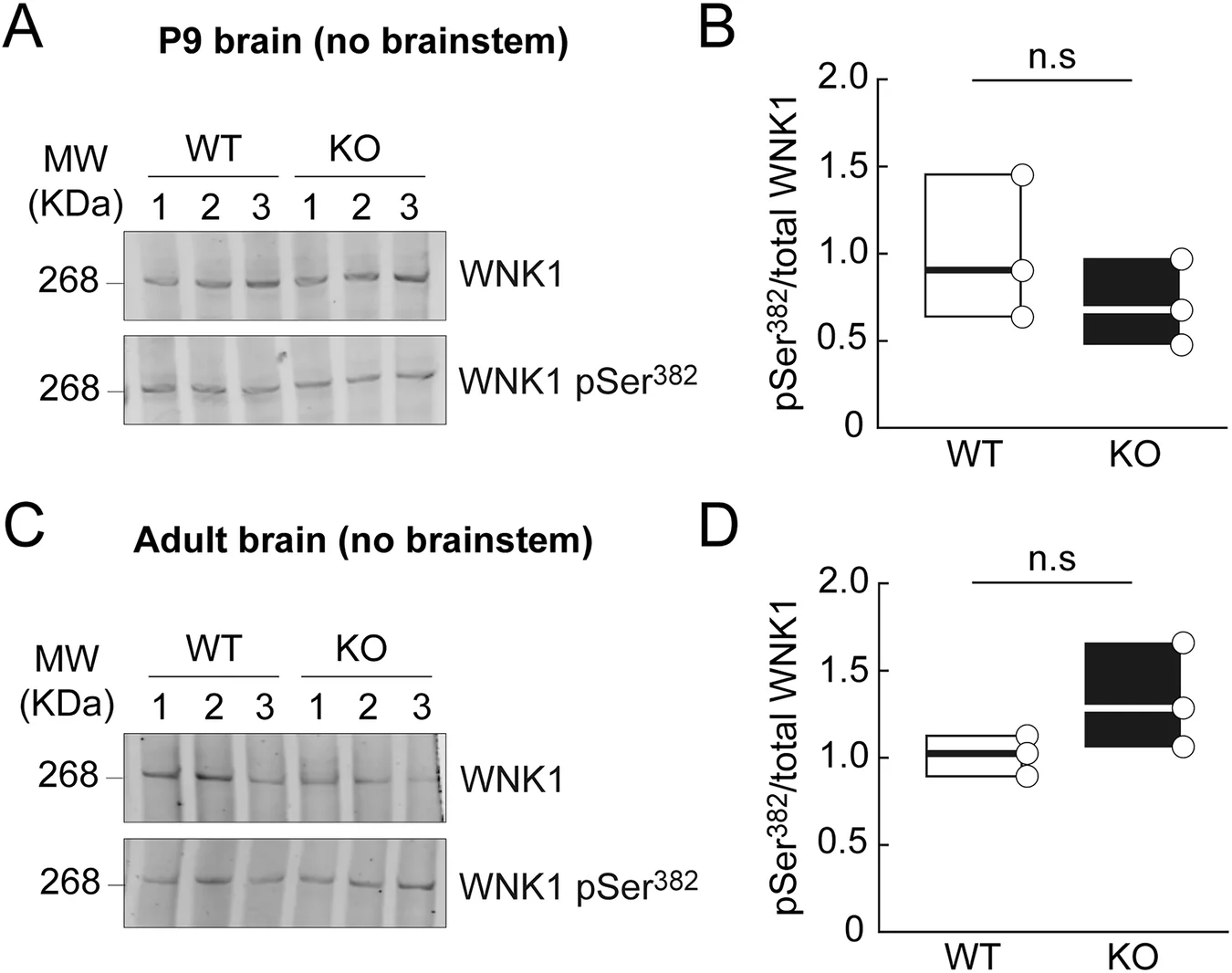
KCC2a knockout does not affect WNK1 Ser382 phosphorylation (related to Fig. 6). (**A**, **C**) Western blots of brain extracts (excluding the brainstem) from P9 and adult KCC2a-KO mice and WT littermates. Each membrane was probed for WNK1 or WNK1 Ser^382P^. Samples which are compared derive from the same experiment and membranes were processed in parallel. (**B**, **D**) Summary boxplots showing the phosphorylated WNK1 immunofluorescence intensity normalized to WNK1 in KCC2a-KO and WT mice. All results are normalized to WT. WT, *n* = 3; KO, *n* = 3 animals; 2–3 technical replicates (for P9-adult, respectively) on the same brain extracts. Two-tailed unpaired *t* test. Samples being compared derive from the same experiment and membranes were processed in parallel. Box plots show the median (centre line) and the 25th and 75th percentiles (box bounds). With *n* = 3 per group, no whiskers are displayed and all individual data points are shown alongside the box.

First, we examined protein extracts from the brains (excluding the brainstem) of KCC2a-knockout (KO) mice at postnatal day 9 (P9) (Markkanen et al, 2014), a developmental stage at which high WNK1-SPAK activity has been reported (Friedel et al, 2015). While the total level of KCC2 protein expression did not differ significantly from that of wild-type (WT) littermates (*P* = 0.182; Fig. 6A,B), KCC2a-KO mice exhibited a 62% decrease in KCC2 Thr1007 phosphorylation (*P* = 2.10^−5^; Fig. 6A,B). This suggests that KCC2a may be necessary for KCC2 phosphorylation by SPAK. Interestingly, KCC2a-KO mice also showed a 43% decrease in SPAK Ser383 phosphorylation (*P* = 0.023; Fig. 6C,D), detected with an antibody against the conserved Ser373 site from the human SPAK protein, but no changes in WNK1 Ser382 phosphorylation (Fig. EV4). This suggests that KCC2a may promote WNK1-dependent SPAK phosphorylation in the immature brain without affecting WNK1 activity itself. By contrast, no changes in KCC2 or SPAK expression or phosphorylation were observed in the brains of adult KCC2a-KO mice, in which basal WNK1-SPAK pathway activity is low (Friedel et al, 2015; Heubl et al, 2017) (*P *> 0.234; Fig. 6E–H). Together, these data show that KCC2a regulates SPAK Ser383 and KCC2b Thr1007 phosphorylation only in the immature brain.

**Figure 7:**
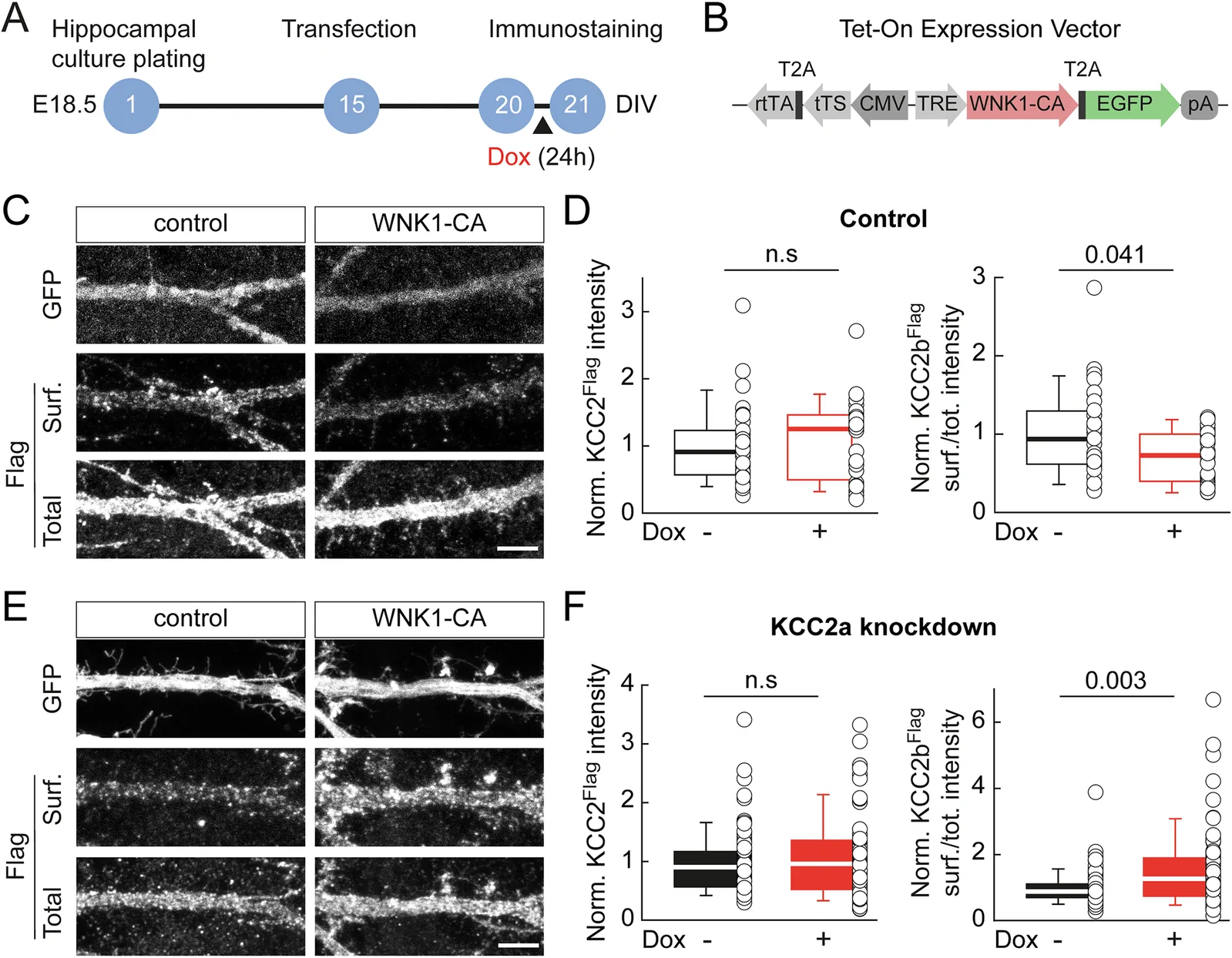
KCC2a gates WNK1-dependent regulation of KCC2b membrane expression in mature hippocampal neuron. (**A**) Experiment timeline. (**B**) Design of the plasmid encoding for the constitutively activated WNK1 (WNK1-CA), bearing a S382E mutation. (**C**) Representative confocal maximum projection images of dendritic sections of hippocampal neurons transfected with plasmids encoding GFP, WNK1-CA and recombinant KCC2b-Flag. Doxycycline (Dox + , 2 µg/ml) was added to culture medium 24 h before live immunostaining. Neurons were immunostained for Flag and GFP. Scale: 5 µm. (**D**) Summary boxplots showing the intensity of Flag immunofluorescence (left) and the ratio of plasmalemmal (surf) to the overall (total) Flag immunofluorescence intensity (right) in neurons treated or not with doxycycline. Dox-, *n* = 31; Dox + , *n* = 28 cells; from 3 independent cultures. Mann–Whitney rank-sum tests. (**E**) Same as (**C**), in neurons additionally co-transfected with plasmids encoding shKCC2a-specific shRNAs. Scale: 5 µm. (**F**) Same as (**D**), in KCC2a-specific knocked-down neurons. Dox-, *n* = 71; Dox + , *n* = 67 cells; from six independent cultures. Mann–Whitney rank-sum tests. All data were normalized to control (no doxycycline). Box plots show the median (centre line), the 25th and 75th percentiles (box bounds) and the 10th and 90th percentiles (whiskers); all individual data points, including minima and maxima, are displayed alongside the box (see “Methods”). *P* values are provided on the graphs.

Next, we investigated whether this age-dependent effect was due to different levels of KCC2a expression or WNK-SPAK activity in immature versus adult brains. To explore this possibility, we examined the impact of KCC2a knockdown on WNK-SPAK-dependent regulation of KCC2b in mature neurons under chronic activation of the WNK-SPAK pathway. We used a TetOn system, which allows for precise temporal control, to overexpress a constitutively active WNK1 bearing the S382E mutation (WNK1-CA) (Friedel et al, 2015; Inoue et al, 2012) for 24 h in mature hippocampal neurons in culture (Fig. 7A,B). In line with prior studies (Friedel et al, 2015; Heubl et al, 2017), we observed a 30% decrease in recombinant KCC2b membrane expression in mature neurons with intact KCC2a expression when WNK1-CA expression was induced by doxycycline (*P* = 0.041; Fig. 7C,D). In contrast, in KCC2a-knockdown neurons, the WNK1-CA-induced decrease in KCC2b-Flag membrane expression was abolished and even reversed, leading to a 55% increase in membrane expression (*P* = 0.003; Fig. 7E,F). This demonstrates that SPAK-dependent phosphorylation of KCC2b is tightly dependent on KCC2a, and that this effect is driven by WNK1 pathway activity rather than by the relative expression level of KCC2a.

## Discussion

In this study, we reveal the critical and previously unsuspected role of the minor KCC2a isoform in regulating the expression, membrane stability, and function of the predominant KCC2b isoform in mature hippocampal neurons. Despite accounting for less than 5% of total KCC2 mRNA in these neurons, we demonstrate that KCC2a preferentially accumulates in dendritic KCC2 clusters and exerts powerful, age-dependent, and non-stoichiometric control over KCC2b. Suppressing KCC2a in mature hippocampal neurons significantly reduces total KCC2 protein levels and impairs dendritic chloride extrusion. Mechanistically, we demonstrate that KCC2a promotes KCC2b clustering and prevents its clathrin-mediated endocytosis and subsequent proteasomal degradation. Furthermore, we establish that KCC2a provides an essential link between KCC2 and the WNK-SPAK signaling pathway, promoting KCC2b Thr1007 phosphorylation when this cascade is active. Therefore, in immature neurons, KCC2a contributes to maintaining low KCC2b membrane expression. Together, these findings revise the current model of KCC2 regulation. They identify the minor KCC2a isoform as a master regulator that controls the stability and function of KCC2 as well as the formation of a WNK1-SPAK-KCC2 complex that gates WNK1-mediated control of KCC2 phosphorylation.

Consistent with previous studies (Markkanen et al, 2017; Uvarov et al, 2007), our results indicate that KCC2a accounts for a small fraction (< 5%) of total KCC2 transcripts in mature hippocampal neurons in vivo and in primary cultures. Although it is difficult to extrapolate transcript relative abundance to protein levels, western blot data support that KCC2a may represent a similar proportion of total KCC2 in the cortex and hippocampus (Markkanen et al, 2017; Uvarov et al, 2009). Therefore, our observations raise the question of how such a weakly expressed isoform may significantly impact the membrane stability and function of the total KCC2 pool. Our data suggest that this may, at least in part, reflect the preferential targeting of KCC2a to functional KCC2 clusters. While KCC2 is expressed throughout the somato-dendritic membrane and exhibits both a diffuse and a clustered distribution, several studies have shown that KCC2 clusters likely represent the most transport-active form of the protein (Al Awabdh et al, 2022; Chamma et al, 2013; Côme et al, 2019; Gauvain et al, 2011; Watanabe et al, 2009). Indeed, manipulations of KCC2 clustering result in changes in chloride export function, even with no change in the total plasmalemmal fraction (Al Awabdh et al, 2022; Watanabe et al, 2009). Despite a slightly lower plasmalemmal-to-total ratio of recombinant KCC2a compared to KCC2b (Fig. 2D), which is consistent with previous work (Markkanen et al, 2014), our data show that endogenous KCC2a is preferentially expressed in proximal and distal dendrites compared to KCC2b (Fig. 1E). Additionally, KCC2a displays an increased cluster-to-total ratio, and the vast majority (> 70%) of KCC2 clusters contain KCC2a (Fig. 1F). Thus, despite its low relative mRNA expression level, KCC2a subcellular expression pattern may be optimal for influencing KCC2b clustering and function. Previous studies have highlighted the presence of large KCC2 clusters within dendritic spines (Chamma et al, 2013; Gauvain et al, 2011; Gulyas et al, 2001). There, KCC2 was shown to influence spine maturation and volume, actin dynamics, and synaptic function and plasticity (Awad et al, 2018; Chevy et al, 2015; Gauvain et al, 2011; Li et al, 2007; Llano et al, 2015). Remarkably, we now show that endogenous KCC2a-immunoreactivity is completely absent from dendritic spines (Fig. 1G). This suggests that the two isoforms may be differentially targeted to specific subcellular compartments and that only KCC2b may be endowed with non-canonical functions in dendritic spines.

Despite the imbalance in KCC2 isoform stoichiometry, our data reveal that KCC2a plays a critical and unexpected role in controlling KCC2 in mature hippocampal neurons. Our experiments on neurons expressing recombinant Flag-tagged KCC2b revealed trans-regulation of KCC2 isoforms: KCC2a knockdown reduced KCC2b total expression, plasmalemmal fraction, and clustering (Fig. 3), which was associated with reduced chloride export function (Fig. 4). While KCC2 was initially suggested to undergo very rapid turnover (Rivera et al, 2004), subsequent studies revealed that it is long-lived, with stability under physiological conditions ranging from hours to days (Puskarjov et al, 2012). However, KCC2 can be modulated by activity within minutes. This modulation involves the phosphorylation or dephosphorylation of key residues in its C-terminal domain or calpain cleavage. These changes result in increased membrane diffusion, endocytosis, and degradation by the lysosomal and proteasomal pathways (Côme et al, 2019; Hu et al, 2023). Our data show that, under basal conditions, blocking clathrin-dependent endocytosis for 1 h or polyubiquitination and proteasomal degradation for 2 h has no significant impact on the plasmalemmal fraction or total expression of KCC2, respectively (Fig. 5). These results are inconsistent with previous observations of rapid recycling of membrane-inserted KCC2 (Lee et al, 2010; Zhao et al, 2008), but they do confirm the relative stability of the total KCC2 pool within this time range. However, we demonstrate that these manipulations completely reverse the effects of chronic KCC2a knockdown. This suggests that KCC2a acts to promote KCC2 membrane stability and prevent its ubiquitination and proteasomal degradation, even under basal activity conditions. The molecular determinants of this stabilizing effect remain to be established, but they could involve KCC2 interactions with its numerous partner proteins (Al Awabdh et al, 2022; Mahadevan et al, 2017; Smalley et al, 2020). In particular, the proteasomal pathway has recently been identified as a key element in KCC2 degradation, involving the direct interaction of KCC2b with FBXL4, a protein that forms the E3 ubiquitin ligase complex SCF-FBXL4 and that is essential for KCC2 polyubiquitination (Chen et al, 2025; Hu et al, 2023). KCC2a may hinder this interaction, thereby preventing KCC2b ubiquitination and degradation. Alternatively, KCC2a may influence KCC2b interaction with partners known to control its membrane stability and/or clustering, such as gephyrin (Al Awabdh et al, 2022), SNARE proteins (Asraf et al, 2022), Neto2 (Ivakine et al, 2013), and CKB (Inoue et al, 2006).

In heterologous cells, KCC2a but not KCC2b, has been shown to interact with SPAK (Markkanen et al, 2017), an effector of WNK and a major regulator of cation-chloride cotransporters (Alessi et al, 2014). This interaction is consistent with the presence of a conserved SPAK/OSR1-binding motif (RFxV/I) in the N-terminal domain of KCC2a but not KCC2b (Uvarov et al, 2007). Accordingly, SPAK suppresses chloride transport mediated by recombinant KCC2a but not KCC2b (Markkanen et al, 2017). Since the two isoforms can oligomerize (Uvarov et al, 2009), KCC2a may be necessary for SPAK-dependent modulation of KCC2b within heteromeric transporters. Our results now provide direct evidence for this model. Endogenous phosphorylation of KCC2 at Thr1007 was strongly reduced in brain extracts from P9 KCC2a-KO pups (Fig. 6B), a developmental stage at which WNK-SPAK activity is high (Friedel et al, 2015). Furthermore, KCC2a knockdown abolished the WNK1-CA-induced downregulation of recombinant KCC2b membrane expression in mature hippocampal neurons (Fig. 7), demonstrating that this effect is not strictly age-dependent but persists whenever the WNK-SPAK pathway is active. Conversely, the developmental decline in WNK-SPAK activity is itself sufficient to convert KCC2a from a negative regulator—promoting KCC2b Thr1007 phosphorylation and destabilization—to a stabilizing partner that prevents its endocytosis and degradation.

Although the KCC2a-gated WNK-SPAK-dependent control of KCC2b membrane expression might seem redundant with the already low transcriptional levels of the isoform in immature neurons, these mechanisms might enable an independent regulation of KCC2 canonical and non-canonical functions (Chevy et al, 2020). Indeed, KCC2b is structurally essential during embryonic and early post-natal development as it regulates spine development as well as the apoptosis of cortical projection neurons (Li et al, 2007; Llano et al, 2015; Mavrovic et al, 2020). Yet, the maintenance of a depolarizing effect of GABA on the membrane potential at these stages is important for synaptogenesis and for the proper establishment of neuronal connections (Caillard et al, 1999; Cancedda et al, 2007; Wang and Kriegstein, 2008). Therefore, the weak KCC2b expression might enable the protein to play its structural role while its KCC2a-mediated regulation by the WNK-SPAK pathway might ensure the inhibition of its transporter function.

More surprisingly, KCC2a-KO brain extracts also showed reduced SPAK phosphorylation at Ser383 without changes in WNK1 phosphorylation (Figs. 6 and  EV4), suggesting that interaction with KCC2a may facilitate WNK1-dependent SPAK activation. Collectively, these data support a model in which KCC2a is essential for assembling a functional signaling unit comprising KCC2b and the regulatory kinase SPAK. The mechanistic basis for this model is the N-terminal SPAK/OSR1-binding motif itself. This class of motif is the established determinant of SPAK/OSR1 recruitment across the cation-chloride cotransporter family: transporters carrying it (KCC3, NKCC1, NKCC2) bind these kinases, whereas those lacking it do not (Piechotta et al, 2002). The motif tolerates conservative substitutions, including histidine for arginine at the first position (Austin et al, 2014), and its functional necessity is directly demonstrated by structural and biochemical work showing that an N-terminally truncated human KCC1 lacking this motif (KCC1Δ19) is no longer phosphorylated at its canonical regulatory threonines (Chi et al, 2021). Our data are fully consistent with this framework: in the heteromeric transporter, KCC2a supplies in trans the docking site that KCC2b lacks, allowing SPAK to access and phosphorylate KCC2b; without KCC2a, this docking site is lost. Nonetheless, direct biochemical isolation of the endogenous KCC2a-SPAK-KCC2b complex from native tissue remains challenging, as neuronal SPAK is expressed at low levels and engages a very large interactome (Delpire and Gagnon, 2007), of which KCC2 represents only a minor fraction.

Given the versatile and fine-tuning role of KCC2a in regulating KCC2b, it is somewhat surprising that KCC2a KO mice were reported to display only a mild phenotype, with a low breathing rate and abnormal occurrence of apnea at birth (Dubois et al, 2018). Our experiments using chronic KCC2a knockdown in vitro predict a reduced KCC2 expression and function in the adult forebrain of KCC2a KO mice, which are expected to impact neuronal function and cortical rhythmogenesis (Goutierre et al, 2019; Simonnet et al, 2023). In support of a specific role for this isoform in pathology, KCC2a was also found to be specifically reduced in brain samples of patients with Rett syndrome (Hinz et al, 2019). However, compensatory mechanisms may be at play in the constitutive KO mice to mask these effects. Further phenotypic exploration of these mice may be needed to reveal anomalies related to altered KCC2 function. Our data, on the other hand, also predict that acute neuronal stress induced under conditions associated with re-activation of the WNK-SPAK pathway - such as *status epilepticus* (Heubl et al, 2017), stroke (Bhuiyan et al, 2022), or brain trauma (Gong et al, 2021) - may differentially affect WT versus KCC2a KO mice. Since KCC2a appears to gate WNK-SPAK-dependent KCC2 downregulation, KO mice may display increased resilience or a better outcome in these conditions and compounds that specifically prevent KCC2a-SPAK interaction may represent useful adjunct therapeutics. Notably, small molecules that enhance KCC2 clustering and function have recently shown antiseizure efficacy in both human mTLE resective tissue and animal models (Donneger et al, 2026), underscoring the broader therapeutic potential of targeting KCC2 regulation. Importantly, because KCC2a loss also lowers baseline KCC2b function in mature neurons, the net effect of KCC2a disruption is likely to differ between acute seizure susceptibility and long-term epileptogenesis, and remains to be tested directly.

## Methods

**Table:** Reagents and tools table

Reagent/resource	Reference or source	Identifier or catalog number
**Experimental models**		
Rj:SD (*R. Norvegicus*)	Janvier	
C57BL/6JOlaHsd (*M. musculus*)	Janvier	
HEK-293 cells (*H. sapiens*)	ATCC	Cat# CRL-1573
**Recombinant DNA**		
Flag-tagged rat full-length KCC2b	Our lab	Chamma et al, 2013
Flag-tagged rat full-length KCC2a	Our lab	Synthesized for this study
p[miRNA30]-eGFP-scramble	VectorBuilder	VB210729-1210gkw
p[miRNA30]-eGFP-shKCC2a_1	VectorBuilder	VB210729-1220zyz
p[miRNA30]-eGFP-shKCC2a_3	VectorBuilder	VB210729-1219wqx
TRE > WNK1-rev(CMV>tTS:t2A:rtTA)	VectorBuilder	VB230503-1035kvj
**Antibodies**		
Rabbit anti-KCC2	Millipore	Cat# 07-432; RRID: AB_310611
Rabbit anti-KCC2a	Airaksinen lab	Markkanen et al, 2014
Mouse anti-Flag M2	Sigma	Cat# F3165; RRID: AB_259529
Chicken anti-GFP	Chemicon	Cat# AB16901
Rabbit anti-VGLUT1	Synaptic System	Cat# 135303
Rabbit anti-VGAT	Synaptic System	Cat# 131002
Donkey anti-Rabbit Cy3	Jackson ImmunoResearch	Cat# 711-165-152
Donkey anti-Rabbit A647	Jackson ImmunoResearch	Cat# 711-605-152
Donkey anti-Mouse Cy3	Jackson ImmunoResearch	Cat# 715-165-150
Donkey anti-Mouse A647	Jackson ImmunoResearch	Cat# 715-605-150
Donkey anti-Chicken A488	Jackson ImmunoResearch	Cat# 703-545-155
Rabbit anti-KCC2	Upstate	Cat# 07-432
Rabbit anti-KCC2pT1007	Phosphosolutions	Cat# p1551-1007; RRID: AB_2716769
Sheep anti-SPAK	MRC PPU reagents Dundee	sheep n° S551D
Sheep anti-SPAKpS373	MRC PPU reagents Dundee	sheep n° S670B
Rabbit anti-GAPDH	Invitrogen	Cat# PA1-987; RRID: AB_2107311
Sheep anti-WNK1	MRC PPU reagents Dundee	sheep n°S650C
Sheep anti-WNK1pS382	MRC PPU reagents Dundee	sheep n°S099B
Goat anti-rabbit IRDYE 800CW	LI-COR	Cat# 926-32211; RRID: AB_621843
Donkey anti-sheep 800	Rockland	Cat# 613-745-168
**Oligonucleotides and other sequence-based reagents**		
Random primers	Thermo Fisher Scientific	Cat# 48190011
**Chemicals, enzymes and other reagents**		
0.25% trypsin	Gibco	Cat# 15090046
HBSS	Gibco	Cat# 14175095
HEPES	Thermo Fisher Scientific	Cat# 15630056
Poly-D,L-ornithine	Sigma-Aldrich	Cat# P8638
MEM	Gibco	Cat# 51200038
horse serum	Gibco	Cat# 26050088
L-glutamine	Thermo Fisher Scientific	Cat# 25030081
Sodium pyruvate	Thermo Fisher Scientific	Cat# 11360039
Neurobasal medium	Gibco	Cat# 21103049
B27	Thermo Fisher Scientific	Cat# A3582801
Penicillin–streptomycin	Gibco	Cat# 15140122
DMEM + GlutaMAX + pyruvate	Gibco	Cat# 10569010
FBS	Gibco	Cat# A3160801
TransFectin	Bio-Rad	Cat# 1703351
PEI	Sigma-Aldrich	
D-glucose	Gibco	
DynaMin Inhibitory Peptide, myristoylated	MedChemExpress	Cat# HY-P1369
PYR-41	MedChemExpress	Cat# HY-13296
MG132	MedChemExpress	Cat# HY-13259
Doxycycline hyclate	Sigma-Aldrich	Cat# D5207
Triton X − 100	Carl Roth	Cat# 3051.3
Goat serum	Gibco	Cat# 16210064
DAPI	Sigma-Aldrich	Cat# D9542
TRIzol	Invitrogen	Cat# 15596026
RNAqueous-Micro Kit	Ambion	Cat# AM1931
SuperScript II reverse transcriptase	Invitrogen	Cat# 18064014
ABsolute Blue QPCR Mix, SYBR Green	Thermo Fisher Scientific	Cat# AB4163A
Protease inhibitors	Roche	Cat# 11836170001
Phosphatase inhibitors	Thermo Fisher Scientific	Cat# 78420
PierceTM BCA Protein assay	Thermo Fisher Scientific	Cat# 23227
NuPAGE™ Sample reducing agent (10X)	Invitrogen	Cat# NP0009
NuPAGE™ LDS Sample Buffer (4X)	Invitrogen	Cat# NP0007
NuPAGE™ Bis-Tris Mini Protein Gels, 4–12%	Invitrogen	Cat# NP0323BOX
NuPAGE™ Tris acetate Mini Protein Gels	Invitrogen	Cat# EA0375BOX
Amersham Protran NC Nitrocellulose Membrane	Thermo Fisher Scientific	Cat# 10600002
TTX	Latoxan	Cat# L8502
D-AP5	HelloBio	Cat# HB0225
NBQX	HelloBio	Cat# HB0443
CGP54626	Tocris Bioscience	Cat# 1088
Isoguvacine	HelloBio	Cat# HB0883
**Software**		
SigmaPlot 13	SYSTAT Software	
ImageJ	NIH	
Imaris 10 and 11	Oxford Instruments	
Metamorph	Molecular Devices	
LAS AF	Leica	
Leica DM6000 microscope	Leica	
RTE/CCD-1300-Y/HS MicroMAX Camera	Roper Scientific	
Leica TCS SP5 microscope	Leica	
QuantStudio 3	Thermo Fisher Scientific	
Odyssey CLx Imager	LI-COR	

### Animal experimentation

All animal procedures were carried out according to the European Community Council directive of 24 November 1986 (86/609/EEC) and the guidelines of the French Agriculture and Forestry Ministry for handling animals (decree 87-848) and were approved by the *Direction Départementale de la Protection des Populations de Paris*. All procedures were performed in a user facility (Institut du Fer à Moulin animal facility, license E750522). Female rats were bought while pregnant and were housed under standard conditions at 20-24 °C with a 12/12-h dark/light cycle and food and water provided ad libitum. Brains from male and female KCC2a-KO mice and their WT littermates were kindly provided by Muriel Thoby-Brisson’s team and were handled as in (Dubois et al, 2018).

### Primary hippocampal cultures

Primary hippocampal neuron cultures were prepared from the hippocampi of WT Sprague Dawley (Rj:SD) male and female rats on embryonic day 18.5 (E18.5), as previously described (Al Awabdh et al, 2022; Gauvain et al, 2011). The hippocampi were dissociated using 0.25% trypsin (Gibco) and then triturated mechanically in ice-cold HBSS (Gibco) supplemented with HEPES (Thermo Fisher Scientific). The neurons were plated at a density of 1.5 × 10⁵ cells per 18-mm glass coverslip pre-coated with 50 μg/ml poly-D,L-ornithine (Sigma-Aldrich). The initial plating medium consisted of minimum essential medium (Gibco) supplemented with 10% horse serum (Gibco), 2 mM L-glutamine (Thermo Fisher Scientific), and 1 mM sodium pyruvate (Thermo Fisher Scientific). After a 3 to 4-h attachment period, the plating medium was replaced with a serum-free culture medium composed of Neurobasal medium (Gibco) supplemented with B27 (Gibco), 2 mM L-glutamine (Thermo Fisher Scientific), and antibiotics (200 units/mL penicillin and 200 μg/mL streptomycin, Gibco). The neurons were maintained in an incubator at 37 °C with 5% CO₂ for up to four weeks, with one-third of the medium replaced weekly.

### HEK-293 cell cultures

HEK-293 cells were maintained in high glucose (4.5 g) DMEM supplemented with GlutaMAX and pyruvate (Gibco), 10% FBS (Gibco), and 0.2% penicillin/streptomycin (Gibco). For transfection, cells were plated at a density of 5 × 10^4^ cells per 18-mm glass coverslip in 12-well plates without antibiotics. Cells were regularly tested for mycoplasma contamination. Cells have not been authenticated.

### Plasmid constructs

The KCC2b-Flag construct was generated by inserting a sequence encoding a 3xFlag tag (DYKDHDGDYKDHHIDYKDDDD) into the second extracellular loop of KCC2, between the NsiI and NheI restriction sites of the full-length pEGFP–IRES–KCC2 construct (GenBank accession number U55816) (Chamma et al, 2013). The KCC2a-Flag construct was subcloned by inserting a synthetic 120-bp fragment encoding the KCC2a-specific N-terminus into the KCC2b-Flag backbone to replace the KCC2b-specific N-terminal coding sequence (Genscript, sequence available upon request). Specific knockdown of rat KCC2a was achieved using two distinct shRNAs (short hairpin RNAs) embedded in a miR-30 backbone obtained from VectorBuilder. The target shRNA sequences, which were designed using VectorBuilder and Thermo Fisher Scientific BLOCK-iT™ RNAi Designer tools, were: CTCCCGAGGGAAGACGTCAAA (shKCC2a_1), ACCCAGAGTCCCGCCGGCATTCGGT (shKCC2a_3). The scrambled shRNA sequence was: ACCTAAGGTTAAGTCGCCCTCG.

The WNK1-CA construct expresses a constitutively active form of rat WNK1 with an S382E mutation, which is cloned into a Tet-On backbone (TRE > WNK1:T2A:GFP-rev(CMV>tTS:t2A:rtTA); VectorBuilder). Co-expression of WNK1 and GFP is controlled by the doxycycline-inducible TRE promoter. Doxycycline facilitates binding of the rtTA transcriptional enhancer and prevents binding of the tTS transcriptional silencer, thus activating gene expression. A GFP-encoding construct was used as a control to assess transfection efficiency.

### Transfection procedures

Hippocampal neurons were transfected between days in vitro (DIV) 13 and 14 using TransFectin (Bio-Rad), following the manufacturer’s instructions. Briefly, the appropriate plasmid combinations (see Table EV1) were mixed in complete culture medium to achieve a total plasmid mass of 1 μg per 20-mm well. Then, TransFectin was mixed with the complete medium at a ratio of 3 μl of TransFectin per 1 μg of plasmid. The plasmid- and TransFectin-containing media were then combined and added to the neurons for 45 min. Subsequently, they were replaced with a mixture of conditioned (2/3) and fresh (1/3) complete culture medium. The transfected neurons were used for immunocytochemistry or electrophysiological analysis 7 to 10 days post-transfection.

HEK-293 cells at 70% confluence were transfected using polyethyleneimine (PEI, Sigma-Aldrich) following a protocol similar to that for neuronal transfection. The cells were incubated in the plasmid/PEI mixture for 48 h prior to fixation.

### Pharmacological treatments

Clathrin-mediated endocytosis was inhibited by incubating neurons for 40 min with the Dynamin Inhibitory Peptide, myristoylated (DiP) (50 μM, MedChemExpress) in complete culture medium at 37 °C. Because the effects of DiP are reversible, it was maintained in the medium for all subsequent live staining procedures (20 min).

The ubiquitin-proteasome system was blocked by incubating neurons for 2 h at 37 °C with either the ubiquitin-activating enzyme (E1) inhibitor PYR-41 (50 μM, MedChemExpress) or the proteasome inhibitor MG132 (2.5 μM, MedChemExpress) in complete culture medium. As MG132 is a known calpain inhibitor (IC_50_  = 1.2 μM), this treatment condition also served to inhibit calpain-dependent degradation of KCC2.

Expression of the constitutively active WNK1 (WNK1-CA) via the Tet-On system was induced by incubating neurons for 24 h with doxycycline hyclate (2 μg/ml, Sigma-Aldrich) in complete culture medium at 37 °C.

### Immunocytochemistry

To ensure the specificity of the in-house rabbit anti-KCC2a antibody (Markkanen et al, 2014), antibodies were pre-adsorbed on brainstems from P9 KCC2a-KO mice. Tissue was fresh-frozen, fixed in 4% PFA overnight at 4 °C, and sectioned at 50 μm. Approximately forty sections were permeabilized with 0.25% Triton X − 100 (Carl Roth) and incubated for 3 days at 4 °C with 1 ml of the rabbit anti-KCC2a primary antibody (1:750) in 3% inactivated goat serum under agitation. The resulting collected antibody solution was subsequently used for KCC2a immunocytochemistry experiments on cultured neurons.

To quantify total endogenous KCC2a and KCC2 expression, the cells were fixed in a solution of 4% paraformaldehyde and 4% sucrose for 15 min. The neurons were then permeabilized with 0.25% Triton X-100 and blocked for 30 min in 10% inactivated goat serum (Gibco). A two-step primary/secondary antibody staining protocol was then used sequentially. First, the coverslips were incubated with rabbit anti-KCC2a primary antibody (dilution as in Table EV2) in 3% inactivated goat serum for 1 h at room temperature. After washing with 1× PBS, the cells were incubated with a Cy3-conjugated donkey anti-rabbit secondary antibody solution for 45 min at room temperature. The process was then repeated using a rabbit anti-KCC2 antibody and a A647-conjugated donkey anti-rabbit secondary antibody. The same protocol—without the rabbit anti-KCC2/donkey anti-rabbit A647 step—was used to quantify endogenous KCC2a alone to test the efficacy of anti-KCC2a shRNAs.

To quantify the relative membrane expression of recombinant KCC2 isoforms, a live-immunostaining protocol was used as previously described (Al Awabdh et al, 2022). Living neurons were incubated with mouse anti-flag M2 primary antibodies in MEM medium (Gibco) supplemented with D-Glucose (33 mM), sodium pyruvate (1 mM), HEPES (20 mM), glutamine (2 mM), and B27 (1X) to tag the membrane-inserted KCC2-Flag protein (surface) at 13 °C for 20 min. The cells were then fixed, permeabilized and blocked as previously described. After washing with 1x PBS, the cells were sequentially incubated with: a Cy3-conjugated donkey anti-mouse antibody, a mouse anti-Flag M2 and chicken anti-GFP antibody solution and finally a solution containing A647-conjugated donkey anti-mouse and A488 donkey anti-chicken secondary antibodies. The cells were then washed, DAPI-stained, and mounted. For experiments quantifying total endogenous KCC2 or recombinant Flag-tagged KCC2 isoforms, the cells were fixed, permeabilized, and blocked as described above. Primary antibodies (combination as in Table EV2) were incubated for 1 h at room temperature in 3% inactivated goat serum. After washing with 1× PBS, the cells were incubated with the appropriate secondary antibody solutions (Table EV2) for 45 min at room temperature. The cells were then washed, DAPI-stained, and mounted.

### Image acquisition and analysis

Standard fluorescence quantification images were acquired using a Leica DM6000 epifluorescence microscope with a 63x objective (NA 1.40) and a 12-bit cooled CCD camera (Micromax, Roper Scientific). The microscope was controlled using Metamorph software (Roper Scientific). The exposure time and other acquisition parameters were determined for each fluorescent channel using the brightest cells to prevent saturation, and these parameters were kept constant across all cells within a given experimental replicate. ImageJ software was used to quantify the surface-to-total fluorescence ratio of recombinant KCC2 isoforms. A region of interest (ROI) was drawn around a primary dendrite positioned in the focal plane. Mean fluorescence intensity was then measured sequentially for the surface (Cy3) and total (Alexa647) pools. Background mean intensity was measured separately for each fluorophore and subtracted from the corresponding signal intensity within the ROI to yield the corrected fluorescence intensity.

Image acquisition for experiments including endogenous KCC2a expression and KCC2 expression depending on various treatments was performed using a Leica SP5 confocal microscope with a 63x objective (NA 1.40) and LAS AF software. Confocal images were acquired at a zoom level of 5. For each neuron, a Z-stack was acquired across a primary dendrite with 0.5 μm Z-steps. For analysis, the Z-stacks were processed into a sum projection using ImageJ. A primary dendrite was selected, and an ROI was drawn. Background fluorescence was subtracted from the A647 signal to determine total protein expression. To account for dendrite thickness, the value was normalized by the dendrite thickness, which was calculated as [number of images in stack − 1] × 0.5 μm. Relative surface expression was calculated as the ratio of corrected surface intensity to corrected total intensity.

We analyzed panKCC2 and KCC2a expression along the somato-dendritic axis using Z-axis projected images. The number of planes was adjusted depending on whether somatic or dendritic expression was quantified. Background intensity was measured independently for the soma stack and the dendrite stack to ensure accurate subtraction. Normalization by the thickness of the neuron ([number of images in the stack – 1]*0.5) was performed independently at the soma and at the dendrite.

IMARIS software was used to quantify enrichment of panKCC2 and KCC2a in protein clusters. Dendritic segments were visualized in 3D and isolated from background noise. Aggregates with a diameter greater than 0.7 μm and a fluorescence intensity exceeding a user-defined threshold were classified as clusters and modeled as 1-μm spheres. The number of clusters and the fluorescence intensity within the clusters and within the dendritic portion were measured for both isoforms, and the corresponding background values were subtracted.

Analysis of KCC2 and recombinant KCC2b-Flag clusters was performed using an in-house Metamorph routine as previously described (Al Awabdh et al, 2022). A primary dendrite ROI was selected, and images were digitally flattened and filtered. Clusters were detected using a user-defined intensity threshold and a minimum area of 0.031 µm^2^. The cluster regions were superimposed on the raw images to calculate their mean density, area, and integrated intensity.

IMARIS software was used to quantify spines type and density. Dendritic segments were visualized in 3D and isolated from background noise. The isolated dendrite was defined as a filament and spines (diameter >0.3 µm, length <5 µm) were defined from this filament using a threshold method based on the *Shortest Distance from Distance Map* algorithm. Spines that were not detected with this technique were added manually and then processed with the previously defined spines. The number of spines per dendrite surface unit was measured. Spines were classified using IMARIS Classify Spines XTension. We compared the proportions of the four dendritic spine types (stubby, mushroom, long, filopodia) using a custom Python script with Mann–Whitney *U* tests. We corrected the *P* values for multiple comparisons using the Benjamini–Hochberg false discovery rate (FDR) procedure. Complementary linear mixed-model analyses were also performed with independent cultures as a random intercept.

### RNA extraction

RNA was extracted from rat brain tissue using TRIzol reagent (Invitrogen). The tissue was snap-frozen on dry ice and stored at −80 °C. The tissue was lysed by immersing it in 1 ml of TRIzol reagent and homogenizing it with a Potter homogenizer. Then, 200 μl of chloroform was added, and the mixture was centrifuged to separate the aqueous phase containing the RNA. The RNA was then precipitated by adding 500 μl of isopropanol, followed by centrifugation for 10 min. The RNA pellet was washed with 75% ethanol, air-dried for 10 min, and resuspended in 50 μl of RNase-free water.

RNA was extracted from cultured neurons using the RNAqueous-Micro Kit (Ambion). Neurons were plated in 6-well plates at a density of 3.5 × 10⁵ cells/well. For each time point (DIV 7, 10, and 24), neurons from two wells were harvested and pooled. The cells were detached by mechanical dissociation and centrifuged. The supernatant was discarded, cells were lysed, and 50 μl of absolute ethanol was added to the lysate. The lysate/ethanol mixture was loaded onto a column provided in the kit, followed by a series of washing and centrifugation steps. RNA was eluted in 20 μl elution buffer. Residual genomic DNA was removed by DNase I treatment.

### Reverse transcription and quantitative PCR

Total RNA was reverse transcribed using SuperScript II reverse transcriptase (Invitrogen). The reaction mixture contained 1 μg RNA, 100 ng random primers (Thermo Fisher Scientific), and 1 μl of a 10 mM dNTP mix (Thermo Fisher Scientific) and was brought to 13 μl with sterile water. The mixture was denatured at 65 °C for 5 min. DTT was added, followed by a 2 min incubation at RT, and then 1 μl SuperScript reverse transcriptase was added. The synthesis steps were 10 min at 25 °C and 50 min at 42 °C. The reaction was then inactivated with a 15-min incubation at 70 °C.

Quantitative PCR was conducted using the ABsolute Blue QPCR Mix, SYBR Green, ROX (Thermo Fisher Scientific). Each target amplification (KCC2a, KCC2b, and the housekeeping gene Ywhaz) was run in triplicate in a 384-well qPCR plate, alongside a no-reverse-transcriptase (NRT) control and a water control. Each 10 μl reaction consisted of: 5 μl Tp2X Sybr, 4 μl template cDNA (C = 10 ng/μl), 0.2 μl forward and reverse primer (10 μM) (Table EV3), and 0.8 μl water. qPCR was performed on a QuantStudio5 device using the following thermal cycling program: initial denaturation at 95 °C for 10 min, followed by 40 cycles of 95 °C for 30 s, 58 °C for 30 s, 72 °C for 20 s, and a final melt curve step at 95 °C for 15 s. *Ywhaz* encodes the 14-3-3ζ protein (tyrosine 3-monooxygenase/tryptophan 5-monooxygenase activation protein ζ) and was selected as reference gene for its high abundance in brain and its established suitability for qPCR normalization (Bonefeld et al, 2008); in our hands, its expression remained stable across developmental stages in both ex vivo tissue and neuronal cultures, unlike actin.

The RT-qPCR data were analyzed as follows. For each condition (rat hippocampus/cortex, hippocampal neurons in culture), 3–4 independent experiments were performed. Within a single experiment, each variant (*KCC2a*, *KCC2b*, *Ywhaz*) was analyzed at different times (E18/adult or DIV7/10/24) and each association of variant and time was run in triplicate. We calculated the mean Ct of the triplicates for each variant-time, and the value of 2^-ΔΔCt^ was calculated by subtraction of a variant mean Ct by *Ywhaz* mean Ct and then by normalization either by KCC2b value or by the E18/DIV7 value. Finally, we calculated the average 2^-ΔΔCt^ of the independent experiments for each variant-time.

### Western blot

Brains used for the western blots were obtained by fast cervical dislocation and decapitation. They were then dissected out, snap-frozen by immersion in isopentane and stored at −80 °C. Adult brain hemispheres or P9 whole brains (with brainstem dissected out) from KCC2a-KO C57BL6/J mice and WT littermates were lysed in a modified RIPA buffer containing (in mM): 50 Tris-HCl (pH 7.4), 150 NaCl, 1% Nonidet P − 40, 1% Triton X-100, 0.5% DOC, 0.1% SDS, 50 NaF, 1 EDTA supplemented with 1X protease inhibitors (Roche) and phosphatase inhibitors (Thermo Fisher Scientific). The lysis volume was standardized (10 μl RIPA per 1 mg tissue). Lysis was achieved by homogenization in a Potter homogenizer. Samples were centrifuged, and the supernatant containing the protein lysate was collected. Protein concentration was determined using the Pierce^TM^ BCA Protein assay (Thermo Fisher Scientific).

For KCC2 and SPAK western blots, protein extracts were prepared with sample reducing agent (Invitrogen) and LDS sample buffer (Invitrogen) to a final concentration of 3 μg/μl and denatured for 1 h at 37 °C. In total, 10 μl of sample was loaded onto a 4–12% Bis-Tris polyacrylamide gel (Invitrogen) for electrophoresis. Proteins were transferred to a nitrocellulose membrane for 3 h at 100 V at 4 °C. Membranes were washed (3 × 10 min) with TBS-Tween (TBST) and blocked with 5% (w/v) skim milk. Primary antibodies were incubated overnight at 4 °C in 5% (w/v) skim milk solution: rabbit anti-KCC2 (1:1000, Upstate), rabbit anti-KCC2pT1007 (1:1000, PhosphoSolutions), sheep anti-SPAK (1:50, MRC PPU reagents Dundee), sheep anti-hSPAKpS373 (1:50, MRC PPU reagents Dundee) and rabbit anti-GAPDH (1:2000, Invitrogen) as loading control. Membranes were washed (3 × 10 min TBST) and incubated for 1 h at RT with goat anti-rabbit DyLight 800 secondary antibody (1:5000, LI-COR) and donkey anti-sheep 800 secondary antibody (1:1000, Rockland, for SPAK staining) in 1% (w/v) skim milk. Fluorescence was acquired using the Odyssey infrared imaging system (LI-COR Bioscience).

For WNK1 western blots, protein samples were denatured for 10 min at 70 °C. In total, 10 μl of protein was loaded on a 3–8% Tris-Acetate polyacrylamide gel (Invitrogen) and transferred to a nitrocellulose membrane for 2.5 h at 65 V at 4 °C. Immunostaining utilized sheep anti-WNK1 (1:50, MRC PPU reagents Dundee) or sheep anti-WNK1pS382 (1:50, MRC PPU reagents Dundee) primary antibodies, followed by donkey anti-sheep 800 secondary antibody (1:1000, Rockland). Integrated intensity of the different bands signal was measured using ImageJ software. To quantify KCC2 (or SPAK) expression for each animal, the integrated intensity of the KCC2 signal was divided by the integrated intensity of the corresponding GAPDH signal. To quantify KCC2 phosphorylation level for each animal, the integrated intensity of the KCC2 Thr1007 phosphorylated signal was divided by the corresponding GAPDH (1) signal. The integrated intensity of the KCC2 signal was divided by the corresponding GAPDH (2) signal. Finally, the $\frac{{KCC}2\,{Thr}10\times {GAPDH}\left(2\right)}{{KCC}2\times {GAPDH}\left(1\right)}$ ratio was calculated. To quantify WNK1 phosphorylation level for each animal, the integrated intensity of the WNK1 Ser382 phosphorylated signal was divided by the integrated intensity of the corresponding WNK1 signal. To account for technical variability, the final protein/GAPDH or phospho-protein/protein ratio for each biological sample (animal) was averaged from three independent technical replicates.

### Electrophysiology

Whole-cell patch clamp recordings were performed on DIV 21-24 hippocampal neurons superfused at a rate of 2 ml/min with an artificial cerebral fluid solution (ACSF) composed of (in mM): 2 CaCl_2_, 2 KCl, 3 MgCl_2_, 10 HEPES, 20 D-glucose, 120 NaCl at 34 °C. Neurons were recorded using 5 MOhm borosilicate glass pipettes filled with (in mM): 104 K-gluconate, 25.4 KCl, 10 HEPES, 10 EGTA, 2 MgATP, 0.4 Na_3_GTP, 1.8 MgCl_2_. The liquid junction potential (14.9 mV) was corrected post hoc. Signals were acquired with a Multiclamp 700B amplifier (Molecular Devices), low-pass filtered at 2 kHz and digitized at 20 kHz using Clampex 11 (Molecular Devices). GABA_A_R-mediated currents were isolated using (in µM): 1 TTX (Na^+^ channel blocker, Latoxan), 50 D-AP5 (NMDA receptor antagonist, HelloBio), 10 NBQX (AMPA receptor antagonist, HelloBio) and 100 CGP54626 (GABA_B_ receptor antagonist, Tocris Bioscience). After achieving whole-cell configuration, an isoguvacine-filled pipet (100 μM) was positioned near the soma. Voltage steps ranging from −80 mV to 0 mV (10 mV step) were applied, and GABAA R-mediated currents were evoked by the release of isoguvacine at each step. The isoguvacine pipet was then repositioned over a primary dendrite at a distance of 50−100 μm from the soma, and the same voltage-step protocol coupled with isoguvacine release was applied. Currents were registered at each holding potential, corrected for both the access resistance voltage drop and the liquid junction potential. Current-voltage (I–V) curves were plotted offline using Clampfit 11 to estimate the reversal potential of GABA_A_ R currents at the soma (EGABA_soma_) and the dendrite (EGABA_dendrite_). The difference (ΔE_GABA_=EGABA_dendrite_ −EGABA_soma_) was computed and then normalized by the distance between the two application sites to quantify the EGABA gradient.

### Statistical analysis and experimental study design

All statistical procedures were conducted in SigmaPlot 13, with significance set at *P* < 0.05. Data are visualized as box plots in which the centre line indicates the median, the box bounds indicate the 25th and 75th percentiles and the whiskers extend to the 10th and 90th percentiles; all individual data points, including minima and maxima, are displayed alongside the box. Numerical differences discussed in the text are based on mean values ± SEM. We determined the appropriate test by first evaluating normality (Shapiro–Wilk) and, for multi-group comparisons, equal variance (Brown–Forsythe). Two-group comparisons were conducted using two-tailed Student’s *t* test (normal distribution) or Mann–Whitney rank-sum test (non-normal). For specific experiments involving more than two groups, such as pharmacological rescue experiments (e.g., Fig. 5), *P* values were further corrected using the Holm–Šidák method. Multi-group comparisons were performed using one-way ANOVA with Tukey’s post hoc test, or Kruskal–Wallis ANOVA on ranks with Dunn’s post hoc test if assumptions were violated.

No statistical methods were used to estimate the samples size and no randomization was performed. Cultures in which neurons seemed particularly damaged were excluded from our experiments. Experiments with a clearly identified experimental issue were also excluded. Data points considered as outliers (criteria depend on the experimental paradigm) were excluded from our analysis. When possible, data were analyzed by an investigator blinded to the experimental group allocation.

## Supplementary information

Table EV1

Table EV2

Table EV3

Peer Review File

Expanded View Figures

## Data Availability

All source data, uncropped western blot images, and original microscopy images underlying Figs. 1–7 and Expanded View Figs. EV1, EV2, EV3 and EV4 have been deposited in Zenodo and are publicly available at https://doi.org/10.5281/zenodo.20762432. The source data of this paper are collected in the following database record: biostudies:S-SCDT-10_1038-S44319-026-00920-3.

## References

[CR1] Al Awabdh S, Donneger F, Goutierre M, Séveno M, Vigy O, Weinzettl P, Russeau M, Moutkine I, Lévi S, Marin P et al (2022) Gephyrin interacts with the K-Cl cotransporter KCC2 to regulate its surface expression and function in cortical neurons. J Neurosci 42(2):166–182 10.1523/JNEUROSCI.2926-20.2021 PMC8802937 34810232

[CR2] Alessi DR, Zhang J, Khanna A, Hochdörfer T, Shang Y, Kahle KT (2014) The WNK-SPAK/OSR1 pathway: master regulator of cation-chloride cotransporters. Sci Signal 7(334):re3 10.1126/scisignal.2005365 25028718

[CR3] Asraf H, Bogdanovic M, Gottesman N, Sekler I, Aizenman E, Hershfinkel M (2022) SNAP23 regulates KCC2 membrane insertion and activity following mZnR/GPR39 activation in hippocampal neurons. iScience 25(2):103751 10.1016/j.isci.2022.103751 PMC8800107 35118363

[CR4] Austin TM, Nannemann DP, Deluca SL, Meiler J, Delpire E (2014) In silico analysis and experimental verification of OSR1 kinase - peptide interaction. J Struct Biol 187(1):58–65 10.1016/j.jsb.2014.05.001 PMC4082687 24821279

[CR5] Awad PN, Amegandjin CA, Szczurkowska J, Carrico JN, Fernandes do Nascimento AS, Baho E, Chattopadhyaya B, Cancedda L, Carmant L, Di Cristo G (2018) KCC2 regulates dendritic spine formation in a brain-region specific and BDNF dependent manner. Cereb Cortex 28(11):4049–4062 10.1093/cercor/bhy198 PMC6188549 30169756

[CR6] Ben-Ari Y (2002) Excitatory actions of GABA during development: the nature of the nurture. Nat Rev Neurosci 3(9):728–739 10.1038/nrn920 12209121

[CR7] Bhuiyan MIH, Young CB, Jahan I, Hasan MN, Fischer S, Meor Azlan NF, Liu M, Chattopadhyay A, Huang H, Kahle KT et al (2022) NF-κB signaling-mediated activation of WNK-SPAK-NKCC1 cascade in worsened stroke outcomes of Ang II-hypertensive mice. Stroke 53(5):1720–1734 10.1161/STROKEAHA.121.038351 PMC9038703 35272484

[CR8] Bonefeld BE, Elfving B, Wegener G (2008) Reference genes for normalization: a study of rat brain tissue. Synapse 62(4):302–309 10.1002/syn.20496 18241047

[CR9] Caillard O, Ben-Ari Y, Gaïarsa JL (1999) Mechanisms of induction and expression of long-term depression at GABAergic synapses in the neonatal rat hippocampus. J Neurosci 19(17):7568–7577 10.1523/JNEUROSCI.19-17-07568.1999 PMC6782510 10460263

[CR10] Cancedda L, Fiumelli H, Chen K1, Poo MM (2007) Excitatory GABA action is essential for morphological maturation of cortical neurons in vivo. J Neurosci 27(19):5224–5235 10.1523/JNEUROSCI.5169-06.2007 PMC6672363 17494709

[CR11] Chamma I, Chevy Q, Poncer JC, Lévi S (2012) Role of the neuronal K-Cl co-transporter KCC2 in inhibitory and excitatory neurotransmission. Front Cell Neurosci 6:5 10.3389/fncel.2012.00005 PMC3282916 22363264

[CR12] Chamma I, Heubl M, Chevy Q, Renner M, Moutkine I, Eugène E, Poncer JC, Lévi S (2013) Activity-dependent regulation of the K/Cl transporter KCC2 membrane diffusion, clustering, and function in hippocampal neurons. J Neurosci 33(39):15488–15503 10.1523/JNEUROSCI.5889-12.2013 PMC6618451 24068817

[CR13] Chen P, Hu JJ, Liu Y, Cao B, Song XJ (2025) VCP controls KCC2 degradation through FAF1 recruitment and accelerates emergence from anesthesia. Proc Natl Acad Sci USA 122(1):e2414016122 10.1073/pnas.2414016122 PMC11725920 39793039

[CR14] Chevy Q, Heubl M, Goutierre M, Backer S, Moutkine I, Eugene E, Bloch-Gallego E, Levi S, Poncer JC (2015) KCC2 gates activity-driven AMPA receptor traffic through cofilin phosphorylation. J Neurosci 35(48):15772–15786 10.1523/JNEUROSCI.1735-15.2015 PMC6605455 26631461

[CR15] Chevy Q, Simonnet C, Al Awabdh S, Lévi S, Poncer JC (2020) Transport-dependent and independent functions of KCC2 at excitatory synapses. In: Tang X (ed) Neuronal chloride transporters in health and disease. Academic Press, pp 133–158

[CR16] Chi G, Ebenhoch R, Man H, Tang H, Tremblay LE, Reggiano G, Qiu X, Bohstedt T, Liko I, Almeida FG et al (2021) Phospho-regulation, nucleotide binding and ion access control in potassium-chloride cotransporters. EMBO J 40:e107294 10.15252/embj.2020107294 PMC8280820 34031912

[CR17] Côme E, Heubl M, Schwartz EJ, Poncer JC, Lévi S (2019) Reciprocal regulation of KCC2 trafficking and synaptic activity. Front Cell Neurosci 13:48 10.3389/fncel.2019.00048 PMC6391895 30842727

[CR18] Coull JA, Boudreau D, Bachand K, Prescott SA, Nault F, Sik A, De Koninck P, De Koninck Y (2003) Trans-synaptic shift in anion gradient in spinal lamina I neurons as a mechanism of neuropathic pain. Nature 424(6951):938–942 10.1038/nature01868 12931188

[CR19] Delpire E, Gagnon KB (2007) Genome-wide analysis of SPAK/OSR1 binding motifs. Physiol Genomics 28(2):223–231 10.1152/physiolgenomics.00173.2006 17032814

[CR20] Donneger F, Zanin A, Besson J, Roussel D, Kadiri Y, Pagan C, Sinha M, David N, Russeau M, Bielle F et al (2026) Enhancing KCC2 function reduces interictal activity and prevents seizures in temporal lobe epilepsy. Proc Natl Acad Sci USA 123(10):e2522722123 10.1073/pnas.2522722123 PMC12974483 41774803

[CR21] Dubois CJ, Cardoit L, Schwarz V, Markkanen M, Airaksinen MS, Uvarov P, Simmers J, Thoby-Brisson M (2018) Role of the K+-Cl- cotransporter KCC2a isoform in mammalian respiration at birth. eNeuro 5(5):ENEURO.0264-18.2018 10.1523/ENEURO.0264-18.2018 PMC6220586 30406192

[CR22] Friedel P, Kahle KT, Zhang J, Hertz N, Pisella LI, Buhler E, Schaller F, Duan J, Khanna AR, Bishop PN et al (2015) WNK1-regulated inhibitory phosphorylation of the KCC2 cotransporter maintains the depolarizing action of GABA in immature neurons. Sci Signal 8(383):ra65 10.1126/scisignal.aaa0354 26126716

[CR23] Friedel P, Ludwig A, Pellegrino C, Agez M, Jawhari A, Rivera C, Medina I (2017) A novel view on the role of intracellular tails in surface delivery of the potassium-chloride cotransporter KCC2. eNeuro 4(4):ENEURO.0055-17.2017 10.1523/ENEURO.0055-17.2017 PMC5520751 28785725

[CR24] Gauvain G, Chamma I, Chevy Q, Cabezas C, Irinopoulou T, Bodrug N, Carnaud M, Lévi S, Poncer JC (2011) The neuronal K-Cl cotransporter KCC2 influences postsynaptic AMPA receptor content and lateral diffusion in dendritic spines. Proc Natl Acad Sci USA 108(37):15474–15479 10.1073/pnas.1107893108 PMC3174661 21878564

[CR25] Gong Y, Wu M, Gao F, Shi M, Gu H, Gao R, Dang BQ, Chen G (2021) Inhibition of the p‑SPAK/p‑NKCC1 signaling pathway protects the blood‑brain barrier and reduces neuronal apoptosis in a rat model of surgical brain injury. Mol Med Rep 24(4):717 10.3892/mmr.2021.12356 34396440

[CR26] Goutierre M, Al Awabdh S, Donneger F, Francois E, Gomez-Dominguez D, Irinopoulou T, Menendez de la Prida L, Poncer JC (2019) KCC2 regulates neuronal excitability and hippocampal activity via interaction with Task-3 channels. Cell Rep 28:91–103.e107 10.1016/j.celrep.2019.06.001 31269453

[CR27] Gulyas AI, Sik A, Payne JA, Kaila K, Freund TF (2001) The KCl cotransporter, KCC2, is highly expressed in the vicinity of excitatory synapses in the rat hippocampus. Eur J Neurosci 13(12):2205–2217 10.1046/j.0953-816x.2001.01600.x 11454023

[CR28] Hartmann AM, Nothwang HG (2022) NKCC1 and KCC2: structural insights into phospho-regulation. Front Mol Neurosci 15:964488 10.3389/fnmol.2022.964488 PMC9355526 35935337

[CR29] Heubl M, Zhang J, Pressey JC, Al Awabdh S, Renner M, Gomez-Castro F, Moutkine I, Eugène E, Russeau M, Kahle KT et al (2017) GABAA receptor dependent synaptic inhibition rapidly tunes KCC2 activity via the Cl-sensitive WNK1 kinase. Nat Commun 8(1):1776 10.1038/s41467-017-01749-0 PMC5701213 29176664

[CR30] Hinz L, Torrella Barrufet J, Heine VM (2019) KCC2 expression levels are reduced in post mortem brain tissue of Rett syndrome patients. Acta Neuropathol Commun 7(1):196 10.1186/s40478-019-0852-x PMC6892240 31796123

[CR31] Hu JJ, Liu Y, Yao H, Cao B, Liao H, Yang R, Chen P, Song XJ (2023) Emergence of consciousness from anesthesia through ubiquitin degradation of KCC2 in the ventral posteromedial nucleus of the thalamus. Nat Neurosci 26(5):751–764 10.1038/s41593-023-01290-y 36973513

[CR32] Huberfeld G, Wittner L, Clemenceau S, Baulac M, Kaila K, Miles R, Rivera C (2007) Perturbed chloride homeostasis and GABAergic signaling in human temporal lobe epilepsy. J Neurosci 27(37):9866–9873 10.1523/JNEUROSCI.2761-07.2007 PMC6672644 17855601

[CR33] Hübner CA, Stein V, Hermans-Borgmeyer I, Meyer T, Ballanyi K, Jentsch TJ (2001) Disruption of KCC2 reveals an essential role of K-Cl cotransport already in early synaptic inhibition. Neuron 30(2):515–5240 10.1016/s0896-6273(01)00297-5 11395011

[CR34] Inoue K, Yamada J, Ueno S, Fukuda A (2006) Brain-type creatine kinase activates neuron-specific K+-Cl- co-transporter KCC2. J Neurochem 96(2):598–608 10.1111/j.1471-4159.2005.03560.x 16336223

[CR35] Inoue K, Furukawa T, Kumada T, Yamada J, Wang T, Inoue R, Fukuda A (2012) Taurine inhibits K+-Cl- cotransporter KCC2 to regulate embryonic Cl- homeostasis via with-no-lysine (WNK) protein kinase signaling pathway. JBC 287(25):20839–20850 10.1074/jbc.M111.319418 PMC3375508 22544747

[CR36] Ivakine EA, Acton BA, Mahadevan V, Ormond J, Tang M, Pressey JC, Huang MY, Ng D, Delpire E, Salter MW et al (2013) Neto2 is a KCC2 interacting protein required for neuronal Cl- regulation in hippocampal neurons. Proc Natl Acad Sci USA 110(9):3561–3566 10.1073/pnas.1212907110 PMC3587235 23401525

[CR37] Kahle KT, Deeb TZ, Puskarjov M, Silayeva L, Liang B, Kaila K, Moss SJ (2013) Modulation of neuronal activity by phosphorylation of the K-Cl cotransporter KCC2. Trends Neurosci 36(12):726–737 10.1016/j.tins.2013.08.006 PMC4381966 24139641

[CR38] Kaila K, Price TJ, Payne JA, Puskarjov M, Voipio J (2014) Cation-chloride cotransporters in neuronal development, plasticity and disease. Nat Rev Neurosci 15(10):637–654 10.1038/nrn3819 PMC4294553 25234263

[CR39] Keramidis I, McAllister BB, Bourbonnais J, Wang F, Isabel D, Rezaei E, Sansonetti R, Degagne P, Hamel JP, Nazari M et al (2023) Restoring neuronal chloride extrusion reverses cognitive decline linked to Alzheimer’s disease mutations. Brain 146(12):4903–4915 10.1093/brain/awad250 PMC10690023 37551444

[CR40] Khirug S, Yamada J, Afzalov R, Voipio J, Khiroug L, Kaila K (2008) GABAergic depolarization of the axon initial segment in cortical principal neurons is caused by the Na-K-2Cl cotransporter NKCC1. J Neurosci 28(18):4635–4639 10.1523/JNEUROSCI.0908-08.2008 PMC6670448 18448640

[CR41] Kittler JT, Delmas P, Jovanovic JN, Brown DA, Smart TG, Moss SJ (2000) Constitutive endocytosis of GABAA receptors by an association with the adaptin AP2 complex modulates inhibitory synaptic currents in hippocampal neurons. J Neurosci 20(21):7972–7977 10.1523/JNEUROSCI.20-21-07972.2000 PMC6772725 11050117

[CR42] Lee HH, Jurd R, Moss SJ (2010) Tyrosine phosphorylation regulates the membrane trafficking of the potassium chloride co-transporter KCC2. Mol Cell Neurosci 45(2):173–179 10.1016/j.mcn.2010.06.008 PMC3529177 20600929

[CR43] Li H, Khirug S, Cai C, Ludwig A, Blaesse P, Kolikova J, Afzalov R, Coleman SK, Lauri S, Airaksinen MS et al (2007) KCC2 interacts with the dendritic cytoskeleton to promote spine development. Neuron 56(6):1019–1033 10.1016/j.neuron.2007.10.039 18093524

[CR44] Llano O, Smirnov S, Soni S, Golubtsov A, Guillemin I, Hotulainen P, Medina I, Nothwang HG, Rivera C, Ludwig A (2015) KCC2 regulates actin dynamics in dendritic spines via interaction with beta-PIX. JCB 209(5):671–686 10.1083/jcb.201411008 PMC4460141 26056138

[CR45] Mahadevan V, Khademullah CS, Dargaei Z, Chevrier J, Uvarov P, Kwan J, Bagshaw RD, Pawson T, Emili A, De Koninck Y et al (2017) Native KCC2 interactome reveals PACSIN1 as a critical regulator of synaptic inhibition. eLife 6:e28270 10.7554/eLife.28270 PMC5640428 29028184

[CR46] Markkanen M, Karhunen T, Llano O, Ludwig A, Rivera C, Uvarov P, Airaksinen MS (2014) Distribution of neuronal KCC2a and KCC2b isoforms in mouse CNS. J Comp Neurol 522(8):1897–1914 10.1002/cne.23510 24639001

[CR47] Markkanen M, Ludwig A, Khirug S, Pryazhnikov E, Soni S, Khiroug L, Delpire E, Rivera C, Airaksinen MS, Uvarov P (2017) Implications of the N-terminal heterogeneity for the neuronal K-Cl cotransporter KCC2 function. Brain Res 1675:87–101 10.1016/j.brainres.2017.08.034 28888841

[CR48] Mavrovic M, Uvarov P, Delpire E, Vutskits L, Kaila K, Puskarjov M (2020) Loss of non-canonical KCC2 functions promotes developmental apoptosis of cortical projection neurons. EMBO Rep 21(4):e48880 10.15252/embr.201948880 PMC7132201 32064760

[CR49] Piechotta K, Lu J, Delpire E (2002) Cation chloride cotransporters interact with the stress-related kinases Ste20-related proline-alanine-rich kinase (SPAK) and oxidative stress response 1 (OSR1). JBC 277(52):50812–50819 10.1074/jbc.M208108200 12386165

[CR50] Pressey JC, de Saint-Rome M, Raveendran VA, Woodin MA (2023) Chloride transporters controlling neuronal excitability. Physiol Rev 103(2):1095–1135 10.1152/physrev.00025.2021 36302178

[CR51] Puskarjov M, Ahmad F, Kaila K, Blaesse P (2012) Activity-dependent cleavage of the K-Cl cotransporter KCC2 mediated by calcium-activated protease calpain. J Neurosci 32(33):11356–11364 10.1523/JNEUROSCI.6265-11.2012 PMC6621186 22895718

[CR52] Raimondo JV, Markram H, Akerman CJ (2012) Short-term ionic plasticity at GABAergic synapses. Front Synaptic Neurosci 4:5 10.3389/fnsyn.2012.00005 PMC3472547 23087642

[CR53] Rivera C, Voipio J, Payne JA, Ruusuvuori E, Lahtinen H, Lamsa K, Pirvola U, Saarma M, Kaila K (1999) The K+/Cl- co-transporter KCC2 renders GABA hyperpolarizing during neuronal maturation. Nature 397(6716):251–255 10.1038/16697 9930699

[CR54] Rivera C, Voipio J, Thomas-Crusells J, Li H, Emri Z, Sipila S, Payne JA, Minichiello L, Saarma M, Kaila K (2004) Mechanism of activity-dependent downregulation of the neuron-specific K-Cl cotransporter KCC2. J Neurosci 24(19):4683–4691 10.1523/JNEUROSCI.5265-03.2004 PMC6729393 15140939

[CR55] Simonnet C, Sinha M, Goutierre M, Moutkine I, Daumas S, Poncer JC (2023) Silencing KCC2 in mouse dorsal hippocampus compromises spatial and contextual memory. Neuropsychopharmacology 48(7):1067–1077 10.1038/s41386-022-01480-5 PMC10209115 36302847

[CR56] Smalley JL, Kontou G, Choi C, Ren Q, Albrecht D, Abiraman K, Santos MAR, Bope CE, Deeb TZ, Davies PA et al (2020) Isolation and Characterization of multi-protein complexes enriched in the K-Cl co-transporter 2 from brain plasma membranes. Front Mol Neurosci 13: 563091 10.3389/fnmol.2020.563091 PMC7643010 33192291

[CR57] Uvarov P, Ludwig A, Markkanen M, Pruunsild P, Kaila K, Delpire E, Timmusk T, Rivera C, Airaksinen MS (2007) A novel N-terminal isoform of the neuron-specific K-Cl cotransporter KCC2. JBC 282(42):30570–30576 10.1074/jbc.M705095200 17715129

[CR58] Uvarov P, Ludwig A, Markkanen M, Soni S, Hübner CA, Rivera C, Airaksinen MS (2009) Coexpression and heteromerization of two neuronal K-Cl cotransporter isoforms in neonatal brain. JBC 284(20):13696–13704 10.1074/jbc.M807366200 PMC2679471 19307176

[CR59] Virtanen MA, Uvarov P, Mavrovic M, Poncer JC, Kaila K (2021) The multifaceted roles of KCC2 in cortical development. Trends Neurosci 44(5):378–392 10.1016/j.tins.2021.01.004 33640193

[CR60] Wang DD, Kriegstein AR (2008) GABA regulates excitatory synapse formation in the neocortex via NMDA receptor activation. J Neurosci 28(21):5547–5558 10.1523/JNEUROSCI.5599-07.2008 PMC2684685 18495889

[CR61] Watanabe M, Wake H, Moorhouse AJ, Nabekura J (2009) Clustering of neuronal K+-Cl- cotransporters in lipid rafts by tyrosine phosphorylation. JBC 284(41):27980–27988 10.1074/jbc.M109.043620 PMC2788850 19679663

[CR62] Woo NS, Lu J, England R, McClellan R, Dufour S, Mount DB, Deutch AY, Lovinger DM, Delpire E (2002) Hyperexcitability and epilepsy associated with disruption of the mouse neuronal-specific K-Cl cotransporter gene. Hippocampus 12(2):258–268 10.1002/hipo.10014 12000122

[CR63] Zhao B, Wong AY, Murshid A, Bowie D, Presley JF, Bedford FK (2008) Identification of a novel di-leucine motif mediating K(+)/Cl(-) cotransporter KCC2 constitutive endocytosis. Cell Signal 20(10):1769–1779 10.1016/j.cellsig.2008.06.011 18625303

