# Supplementary material for: Differential isoform-specific control of KCC2 function in developing and mature cortical neurons
Source: EMBO Rep. 2026 Sep 5;27(20):5837–59. doi: 10.1038/s44319-026-00920-3 (PMC13694278; doi:10.1038/s44319-026-00920-3)
Supplement: Supplementary file 1 [file 44319_2026_920_MOESM4_ESM.pdf]

EMBO reports

Differential isoform-specific control of KCC2 function in developing and mature cortical neurons

Carla Pagan, Pauline Weinzettl, Marika Markkanen, Marion Russeau, Matti Airaksinen, and Jean Christophe Poncer

Corresponding author(s): Jean Christophe Poncer (jean-christophe.poncer@inserm.fr)

**Review Timeline:**		
	Submission Date:	22nd Jan 26
	Editorial Decision:	11th Mar 26
	Revision Received:	27th Jun 26
	Editorial Decision:	6th Aug 26
	Revision Received:	19th Aug 26
	Accepted:	24th Aug 26

Editor: Esther Schnapp

Transaction Report:

(Note: With the exception of the correction of typographical or spelling errors that could be a source of ambiguity, letters and reports are not edited. Depending on transfer agreements, referee reports obtained elsewhere may or may not be included in this compilation. Referee reports are anonymous unless the Referee chooses to sign their reports.)

Dear Dr. Poncer,

Thank you for the submission of your manuscript to EMBO reports. We have now received the full set of referee reports that is pasted below.

As you will see, the referees acknowledge that the findings are interesting. However, they also have several suggestions for how the study could be improved and I think all suggestions are good and should be addressed, but please let me know in case you disagree, and we can discuss the exact revision requirements further, also in a video chat, if you like.

Before sending your ms for in-depth review I contacted an advisor and also paste some of her/his comments here :

- Most mechanistic claims are culture-based, and the only direct in vivo data are biochemical (western blots at P9 and adult). There is no in vivo functional readout (e.g., EGABA / ECl, inhibitory synaptic efficacy, network excitability) in KO animals in this manuscript.

- The "stabilization" mechanism in mature neurons relies heavily on overexpressed KCC2b-Flag surface assays plus pharmacological perturbations. These are standard approaches, but reviewers should evaluate specificity and whether conclusions extend to endogenous KCC2b trafficking/degradation.

It would be great if these points could also be further strengthened experimentally.

I would thus like to invite you to revise your manuscript with the understanding that the referee concerns must be fully addressed and their suggestions taken on board. Please address all referee concerns in a complete point-by-point response. Acceptance of the manuscript will depend on a positive outcome of a second round of review. It is EMBO reports policy to allow a single round of major revision only and acceptance or rejection of the manuscript will therefore depend on the completeness of your responses included in the next, final version of the manuscript.

We realize that it is difficult to revise to a specific deadline. In the interest of protecting the conceptual advance provided by the work, we recommend a revision within 3 months (11th Jun 2026). Please discuss the revision progress ahead of this time with the editor if you require more time to complete the revisions.

IMPORTANT NOTE: we perform an initial quality control of all revised manuscripts before re-review. Your manuscript will FAIL this control and the handling will be DELAYED if the following APPLIES:
1) A data availability section providing access to data deposited in public databases is missing. If you have not deposited any data, please add a sentence to the data availability section that explains that.
2) Your manuscript contains statistics and error bars based on n=2. Please use scatter blots in these cases. No statistics should be calculated if n=2.

When submitting your revised manuscript, please review the submission guidelines (https://link.springer.com/journal/44319/submission-guidelines#cms-Revised-submissions) and carefully review the instructions that follow below. Failure to include requested items will delay the evaluation of your revision.

1) a .docx formatted version of the manuscript text (including legends for main figures, EV figures and tables). Please make sure that the changes are highlighted to be clearly visible.

2) individual production quality figure files as .eps, .tif, .jpg (one file per figure). See hhttps://media.springernature.com/original/springer-cms/rest/v1/content/27825798/data/v1 for more info on how to prepare your figures.

3) We replaced Supplementary Information with Expanded View (EV) Figures and Tables that are collapsible/expandable online. A maximum of 5 EV Figures can be typeset. EV Figures should be cited as 'Figure EV1, Figure EV2" etc... in the text and their respective legends should be included in the main text after the legends of regular figures.

- For the figures that you do NOT wish to display as Expanded View figures, they should be bundled together with their legends in a single PDF file called *Appendix*, which should start with a short Table of Content. Appendix figures should be referred to in the main text as: "Appendix Figure S1, Appendix Figure S2" etc. See 'Expanded View' section of the submission guidelines.

- Additional Tables/Datasets should be labeled and referred to as Table EV1, Dataset EV1, etc. Legends have to be provided in a separate tab in case of .xls files. Alternatively, the legend can be supplied as a separate text file (README) and zipped together with the Table/Dataset file.

4) a .docx formatted letter INCLUDING the reviewers' reports and your detailed point-by-point responses to their comments. As

part of the EMBO Press transparent editorial process, the point-by-point response is part of the Review Process File (RPF), which will be published alongside your paper.

5) a complete author checklist, which you can download from our author guidelines. Please insert information in the checklist that is also reflected in the manuscript. The completed author checklist will also be part of the RPF.

6) Please note that all corresponding authors are required to supply an ORCID ID for their name upon submission of a revised manuscript (<https://orcid.org/>), which can be linked to your profile in the manuscript submission system.

7) Before submitting your revision, primary datasets produced in this study need to be deposited in an appropriate public database. Please remember to provide a reviewer password if the datasets are not yet public. The accession numbers and database should be listed in a formal "Data Availability" section placed after Methods. Please note that the Data Availability Section is restricted to new primary data that are part of this study. * Note - All links should resolve to a page where the data can be accessed. *

If your study has not produced novel datasets, please mention this fact in the Data Availability Section.

8) At EMBO Press we ask authors to provide source data for the main manuscript figures. You will receive a separate email with instructions for providing source data with your revised manuscript, including how to upload and organize the files.

9) Our journal also encourages inclusion of *data citations in the reference list* to directly cite datasets that were re-used and obtained from public databases. Data citations in the article text are distinct from normal bibliographical citations and should directly link to the database records from which the data can be accessed. In the main text, data citations are formatted as follows: "Data ref: Smith et al, 2001" or "Data ref: NCBI Sequence Read Archive PRJNA342805, 2017". In the Reference list, data citations must be labeled with "[DATASET]". A data reference must provide the database name, accession number/identifiers and a resolvable link to the landing page from which the data can be accessed at the end of the reference.

10) Regarding data quantification, the following points must be specified in each figure legend:

- the name of the statistical test used to generate error bars and P values,

- the number (n) of independent experiments (please specify technical or biological replicates) underlying each data point,

- the nature of the bars and error bars (s.d., s.e.m.),

- If the data are obtained from n Program fragment delivered error ``Can't locate object method "less" via package "than" (perhaps you forgot to load "than"?) at //ejpvfs23/sites23b/embor_www/letters/embor_decision_revise_and_review.txt line 54.' 2, use scatter blots showing the individual data points.

Discussion of statistical methodology can be reported in the materials and methods section, but figure legends should contain a basic description of n, P and the test applied.

- Please also include scale bars in all microscopy images.

11) The journal requires a statement specifying whether or not authors have competing interests (defined as all potential or actual interests that could be perceived to influence the presentation or interpretation of an article). In case of competing interests, this must be specified in your disclosure statement.

12) All Materials and Methods need to be described in the main text using our 'Structured Methods' format, which is required for all research articles. According to this format, the Methods section includes a separate Reagents and Tools Table file (listing key reagents, experimental models, software and relevant equipment and including their sources and relevant identifiers) and a Methods and Protocols section describing the methods using a step-by-step protocol format. The aim is to facilitate adoption of the methodologies across labs. More information on how to adhere to this format as well as a downloadable template (.docx) for the Reagents and Tools Table can be found in our author guidelines.

We would also welcome the submission of cover suggestions, or motifs to be used by our Graphics Illustrator in designing a cover.

As part of the EMBO publication's Transparent Editorial Process, EMBO reports publishes online a Review Process File (RPF) to accompany accepted manuscripts. This File will be published in conjunction with your paper and will include the referee reports, your point-by-point response and all pertinent correspondence relating to the manuscript.

You are able to opt out of this by letting the editorial office know (emboreports@embo.org). If you do opt out, the Review Process File link will point to the following statement: "No Review Process File is available with this article, as the authors have

chosen not to make the review process public in this case."

I look forward to seeing a revised form of your manuscript when it is ready.

Best regards,
Esther

Esther Schnapp, PhD
Senior Editor
EMBO reports

Referee #1:

This manuscript by Pagan et al. reports an interesting and potentially important discovery that KCC2a, the minor splice isoform of KCC2, regulates the phosphorylation, membrane stability, and functional expression of the major isoform, KCC2b. The authors propose that KCC2a prevents clathrin-mediated endocytosis, polyubiquitination, and proteasomal degradation of KCC2b, thereby stabilizing KCC2b at the plasma membrane in mature neurons. This study introduces KCC2a as a context-dependent regulator of KCC2b and suggests a developmental switch in which KCC2a initially suppresses KCC2b membrane expression but later promotes its stabilization.

The concept of a developmental switch is intriguing, particularly given that KCC2a transcripts are relatively abundant at embryonic stages. However, this raises several conceptual questions. In early development, KCC2b expression is already low-why would KCC2a be required to further suppress it? What molecular mechanism drives the transition from inhibition to stabilization? The data implicating changes in KCC2 phosphorylation and SPAK signaling in KCC2a knockout mice at P9 are striking and somewhat unexpected. This raises the possibility that KCC2a may act as an organizer or scaffold for WNK-SPAK signaling. However, these interactions are likely transient, and additional validation, such as co-IP, would strengthen the mechanistic claims.

Several technical and interpretational issues require clarification.

In Figure 2, does the KCC2a shRNA knock down endogenous mouse Kcc2a? The authors should explicitly demonstrate isoform specificity using the KCC2a antibody that appears validated in Figure 1 to detect the endogenous KCC2a protein, instead of relying on immunostaining of overexpressed FLAG-KCC2a.

In Figure 4D, panel placement may be incorrect, and the large difference in peak amplitude between shNT and shKCC2a groups raises the possibility that KCC2a may regulate GABA_A receptor functional expression in addition to chloride extrusion. The authors should address this concern through experiments.

The finding that KCC2a is absent from dendritic spines, whereas KCC2b is spine-enriched, is particularly intriguing. This raises the possibility that the N-terminus of KCC2b contains spine-localization determinants absent in KCC2a. Functional consequences of KCC2a deletion on excitatory/inhibitory balance, including dendritic spine analysis, mEPSC frequency and amplitude, EGABA measurements should be examined both in acute knockdown neurons and in KCC2a knockout mice to better define circuit-level consequences.

Finally, the authors speculate that KCC2a knockout or disruption of KCC2a-SPAK interaction could be an anti-epileptic strategy. However, this interpretation appears inconsistent with their own data showing that KCC2a knockdown in mature neurons reduces KCC2b membrane expression. This point requires clarification, as it has important therapeutic implications.

The authors are strongly recommended to provide a schematic diagram summarizing the proposed model, which would greatly enhance clarity.

Referee #2:

This is an excellent study. The authors address a long-standing, important question in the field-the functional role of the minor KCC2a isoform. They did a series of sophisticated in vitro and in vivo experiments to show that KCC2a controls the main KCC2b type in both directions and depending on the situation. The most important finding is that KCC2a switches from an inhibitory to a stabilizing role depending on WNK-SPAK pathway activity. This is new and basically changes the way KCC2 is regulated during neuronal development. The manuscript is well-organized, the data are convincing, and the conclusions are mostly well supported.

The main results of the manuscript are already strong enough. However, it would make an already excellent study truly definitive by including the following experiments. I also think that the authors may mention it in the Discussion section as future work, instead of adding data to this manuscript.
1. The conclusions are supported by a strong, multi-part experimental approach using isoform-specific knockdown, knockout mice, pharmacological inhibitors, and functional assays. The evidence shows clearly what happened. To further prove the mechanistic claims and increase the impact, the following experiments are suggested: to perform a rescue experiment with a phospho-dead KCC2b mutant (T1007A). The model shows that in KCC2a-deficient mature neurons where WNK1-CA is overexpressed, the membrane expression of wild-type KCC2b should decrease, but the KCC2b-T1007A mutant should be resistant to this downregulation. However, this mutant might show altered baseline stability. This experiment would establish a direct causal link between Thr1007 phosphorylation and the observed membrane destabilization, moving beyond correlation.
2. Try proximity ligation assays (PLA) or co-IP from neuronal lysates to show the KCC2a-SPAK-KCC2b interaction complex in the cell, and how it changes with development or WNK-SPAK activation.

Minors:
1. Page 9, Results. "Under basal conditions..., specifically blocking the proteasome... or polyubiquitination... for two hours did not significantly reduce endogenous KCC2 expression... These results suggest that KCC2a controls KCC2 protein levels by preventing its polyubiquitination and subsequent degradation by the proteasome."
---This sentence could be nicely modified as follows: "These results suggest that KCC2a protects KCC2b from polyubiquitination and subsequent degradation by the proteasome, a degradation pathway that becomes engaged upon KCC2a loss."

2. Page 21, Material and Methods: "...the proteasome inhibitor MG132 (2.5 μM...). As MG132 is a known calpain inhibitor (IC50 = 1.2 μM), this treatment condition also served to inhibit calpain-dependent degradation of KCC2".
---Thus, in Page 9, after Fig 5E, It might be helpful to add some words like: "The effect of MG132, which also inhibits calpain at this concentration, suggests the proteasomal pathway is primarily involved, though a contribution from calpain inhibition cannot be fully excluded."

Referee #3:

The K/Cl cotransporter KCC2 regulates chloride balance in neurons, thus playing fundamental roles in neuronal physiology in both normal and pathological conditions. In particular, reduced KCC2 activity has been linked to neurological disorders, including epilepsy chronic pain and traumatic injury. A better understanding of the mechanisms regulating its endogenous activity could help designing novel pharmacological approaches to regulate KCC2 function and chloride balance in pathological conditions.

KCC2 comes in two flavors, KCC2a and KCC2b, the latter being the dominant isoform. In this study, Pagan et al investigate the role of KCC2a in regulating KCC2a expression and function. They convincingly demonstrated that :

1) In mature hippocampal neurons, suppressing KCC2a significantly reduces total KCC2 protein levels and impairs dendritic chloride extrusion. They further showed that KCC2a promotes KCC2b clustering and prevents its clathrin-mediated endocytosis and subsequent proteasomal degradation.

2) In immature neurons, KCC2a contributes to maintaining low KCC2b membrane expression, by promoting KCC2b Thr1007 phosphorylation. This likely occurs because WNK-SPAK pathway is more active in immature than mature neurons.

The study is timely, the conclusions are supported by the data, and the manuscripts is well written.

I have a few comments and questions that should be addressed by explanation in the text or revising the chosen images:

1) In legend 1: "specific mRNA expression relative to Ywhaz ", and methods: which protein does the housekeeping gene Ywhaz code for? Why was this specific gene chosen as housekeeping gene?

2) Figure 5B. The dendrite in DiP-treated culture appear to bear more spine, why? Is it due to how the images were chosen? Is there any effects on spine density?

3) Relative to the same figure: Why is GFP signal stronger in DiP treated dendrites?

4)Please, add the catalog number of the reagents (in particular for commercial antibodies) used in this study.

5) In general, the resolution of the images showing dendrites (for example panel 1G, panel 2C,E, 3B, 3E etc) is quite low. Maybe this is due to the PDF conversion, but it would have been more informative to be able to look at higher resolution images.

We thank the editor and the three referees for their thorough and constructive evaluation of our manuscript. We are grateful for the positive assessment of the study's significance and rigor. We have carried out additional experiments and analyses, revised the text, and added a schematic of the proposed model. New data and text additions are highlighted in yellow in the revised manuscript. Below, each comment is reproduced in italics and followed by our response; wherever a change was made to the main document, we quote the corresponding sentence and give its page and line number.

Advisor's comments

- Most mechanistic claims are culture-based, and the only direct in vivo data are biochemical (western blots at P9 and adult). There is no in vivo functional readout (e.g., EGABA / ECl, inhibitory synaptic efficacy, network excitability) in KO animals in this manuscript.

We agree. New functional and behavioral cohorts could not be generated during this revision because our KCC2a-KO colony is currently being re-established and no experimental animals of the required ages were available. We have therefore framed these experiments as a priority for future work. In the meantime, the in vivo mechanistic conclusions of the study rest on biochemical measurements in KCC2a-KO brain that are already part of the manuscript, namely the loss of KCC2b Thr1007 phosphorylation and the reduction of SPAK Ser373 phosphorylation at P9 (Fig. 6).

- The "stabilization" mechanism in mature neurons relies heavily on overexpressed KCC2b-Flag surface assays plus pharmacological perturbations. These are standard approaches, but reviewers should evaluate specificity and whether conclusions extend to endogenous KCC2b trafficking/degradation.

Two considerations motivated the use of recombinant Flag-tagged constructs. First, the 3xFlag epitope inserted into the second extracellular loop is what allows live labelling of the plasma-membrane pool and hence the surface-to-total measurement in intact neurons; antibodies directed against native KCC2b recognize intracellular epitopes and do not permit live surface labelling. Second, our conclusions on protein level and degradation do not rest on the recombinant assays alone: the reduction of total KCC2 upon KCC2a knockdown, and its rescue by proteasome or ubiquitination blockade, were both established on endogenous KCC2 detected with a pan-KCC2 antibody (Fig. 3A–C and Fig. 5D–E). The recombinant constructs were used specifically to resolve the membrane-trafficking component, which is not otherwise accessible. We did not re-run the surface assay on endogenous KCC2b for the reasons above.

Referee #1

1.1a. The concept of a developmental switch is intriguing, particularly given that KCC2a transcripts are relatively abundant at embryonic stages. However, this raises several conceptual questions. In early development, KCC2b expression is already low-why would KCC2a be required to further suppress it?

This is a valid, albeit conceptual, question that is difficult to address experimentally. Several mechanisms converge to control KCC2 function during cortical development. These mechanisms include transcriptional regulation, particularly of the KCC2b isoform, as well as posttranslational mechanisms, such as the WNK-SPAK pathway. Though these mechanisms may appear redundant, they likely serve distinct, age-dependent functions. The transport-independent functions of KCC2, which are mediated by its C-terminal domain and include the regulation of actin/cofilin dynamics, are essential during corticogenesis. Their loss promotes the developmental apoptosis of cortical projection neurons

(Mavrovic et al., 2020). Meanwhile, maintaining a depolarizing effect of GABA on the membrane potential of immature neurons, which is ensured by a lack of KCC2 transport function, is important for several aspects of neurodevelopment, including synaptogenesis and establishing proper neuronal connections (Caillard et al., 1999; Cancedda et al., 2007; Wang et al., 2008). Thus, the coexistence of apparently redundant regulatory mechanisms may reflect the need to differentially control canonical (ion transport) and noncanonical (structural) KCC2 functions, which rely on different combinations of membrane stability and protein expression. Consistent with this view, we found that KCC2a KO reduced threonine 1007 phosphorylation at P9 without significantly reducing total KCC2 expression. We discuss this further in the Discussion section on page 17.

1.1b. What molecular mechanism drives the transition from inhibition to stabilization?

In immature neurons, membrane KCC2b is low but not null, and is actively held down: by promoting Thr1007 phosphorylation of KCC2b, KCC2a provides an active brake that keeps surface KCC2b and chloride extrusion immature even as total KCC2 protein rises. Crucially, the transition from inhibition to stabilization does not require any change in the molecular action of KCC2a. The same KCC2a–KCC2b coupling has opposite consequences depending on WNK-SPAK pathway activity: when WNK-SPAK activity is high (immature stages), the coupling enables SPAK-mediated KCC2b Thr1007 phosphorylation and destabilization; when WNK-SPAK activity declines (mature stages), the same physical association instead protects KCC2b from clathrin-mediated endocytosis and proteasomal degradation. The developmental fall in WNK-SPAK activity is thus sufficient to convert KCC2a from a negative into a positive regulator. This logic is the backbone of the model laid out in the Discussion and summarized in the new schematic (Graphical Abstract).

1.2. The data implicating changes in KCC2 phosphorylation and SPAK signaling in KCC2a knockout mice at P9 are striking and somewhat unexpected. This raises the possibility that KCC2a may act as an organizer or scaffold for WNK-SPAK signaling. However, these interactions are likely transient, and additional validation, such as co-IP, would strengthen the mechanistic claims.

We agree, and proposed exactly such a complex in the original manuscript. Specific co-immunoprecipitation of SPAK with the KCC2a isoform (but not KCC2b) has already been demonstrated in heterologous cells (Markkanen et al., 2017). To extend this to native tissue, we attempted to co-immunoprecipitate SPAK and KCC2 isoforms from P9 and adult brain lysates of WT and KCC2a-KO mice, using either anti-SPAK or anti-KCC2b antibodies for immunoprecipitation. We encountered two major issues with this experiment. i) Although our isoform-specific antibodies perform well in immunocytochemistry and in western blot on heterologous cells, they yielded prohibitively high background on the pre-clear and unbound fraction of our whole-brain extracts obtained during our co-IP experiment, which prevented us from detecting endogenous KCC2a and KCC2b proteins before and after the immunoprecipitation. Yet, this is an important control in this experiment. ii) We obtained an enrichment of the SPAK signal after its immunoprecipitation but we did not detect any KCC2a or KCC2b signal in the IP fraction.

It seems that co-immunoprecipitating KCC2 and SPAK from brain extracts is particularly challenging. Several studies that explored the KCC2 interactome did not succeed in precipitating SPAK together with KCC2 (Mahadevan et al, 2017; Al Awabdh et al, 2022). Together with Dr P. Uvarov, who generated these isoform-specific antibodies, we identified several non-trivial reasons why endogenous co-IP of this

complex from brain is inherently difficult. i) Heterologous systems co-express KCC2 and SPAK/OSR1 at high levels in the same cells, whereas in brain SPAK (Stk39) is expressed at low levels in neurons and is comparatively abundant in non-neuronal compartments such as the choroid plexus (single-cell transcriptomic data, e.g. DropViz); a SPAK pull-down from whole brain therefore recovers a large amount of SPAK from cells that do not express KCC2. ii) SPAK engages a very large interactome — Delpire & Gagnon (2007) identified more than 130 proteins bearing SPAK/OSR1-binding motifs, and subsequent analyses suggest substantially more — so that KCC2 represents only a small fraction of SPAK-bound proteins. iii) The N-terminal KCC2a epitope used to raise the antibody (≈ residues 20–40) contains a PKA phosphorylation site (Ser27) that is phosphorylated in brain according to unbiased phosphoproteomic datasets; because PKA-dependent regulation differs between neurons and HEK293 cells, phosphorylation at this site may occlude antibody recognition in IP from brain, whereas the KCC2b-specific N-terminal epitope contains no comparable site. A further practical constraint is that the chicken anti-KCC2b (IgY) antibody is not captured by Protein A/G and requires anti-IgY agarose or direct NHS coupling for efficient IP.

Importantly, our central conclusion does not depend on directly resolving this complex. The feature that distinguishes the two isoforms is the SPAK/OSR1-binding motif (RFxV/I) present in the KCC2a N-terminus but absent from KCC2b. Consistent with this motif being required for SPAK/OSR1-dependent phospho-regulation, a human N-terminally truncated KCC1 lacking the equivalent OSR1-binding motif (KCC1Δ19) is not phosphorylated at its canonical regulatory sites (Chi et al., 2021). Our data in KCC2a-KO brain are fully congruent with this framework: loss of KCC2a strongly reduces Thr1007 phosphorylation of the remaining KCC2b pool (Fig. 6), exactly as predicted if KCC2a provides the N-terminal SPAK-docking site required for KCC2b phospho-regulation. We have added this point, together with the rationale above for the difficulty of isolating the endogenous complex, to the Discussion (p. 17-18, lines 407–422: "*The mechanistic basis for this model is the N-terminal SPAK/OSR1-binding motif itself … an N-terminally truncated human KCC1 lacking this motif (KCC1Δ19) is no longer phosphorylated at its canonical regulatory threonines (Chi et al, 2021)… direct biochemical isolation of the endogenous KCC2a-SPAK-KCC2b complex from native tissue remains challenging, as neuronal SPAK is expressed at low levels and engages a very large interactome (Delpire & Gagnon, 2007), of which KCC2 represents only a minor fraction*."

1.3. In Figure 2, does the KCC2a shRNA knock down endogenous mouse Kcc2a? The authors should explicitly demonstrate isoform specificity using the KCC2a antibody that appears validated in Figure 1 to detect the endogenous KCC2a protein, instead of relying on immunostaining of overexpressed FLAG-KCC2a.

We have performed this validation. Using the pre-adsorbed anti-KCC2a antibody validated in Figure 1, we measured endogenous KCC2a in neurons expressing the two shRNAs and found a significant 25% reduction ($p<0.001$). Changes in mean KCC2a immunofluorescence across the five cultures were: −38%, +17%, −13%, −41%, and −76%, likely reflecting detection variability near the sensitivity floor of the immunostaining assay for this low-abundance isoform, or genuine culture-to-culture differences in endogenous KCC2a expression. Either way, the unambiguous >60% knockdown of recombinant KCC2a-Flag (Fig. 2E-F) demonstrates that the shRNAs are effective. These new data are shown in a new panel of Fig EV2, with the corresponding immunostaining protocol added to the Methods. (Results, p. 8; Fig

EV2; Methods (immunostaining): "*…reduced recombinant KCC2a expression by over 60% (Fig. 2E-F) and endogenous KCC2a levels by 25% (p<0.001; Fig EV2).*")

1.4. In Figure 4D, panel placement may be incorrect, and the large difference in peak amplitude between shNT and shKCC2a groups raises the possibility that KCC2a may regulate GABA_A receptor functional expression in addition to chloride extrusion. The authors should address this concern through experiments.

We have checked and corrected the panel order in Fig. 4. Regarding amplitude, we would like to clarify why it does not reflect GABAA-receptor expression. In our paradigm, GABAA-receptor currents are evoked by local isoguvacine puffs onto the soma or a dendrite. The absolute amplitude of such puff-evoked currents is intrinsically variable and is not an interpretable index of receptor expression, because it depends on the puff-pipette position and on the membrane area exposed to agonist, neither of which is standardized across cells. Critically, our chloride-extrusion readout (ΔE_{GABA}) is a reversal-potential measurement and is therefore independent of current amplitude. To exclude any systematic bias between groups, we nonetheless compared peak amplitudes at the soma and dendrite between shNT and shKCC2a neurons and found no significant difference (two-way ANOVA across voltage steps: p=0.90 soma, p=0.99 dendrite; t-test at −60 mV: p=0.94 soma, p=0.23 dendrite, **Fig. R1** below). Because absolute puff-evoked amplitudes are not a meaningful measure of receptor expression, we prefer not to add them to the manuscript, where they could be over-interpreted, but would gladly include them as a supplementary panel if the referee considers it useful.

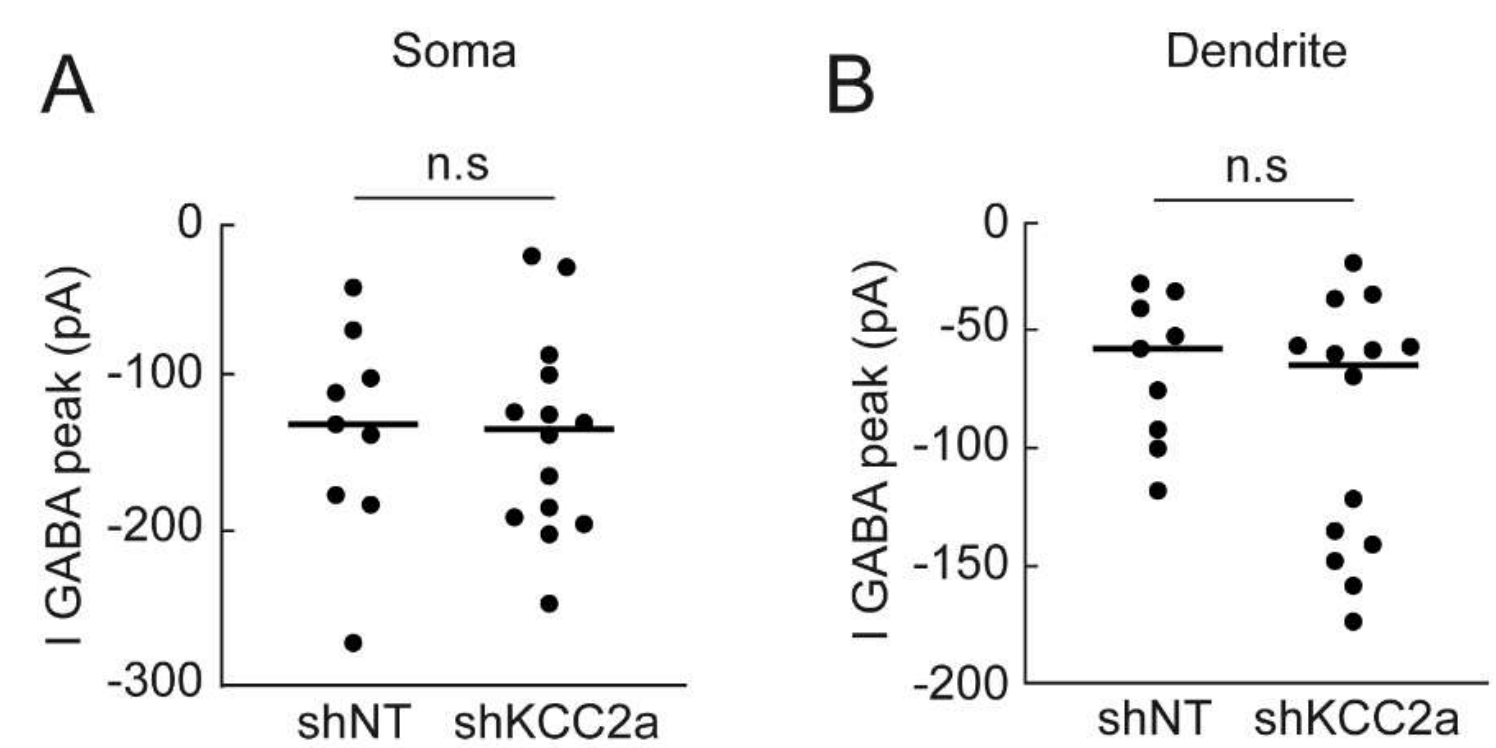

Figure R1: No effect of KCC2a knockdown on the amplitude of evoked GABAAR-mediated currents. Peak amplitude of isoguvacine-evoked currents at the soma (A) and at dendritic sites (B) for all neurons in Figure 4, measured at a hyperpolarized voltage step of −60 mV. No significant difference was found between shNT and shKCC2a neurons (two-tailed t-test, p = 0.937 for the soma and p = 0.229 for the dendrite).

1.5. The finding that KCC2a is absent from dendritic spines, whereas KCC2b is spine-enriched, is particularly intriguing. This raises the possibility that the N-terminus of KCC2b contains spine-localization determinants absent in KCC2a. Functional consequences of KCC2a deletion on excitatory/inhibitory balance, including dendritic spine analysis, mEPSC frequency and amplitude, EGABA measurements should be examined both in acute knockdown neurons and in KCC2a knockout mice to better define circuit-level consequences.

We have added a quantitative analysis of dendritic spines. Using IMARIS, we compared spine density per surface area and the proportion of the four spine types (stubby, mushroom, long, filopodia)

between shNT and shKCC2a neurons and found no significant differences (density p=0.39; spine types p≥0.33), indicating that acute KCC2a knockdown does not by itself remodel spines. These data, and the IMARIS methodology, are now in Fig EV3 and the Methods. The differential spine localization of the two isoforms is intriguing and, as the referee suggests, points to spine-targeting determinants in the KCC2b N-terminus; we now note this differential spine localization of the two isoforms in the Discussion. A full electrophysiological dissection of E/I balance (mEPSCs and E_{GABA} in acute knockdown and in KO neurons) is an important next step that we have flagged as future work rather than attempting here, as it would constitute a study in its own right. (Results, p. 8; Fig EV3; Methods (Image acquisition and analysis): "*We compared the spine density per surface area and the proportion of the four spine types — stubby, mushroom, long, and filopodia — between the shNT and shKCC2a conditions. No statistically significant differences … were observed between conditions (Fig EV3).*")

1.6. Finally, the authors speculate that KCC2a knockout or disruption of KCC2a-SPAK interaction could be an anti-epileptic strategy. However, this interpretation appears inconsistent with their own data showing that KCC2a knockdown in mature neurons reduces KCC2b membrane expression. This point requires clarification, as it has important therapeutic implications.

This is a fair point and we have nuanced the Discussion accordingly. KCC2a disruption has two opposing potential consequences: it lowers baseline KCC2b function in mature neurons (which would tend to weaken inhibition), but it also removes the KCC2a–SPAK coupling through which pathological, WNK-SPAK-driven KCC2 down-regulation occurs during insults such as status epilepticus (which would tend to preserve inhibition). The net effect is therefore expected to differ between acute seizure susceptibility and long-term epileptogenesis and cannot be predicted from the present data; we now state this explicitly and present the therapeutic idea as a hypothesis to be tested rather than a claim. (Discussion, p. 19, lines 445-448: "*Importantly, because KCC2a loss also lowers baseline KCC2b function in mature neurons, the net effect of KCC2a disruption is likely to differ between acute seizure susceptibility and long-term epileptogenesis, and remains to be tested directly.*")

1.7. The authors are strongly recommended to provide a schematic diagram summarizing the proposed model, which would greatly enhance clarity.

A schematic of the proposed model (KCC2a as a developmental, WNK-SPAK-gated switch controlling KCC2b stability and phosphorylation) is provided as the Graphical Abstract with the revised submission. We apologize if it did not reach the referees with the original files.

Referee #2

We thank Referee #2 for the very positive assessment and for the constructive suggestions.

2.1. The conclusions are supported by a strong, multi-part experimental approach using isoform-specific knockdown, knockout mice, pharmacological inhibitors, and functional assays. The evidence shows clearly what happened. To further prove the mechanistic claims and increase the impact, the following experiments are suggested: to perform a rescue experiment with a phospho-dead KCC2b mutant (T1007A). The model shows that in KCC2a-deficient mature neurons where WNK1-CA is overexpressed, the membrane expression of wild-type KCC2b should decrease, but the KCC2b-T1007A mutant should be resistant to this downregulation. However, this mutant might show altered baseline stability. This

experiment would establish a direct causal link between Thr1007 phosphorylation and the observed membrane destabilization, moving beyond correlation.

We considered this experiment carefully but elected not to include it, for two reasons that the referee partly anticipates. First, the full-length WNK1-CA-T2a-GFP construct is large (~13.7 kb) and transfects mature neurons at low efficiency, which makes a quantitative four-condition rescue (±WNK1-CA × WT/T1007A) very difficult to power reliably. Second, as the referee notes, T1007A can itself alter baseline membrane stability, which would confound the comparison. Importantly, the causal link between Thr1007 phosphorylation and KCC2b membrane destabilization is already well established (Friedel et al., 2015; Heubl et al., 2017); our contribution is to show that this regulation requires KCC2a, which we demonstrate by an orthogonal gain-of-function approach (WNK1-CA induction destabilizes KCC2b only when KCC2a is present; Fig. 7). We therefore consider the T1007A rescue a valuable but non-essential confirmation and have noted it as a future direction.

2.2. Try proximity ligation assays (PLA) or co-IP from neuronal lysates to show the KCC2a-SPAK-KCC2b interaction complex in the cell, and how it changes with development or WNK-SPAK activation.

As detailed in our response to Referee #1, point 1.2, we attempted co-IP from brain lysates but were limited by antibody background and low co-IP efficiency on whole-brain extracts. We discuss the converging genetic (KCC2a-KO; Fig. 6) and structural (Chi et al., 2021) evidence supporting the complex in the revised Discussion.

2.3 (minor) – Re-word the sentence on page 9 (proteasome/polyubiquitination). Suggested wording: "These results suggest that KCC2a protects KCC2b from polyubiquitination and subsequent degradation by the proteasome, a degradation pathway that becomes engaged upon KCC2a loss."

We have adopted the referee's suggested wording. (Results, p. 10, lines 233–236: "*These results suggest that KCC2a protects KCC2b from polyubiquitination and subsequent degradation by the proteasome, a degradation pathway that becomes engaged upon KCC2a loss.")*

2.4 (minor) – Add, after Fig. 5E, a caveat noting that MG132 also inhibits calpain at this concentration.

We have added the suggested caveat in the Results. (Results, p. 10, lines 236–238: "*The effect of MG132, which also inhibits calpain at this concentration, suggests the proteasomal pathway is primarily involved, though a contribution from calpain inhibition cannot be fully excluded.")*

Referee #3

We thank Referee #3 for the supportive evaluation and for the helpful, specific points.

3.1. In legend 1: "specific mRNA expression relative to Ywhaz ", and methods: which protein does the housekeeping gene Ywhaz code for? Why was this specific gene chosen as housekeeping gene?

We have clarified this in the Methods. Ywhaz encodes the 14-3-3ζ protein; it was selected because of its high and stable abundance in brain and its established suitability for qPCR normalization, and because in our hands its expression remained stable across the developmental stages examined, unlike actin. (Methods (RT-qPCR), p. 32, line 679: "*Ywhaz encodes the 14-3-3ζ protein … and was selected as reference gene for its high abundance in brain and its established suitability for qPCR normalization*

(Bonefeld et al, 2008); in our hands, its expression remained stable across developmental stages… unlike actin.")

3.2. Figure 5B. The dendrite in DiP-treated culture appear to bear more spine, why? Is it due to how the images were chosen? Is there any effects on spine density?

There is no biological effect: the apparent difference reflects the particular dendritic segment shown, and we have selected a more representative image. We confirmed this by quantifying spine density and spine-type proportions in shNT-expressing neurons ± DiP, finding no effect of DiP on either measure (**Fig. R2A-B**). DiP treatment alone therefore does not alter spine density or spine-type distribution.

3.3. Relative to the same figure: Why is GFP signal stronger in DiP treated dendrites?

This is not a treatment effect. GFP is the transfection/morphology marker, and its absolute intensity varies from cell to cell and with acquisition settings; the apparent difference reflects the representative images, which we have tried to select so that displayed GFP levels are comparable across conditions. Quantification confirms the absence of any effect of DiP on background-subtracted GFP fluorescence or A488 background (**Fig. R2C-D**).

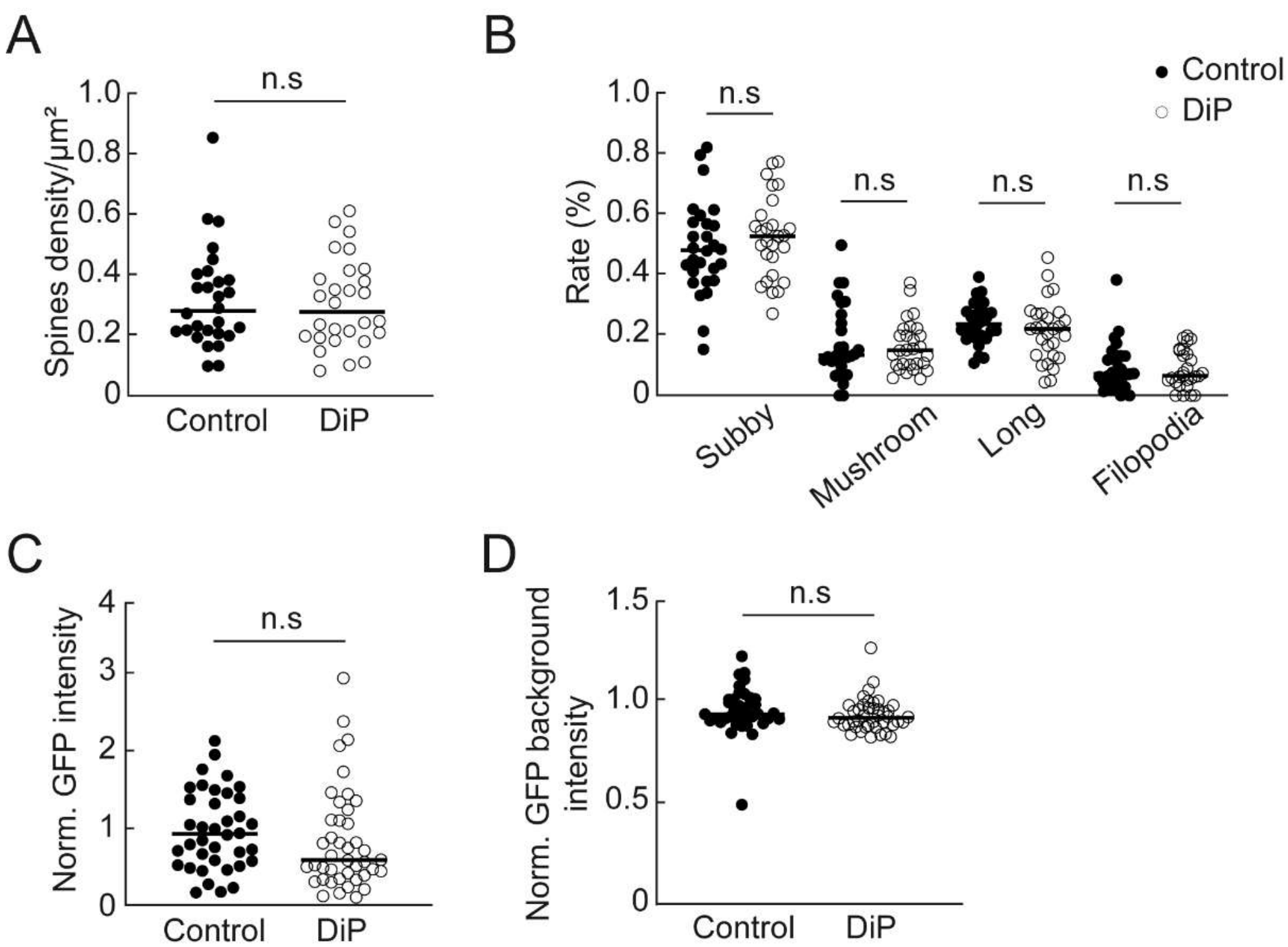

Figure R2. Lack of effect of DiP treatment on spine density and GFP intensity (data from Fig. 5B). Comparison of shNT-expressing neurons in the presence or absence of DiP (n = 27 neurons each, 3 independent cultures). (A) Spine density: no difference between conditions (Mann-Whitney, $p = 0.84$). (B) Proportions of the four spine types: no difference for any type (Mann-Whitney with FDR correction, all $p > 0.05$; control vs +DiP — stubby 48.9 ± 3.0% vs 53.1 ± 2.5%; long 24.2 ± 1.3% vs 21.7 ± 1.9%; mushroom 17.6 ± 2.4% vs 17.8 ± 2.0%; filopodia 9.3 ± 1.5% vs 10.0 ± 1.5%). (C) Background-subtracted GFP fluorescence and (D) A488 background comparison between shNT control and shNT DiP: a two-way ANOVA across the four conditions (shNT/shKCC2a ± DiP) confirmed no significant effect of DiP on either measure ($p = 0.24$ and $p = 0.52$, respectively).

3.4. Please, add the catalog number of the reagents (in particular for commercial antibodies) used in this study.

We have added catalogue numbers for all commercial antibodies and key reagents (Table reagents).

4. In general, the resolution of the images showing dendrites (for example panel 1G, panel 2C,E, 3B, 3E etc) is quite low. Maybe this is due to the PDF conversion, but it would have been more informative to be able to look at higher resolution images.

The apparent low resolution is a compression artefact of the assembled PDF used for review rather than of the source images. High-resolution figure files (≥300 dpi) are provided with the revised submission and can be inspected at full resolution.

We hope that these revisions and clarifications address the referees' concerns and that the manuscript is now suitable for publication.

Dear Dr. Poncer,

Thank you for the submission of your revised manuscript. We have now received the enclosed reports from the referees that were asked to assess it and I am happy to say that both support its publication now. Only a few editorial requests will need to be addressed before we can proceed with the official acceptance of your manuscript:

- Please remove all figures from the ms file. All figures need to be uploaded as individual, high resolution figure files. The figure legends need to be placed after the References.

- Please correct the conflict of interest subheading to "Disclosure and Competing Interests Statement" and place it after the Acknowledgments.

- Please remove the author credits from the ms file. All credits need to be entered during online ms submission.

- The REFERENCE format is not correct: et al needs to be used after 10 author names and the format should not be numerical but alphabetical. Please correct to the EMBO reports reference style.

- The FUNDING INFO in our online submission system is missing "Inserm", please add this with the next ms submission.

- Callouts for Fig. 7C, 7E are missing, please add.

- The EV tables need to be removed from the ms file. The EV tables only need to be uploaded as separate files. Also the Reagents & Tools table needs to be removed from the ms file and only uploaded separately.

- The manuscript sections should be in the following order: Title page - Abstract - Introduction - Results - Discussion - Methods - Data Availability - Acknowledgments - Disclosure Statement & Competing Interests - References - Figure Legends - (Main Tables with legends if applicable) - Expanded View Figure Legends.

- Materials and Methods should be Methods

* Figure Legends - Comments *

- Please note that the legends for figure 6 are not provided in a sequential manner (legend for figure 6C is provided before legend of figure 6B). This needs to be rectified.
- 1. Please note that the exact p values are not provided in the legends of figures 1B,E,F; 2F; 3C,D,F,G; 6B; EV-2B,D. Please provide exact values as reasonable.
- Please note that the box plots need to be defined in terms of centre, bounds of box and percentile in the legends of figures 6B,D,F,H; EV-4B,D.
- Please note that the box plots need to be defined in terms of centre, minima, maxima and whiskers in the legends of figures 1E,F; 2D,F; 3C,F; 4E,F; 5C,E; 7D,F; EV-2B,D; EV-3B,C.
- Please note that the box plots need to be defined in terms of centre in the legends of figures 1B,C.

EMBO press papers are accompanied online by A) a short (1-2 sentences) summary of the findings and their significance, B) 2-3 bullet points highlighting key results and C) a synopsis image that is exactly 550 pixels wide and 300 pixels high. The synopsis image should provide a sketch of the major findings, like a graphical abstract. Please note that text needs to be readable at the final size. Please send us this information along with the final manuscript. (Your synopsis image looks good but the size is not correct. Can you please re-do it? Thank you)

I look forward to seeing a final version of your manuscript as soon as possible.

Kind regards,
Esther

Esther Schnapp, PhD
Senior Editor
EMBO reports

All minor editorial requests have been addressed by the authors.

Dr. Jean Christophe Poncer
Paris Brain Institute
Clinical and Experimental Epilepsy
Hôpîtal Pitié Salpétrière
47 boulevard de l'Hôpital
Paris 75013
France

Dear Dr. Poncer,

I am very pleased to accept your manuscript for publication in the next available issue of EMBO reports. Thank you for your contribution to our journal.

I only have one last suggestion for a small change to the title. Do you agree with this:

Differential isoform-specific control of KCC2 function in developing and mature cortical neurons

Your manuscript will be processed for publication by EMBO Press. It will be copy edited and you will receive page proofs prior to publication. Please note that you will be contacted by Springer Nature Author Services to complete licensing and payment information.

You may qualify for financial assistance for your publication charges - either via a Springer Nature fully open access agreement or an EMBO initiative. Check your eligibility: https://link.springer.com/journal/44319/how-to-publish-with-us

Should you be planning a Press Release on your article, please notify the production team via this form in order to coordinate publication and release dates: https://forms.cloud.microsoft/e/eH4A8JVP6g

If you have any questions, please do not hesitate to contact the Editorial Office. Thank you for your contribution to EMBO Reports.

Best regards,
Esther

Esther Schnapp, PhD
Senior Editor
EMBO reports

>>> Please note that it is EMBO Reports policy for the transcript of the editorial process (containing referee reports and your response letter) to be published as an online supplement to each paper. If you do NOT want this, you will need to inform the Editorial Office via email immediately. More information is available here: https://link.springer.com/partners/embo-press/editorial-policies#Peer%20review

EMBO Press Author Checklist

Corresponding Author Name: Jean-Christophe Poncer	
Journal Submitted to: EMBO reports	
Manuscript Number: EMBOR-2026-63580V1	

USEFUL LINKS FOR COMPLETING THIS FORM
The EMBO Journal - Author Guidelines
EMBO Reports - Author Guidelines
Molecular Systems Biology - Author Guidelines
EMBO Molecular Medicine - Author Guidelines

Reporting Checklist for Life Science Articles (updated January
This checklist is adapted from Materials Design Analysis Reporting (MDAR) Checklist for Authors. MDAR establishes a minimum set of requirements in transparent reporting in the life sciences (see Statement of Task: 10.31222/osf.io/9sm4x). Please follow the journal's guidelines in preparing your
Please note that a copy of this checklist will be published alongside your article.

Abridged guidelines for figures
1. Data
The data shown in figures should satisfy the following conditions:
- the data were obtained and processed according to the field's best practice and are presented to reflect the results of the experiments in an accurate and unbiased manner.
- ideally, figure panels should include only measurements that are directly comparable to each other and obtained with the same assay.
- plots include clearly labeled error bars for independent experiments and sample sizes. Unless justified, error bars should not be shown for technical
- if n<5, the individual data points from each experiment should be plotted. Any statistical test employed should be justified.
- Source Data should be included to report the data underlying figures according to the guidelines set out in the authorship guidelines on Data

2. Captions
Each figure caption should contain the following information, for each panel where they are relevant:
- a specification of the experimental system investigated (eg cell line, species name).
- the assay(s) and method(s) used to carry out the reported observations and measurements.
- an explicit mention of the biological and chemical entity(ies) that are being measured.
- an explicit mention of the biological and chemical entity(ies) that are altered/varied/perturbed in a controlled manner.
- the exact sample size (n) for each experimental group/condition, given as a number, not a range;
- a description of the sample collection allowing the reader to understand whether the samples represent technical or biological replicates (including how many animals, litters, cultures, etc.).
- a statement of how many times the experiment shown was independently replicated in the laboratory.
- definitions of statistical methods and measures:
  - common tests, such as t-test (please specify whether paired vs. unpaired), simple χ2 tests, Wilcoxon and Mann-Whitney tests, can be unambiguously identified by name only, but more complex techniques should be described in the methods section;
  - are tests one-sided or two-sided?
  - are there adjustments for multiple comparisons?
  - exact statistical test results, e.g., P values = x but not P values < x;
  - definition of 'center values' as median or average;
  - definition of error bars as s.d. or s.e.m.

Please complete ALL of the questions below.
Select "Not Applicable" only when the requested information is not relevant for your study.

Materials

Newly Created Materials	Information included in the manuscript?	In which section is the information available? (Reagents and Tools Table, Materials and Methods, Figures, Data Availability Section)
New materials and reagents need to be available; do any restrictions apply?	Yes	Regeants and Tools Table, Materials and Methods

Antibodies	Information included in the manuscript?	In which section is the information available? (Reagents and Tools Table, Materials and Methods, Figures, Data Availability Section)
For **antibodies** provide the following information:<br>- Commercial antibodies: RRID (if possible) or supplier name, catalogue number and or/clone number<br>- Non-commercial: RRID or citation	Yes	Regeants and Tools Table, Materials and Methods

DNA and RNA sequences	Information included in the manuscript?	In which section is the information available? (Reagents and Tools Table, Materials and Methods, Figures, Data Availability Section)
**Short novel DNA or RNA including primers, probes**: provide the sequences.	Yes	Materials and Methods, Table EV

Cell materials	Information included in the manuscript?	In which section is the information available? (Reagents and Tools Table, Materials and Methods, Figures, Data Availability Section)
**Cell lines:** Provide species information, strain. Provide accession number in repository **OR** supplier name, catalog number, clone number, and/**OR** RRID.	Yes	Regeants and Tools Table
**Primary cultures:** Provide species, strain, sex of origin, genetic modification status.	Yes	Materials and Methods
Report if the cell lines were recently **authenticated** (e.g., by STR profiling) and tested for mycoplasma contamination.	Yes	Materials and Methods

Experimental animals	Information included in the manuscript?	In which section is the information available? (Reagents and Tools Table, Materials and Methods, Figures, Data Availability Section)
**Laboratory animals or Model organisms:** Provide species, strain, sex, age, genetic modification status. Provide accession number in repository **OR** supplier name, catalog number, clone number, **OR** RRID.	Yes	Regeants and Tools Table, Materials and Methods
**Animal observed in or captured from the field:** Provide species, sex, and age where possible.	Not Applicable	
Please detail **housing and husbandry conditions**.	Yes	Materials and Methods

Plants and microbes	Information included in the manuscript?	In which section is the information available? (Reagents and Tools Table, Materials and Methods, Figures, Data Availability Section)
**Plants:** provide species and strain, ecotype and cultivar where relevant, unique accession number if available, and source (including location for collected wild specimens).	Not Applicable	
**Microbes:** provide species and strain, unique accession number if available, and source.	Not Applicable	

Human research participants	Information included in the manuscript?	In which section is the information available? (Reagents and Tools Table, Materials and Methods, Figures, Data Availability Section)
If collected and within the bounds of privacy constraints report on age, sex and gender or ethnicity for all study participants.	Not Applicable	

Core facilities	Information included in the manuscript?	In which section is the information available? (Reagents and Tools Table, Materials and Methods, Figures, Data Availability Section)
If your work benefited from core facilities, was their service mentioned in the acknowledgments section?	Yes	Acknowledgements

Design

Study protocol	Information included in the manuscript?	In which section is the information available? (Reagents and Tools Table, Materials and Methods, Figures, Data Availability Section)
If study protocol has been **pre-registered, provide DOI in the manuscript**. For clinical trials, provide the trial registration number **OR** cite DOI.	Not Applicable	
Report the **clinical trial registration number** (at ClinicalTrials.gov or equivalent), where applicable.	Not Applicable	

Laboratory protocol	Information included in the manuscript?	In which section is the information available? (Reagents and Tools Table, Materials and Methods, Figures, Data Availability Section)
Provide DOI OR other citation details if **external detailed step-by-step protocols** are available.	Yes	Results, Materials and Methods, References

Experimental study design and statistics	Information included in the manuscript?	In which section is the information available? (Reagents and Tools Table, Materials and Methods, Figures, Data Availability Section)
Include a statement about **sample size** estimate even if no statistical methods were used.	Yes	No sample size estimate was performed, mentionned in Materials and Methods
Were any steps taken to minimize the effects of subjective bias when allocating animals/samples to treatment (e.g. **randomization procedure**)? If yes, have they been described?	Yes	No randomization was performed, mentionned in Materials and Methods
Include a statement about **blinding** even if no blinding was done.	Yes	Materials and Methods
Describe **inclusion/exclusion criteria** if samples or animals were excluded from the analysis. Were the criteria pre-established?<br><br>If sample or data points were omitted from analysis, report if this was due to attrition or intentional exclusion and provide justification.	Yes	Materials and Methods
For every figure, are **statistical tests** justified as appropriate? Do the data meet the assumptions of the tests (e.g., normal distribution)? Describe any methods used to assess it. Is there an estimate of variation within each group of data? Is the variance similar between the groups that are being statistically compared?	Yes	Materials and Methods

Sample definition and in-laboratory replication	Information included in the manuscript?	In which section is the information available? (Reagents and Tools Table, Materials and Methods, Figures, Data Availability Section)
In the figure legends: state number of times the experiment was **replicated** in laboratory.	Yes	Figure legends
In the figure legends: define whether data describe **technical or biological replicates**.	Yes	Figure legends

Ethics

Ethics	Information included in the manuscript?	In which section is the information available? (Reagents and Tools Table, Materials and Methods, Figures, Data Availability Section)
Studies involving **human participants**: State details of **authority granting ethics approval** (IRB or equivalent committee(s), provide reference number for approval.	Not Applicable	
Studies involving **human participants**: Include a statement confirming that **informed consent** was obtained from all subjects and that the experiments conformed to the principles set out in the WMA Declaration of Helsinki and the Department of Health and Human Services Belmont Report.	Not Applicable	
Studies involving **human participants:** For publication of **patient photos**, include a statement confirming that consent to publish was obtained.	Not Applicable	
Studies involving experimental **animals**: State details of **authority granting ethics approval** (IRB or equivalent committee(s), provide reference number for approval. Include a statement of compliance with ethical regulations.	Yes	Materials and Methods
Studies involving **specimen and field samples:** State if relevant **permits** obtained, provide details of authority approving study; if none were required, explain why.	Not Applicable	

Dual Use Research of Concern (DURC)	Information included in the manuscript?	In which section is the information available? (Reagents and Tools Table, Materials and Methods, Figures, Data Availability Section)
Could your study fall under dual use research restrictions? Please check biosecurity documents and list of **select agents and toxins** (CDC): https://www.selectagents.gov/sat/list.htm	Not Applicable	
If you used a select agent, is the security level of the lab appropriate and reported in the manuscript?	Not Applicable	
If a study is subject to dual use research of concern regulations, is the name of the **authority granting approval and reference number** for the regulatory approval provided in the manuscript?	Not Applicable	

Reporting

The MDAR framework recommends adoption of discipline-specific guidelines, established and endorsed through community initiatives. Journals have their own policy about requiring specific guidelines and recommendations to complement MDAR.

Adherence to community standards	Information included in the manuscript?	In which section is the information available? (Reagents and Tools Table, Materials and Methods, Figures, Data Availability Section)
State if relevant guidelines or checklists (e.g., **ICMJE, MIBBI, ARRIVE, PRISMA**) have been followed or provided.	Not Applicable	
For **tumor marker prognostic studies,** we recommend that you follow the **REMARK** reporting guidelines (see link list at top right). See author guidelines, under 'Reporting Guidelines'. Please confirm you have followed these guidelines.	Not Applicable	
For **phase II and III randomized controlled trials**, please refer to the **CONSORT** flow diagram (see link list at top right) and submit the CONSORT checklist (see link list at top right) with your submission. See author guidelines, under 'Reporting Guidelines'. Please confirm you have submitted this list.	Not Applicable	

Data Availability

Data availability	Information included in the manuscript?	In which section is the information available? (Reagents and Tools Table, Materials and Methods, Figures, Data Availability Section)
Have **primary datasets** been deposited according to the journal's guidelines (see 'Data Deposition' section) and the respective accession numbers provided in the Data Availability Section?	Yes	Data availability (all raw data are available on Zenodo and follow the journal guidelines)
Were **human clinical and genomic datasets** deposited in a public access-controlled repository in accordance to ethical obligations to the patients and to the applicable consent agreement?	Not Applicable	
Are **computational models** that are central and integral to a study available without restrictions in a machine-readable form? Were the relevant accession numbers or links provided?	Not Applicable	
If publicly available data were reused, provide the respective **data citations in the reference list**.	Not Applicable	
