# Supplementary material for: Differential isoform-specific control of KCC2 function in developing and mature cortical neurons
Source: EMBO Rep. 2026 Sep 5;27(20):5837–59. doi: 10.1038/s44319-026-00920-3 (PMC13694278; doi:10.1038/s44319-026-00920-3)
Supplement: Supplementary file 2 [file 44319_2026_920_MOESM5_ESM.pdf]

Expanded View Figures

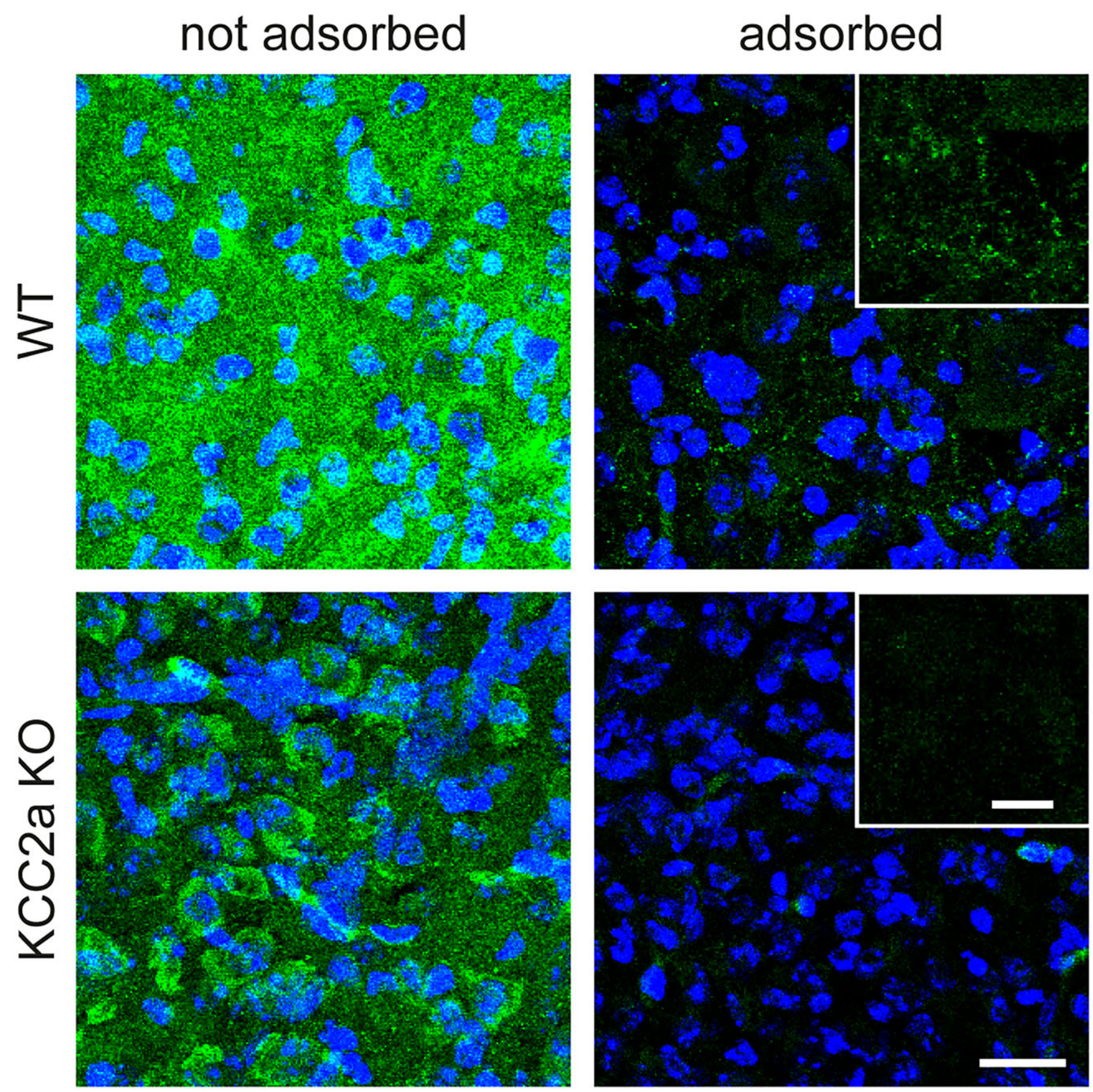

Figure EV1. Specificity of the anti-KCC2a antibody (related to Fig. 1).

Representative confocal maximum projection images of the brainstem from wild-type (WT) and KCC2a-knockout (KCC2a-KO) P9 pups. The sections were immunostained with our anti-KCC2a antibody (green), which was either pre-adsorbed on KCC2a-KO mice brainstems (right) or not pre-adsorbed (left), and then stained with DAPI (blue). Using pre-adsorbed anti-KCC2a antibody greatly reduces nonspecific background staining compared to non-pre-adsorbed antibody. The insets show KCC2a-specific immunostaining in WT but not KCC2a KO samples, at higher magnification. Scale: 20 µm and 10 µm (insets). Two animals per condition, 2 technical replicates.

 © The Author(s)

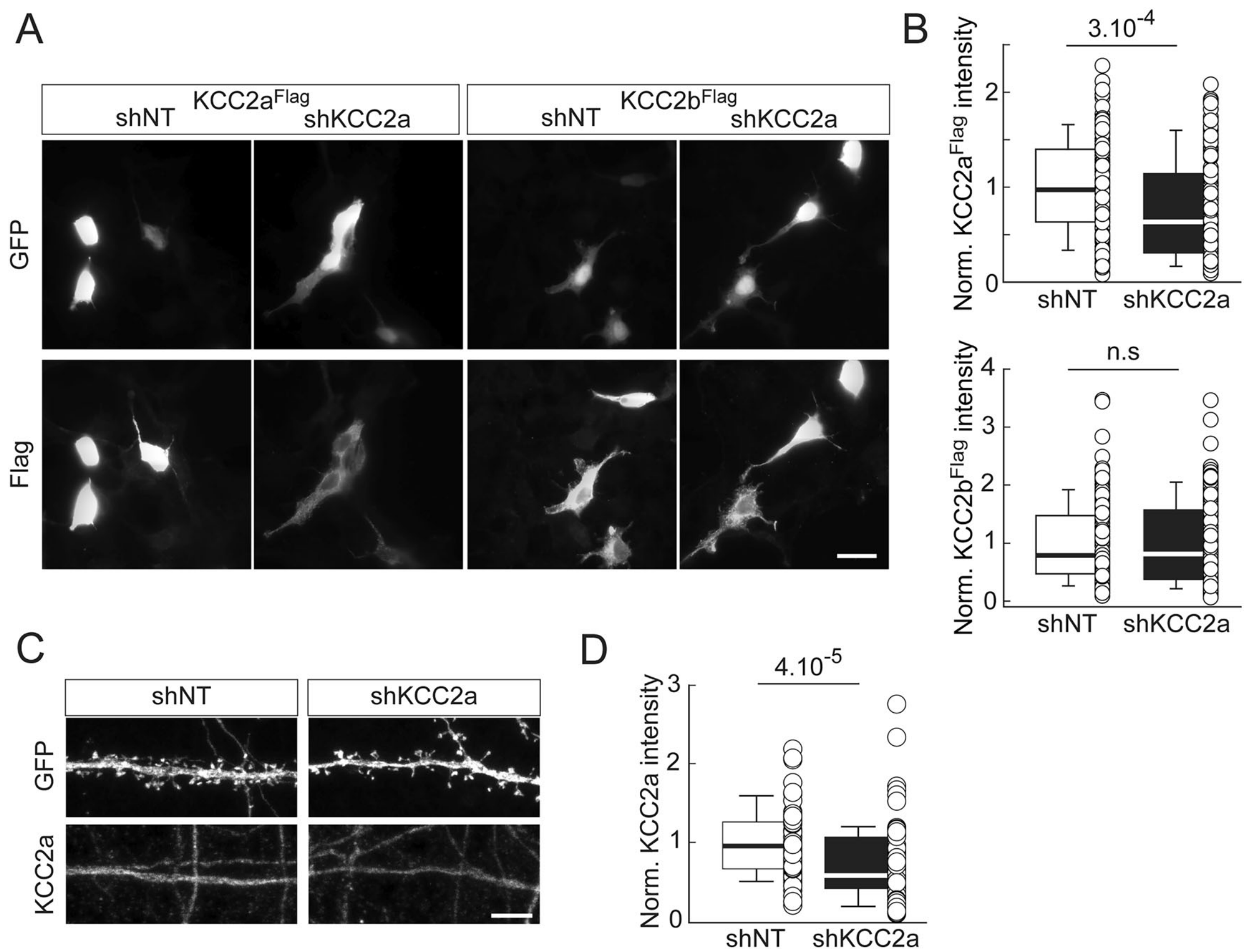

Figure EV2. Specificity of KCC2a knockdown in heterologous cells and hippocampal neurons (related to Fig. 2).

(**A**) Representative epifluorescence images of HEK-293 cells transfected with plasmids encoding either shNT or shKCC2a and either KCC2a-Flag or KCC2b-Flag. Cells were immunostained for Flag and GFP. Scale: 20 µm. (**B**) Summary boxplot showing the intensity of Flag immunostaining in HEK-293 cells expressing scrambled (shNT) or KCC2a-specific (shKCC2a) shRNAs and recombinant KCC2a-Flag (left) or KCC2b-Flag (right). shNT KCC2a-Flag, $n = 131$; shKCC2a KCC2a-Flag, $n = 126$; shNT KCC2b-Flag, $n = 155$; shKCC2a KCC2b-Flag, $n = 153$ cells, from three independent cultures. Mann–Whitney rank-sum test. All data are normalized to the corresponding shNT. (**C**) Representative confocal maximum projection images of dendritic sections of neurons transfected with plasmids encoding shNT or shKCC2a and immunostained for endogenous KCC2a. Scale: 5 µm. (**D**) Boxplot showing the intensity of endogenous KCC2a immunofluorescence in neurons transfected with shNT or shKCC2a_1 + 3. shNT, $n = 73$; shKCC2a, $n = 74$ cells, from five independent cultures (mean effect per culture: −38%, +17%, −13%, −41%, −76%). Mann–Whitney rank-sum test. Box plots show the median (centre line), the 25th and 75th percentiles (box bounds) and the 10th and 90th percentiles (whiskers); all individual data points, including minima and maxima, are displayed alongside the box (see "Methods"). *P* values are provided on the graphs.

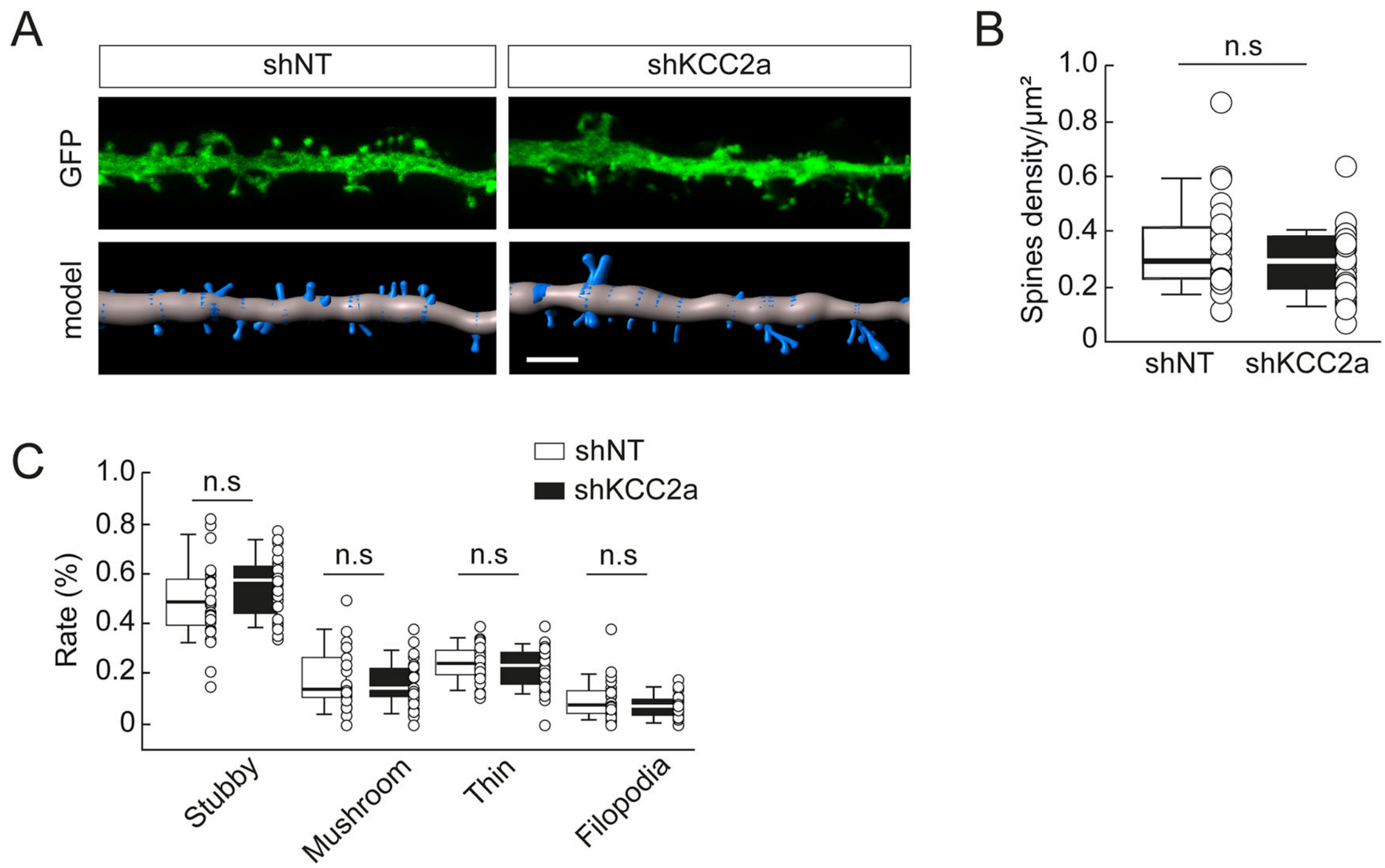

Figure EV3. KCC2a knockdown does not affect dendritic spines in mature hippocampal neurons (related to Fig. 3).

(**A**) Representative confocal maximum projection images and 3D reconstruction of dendritic sections of neurons transfected with plasmids encoding shNT or shKCC2a and immunostained for GFP. The 3D reconstruction was used for spine analysis. Scale: 3 µm. (**B**) Boxplot showing the spine density per dendrite surface area of neurons transfected with shNT or shKCC2a_1 + 3. Mann–Whitney rank-sum test. (**C**) Boxplot showing the proportion of each spine type (stubby, mushroom, long, and filopodia) within neurons transfected with plasmids encoding shNT or shKCC2a. Mann–Whitney rank-sum test, *P* value corrected for multiple comparisons using the Benjamini–Hochberg false discovery rate (FDR) procedure. Complementary linear mixed-model analyses were also performed with independent cultures as a random intercept. shNT, $n = 28$; shKCC2a, $n = 28$, from three independent cultures. Box plots show the median (centre line), the 25th and 75th percentiles (box bounds) and the 10th and 90th percentiles (whiskers); all individual data points, including minima and maxima, are displayed alongside the box (see "Methods").

 © The Author(s)

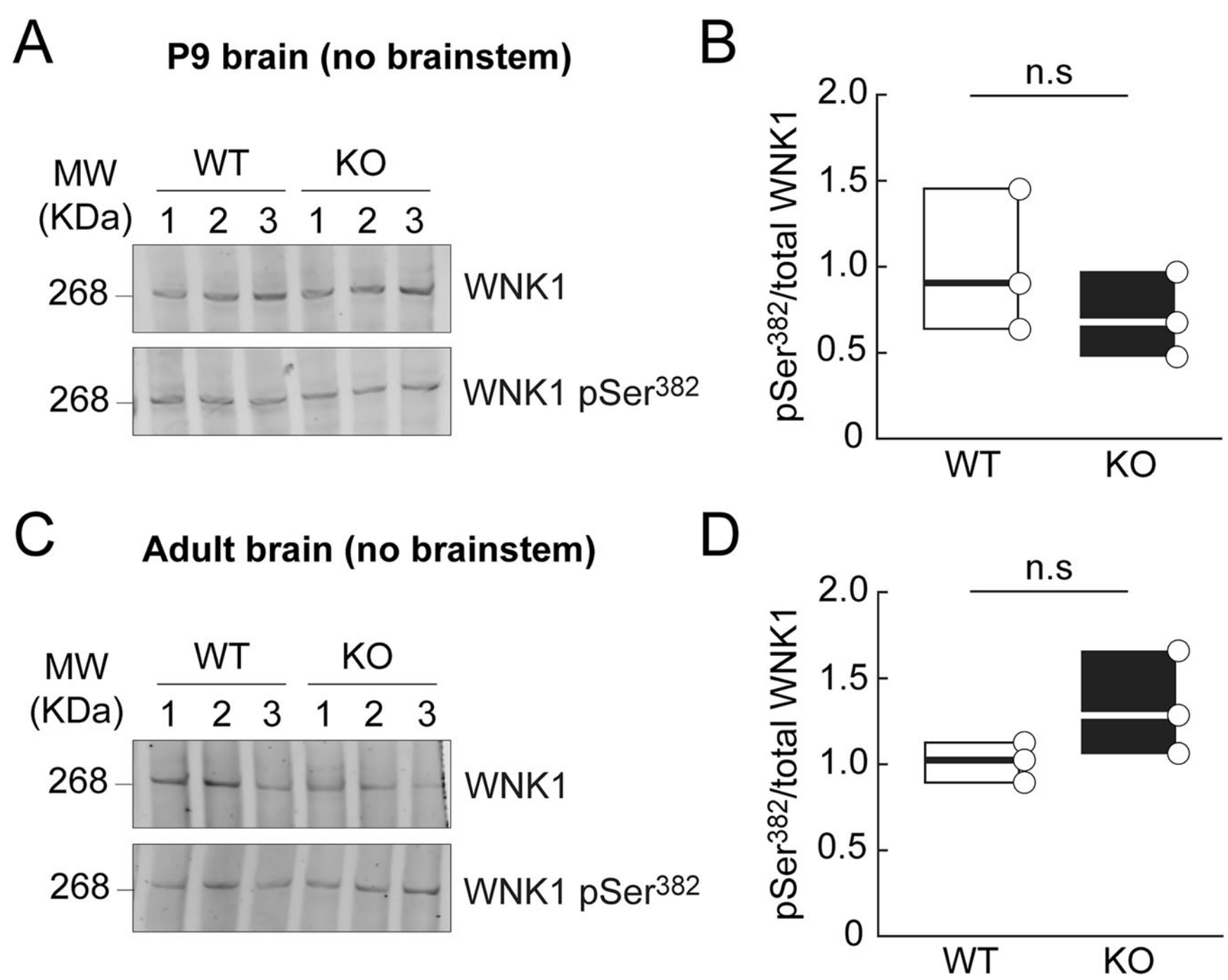

Figure EV4. KCC2a knockout does not affect WNK1 Ser382 phosphorylation (related to Fig. 6).

(**A**, **C**) Western blots of brain extracts (excluding the brainstem) from P9 and adult KCC2a-KO mice and WT littermates. Each membrane was probed for WNK1 or WNK1 Ser^{382P}. Samples which are compared derive from the same experiment and membranes were processed in parallel. (**B**, **D**) Summary boxplots showing the phosphorylated WNK1 immunofluorescence intensity normalized to WNK1 in KCC2a-KO and WT mice. All results are normalized to WT. WT, $n = 3$; KO, $n = 3$ animals; 2–3 technical replicates (for P9-adult, respectively) on the same brain extracts. Two-tailed unpaired *t* test. Samples being compared derive from the same experiment and membranes were processed in parallel. Box plots show the median (centre line) and the 25th and 75th percentiles (box bounds). With $n = 3$ per group, no whiskers are displayed and all individual data points are shown alongside the box.

© The Author(s)
