# Supplementary material for: Differential isoform-specific control of KCC2 function in developing and mature cortical neurons
Source: EMBO Rep. 2026 Sep 5;27(20):5837–59. doi: 10.1038/s44319-026-00920-3 (PMC13694278; doi:10.1038/s44319-026-00920-3)
Supplement: Supplementary file 3 [file 44319_2026_920_MOESM1_ESM.docx]

**Table EV1. Plasmids ratio used for transfection**

| **Experiment** | **Plasmids ratio for each condition (C)** |
| --- | --- |
| Expression and relative membrane expression of recombinant KCC2a- and KCC2b-Flag | C1: KCC2a-Flag (0.8) + EGFP (0.2)  C2: KCC2b-Flag (0.8) + EGFP (0.2) |
| Efficacy and specificity of KCC2a-specific shRNAs | C1: shKCC2a_1-GFP (0.2) + KCC2a-Flag (0.8)  C2: shKCC2a_3-GFP (0.2) + KCC2a-Flag (0.8)  C3: shNT-GFP (0.2) + KCC2a-Flag (0.8)  C4: shKCC2a_1-GFP (0.1) + shKCC2a_3-GFP (0.1) + KCC2a-Flag (0.8) |
| Effect of KCC2a knockdown on endogenous KCC2 expression | C1: shKCC2a_1-GFP (0.5) + shKCC2a_3-GFP (0.5)  C2: shNT-GFP (1) |
| Effect of KCC2a knockdown on recombinant KCC2b expression and membrane expression | C1: shKCC2a_1-GFP (0.1) + shKCC2a_3-GFP (0.1) + KCC2b-flag (0.8)  C2: shNT-GFP (0.2) + KCC2b-flag (0.8) |
| Effect of WNK1 activation on recombinant KCC2b membrane expression (control) | C1: WNK1-CA-GFP (0.5) + KCC2b-Flag (0.5)  C2: WNK1-CA-GFP (0.45) + KCC2b-Flag (0.45) + EGFP (0.1) |
| Effect of WNK1 activation on recombinant KCC2b membrane expression (KCC2a knockdown) | C1: WNK1-CA (0.4) + KCC2b-Flag (0.4) + shNT (0.2)  C2: WNK1-CA (0.4) + KCC2b-Flag (0.4) + shKCC2a_1-GFP (0.1) + shKCC2a_3-GFP (0.1) |
