# Supplementary material for: Differential isoform-specific control of KCC2 function in developing and mature cortical neurons
Source: EMBO Rep. 2026 Sep 5;27(20):5837–59. doi: 10.1038/s44319-026-00920-3 (PMC13694278; doi:10.1038/s44319-026-00920-3)
Supplement: Supplementary file 4 [file 44319_2026_920_MOESM2_ESM.docx]

**Table EV2. Primary and secondary antibodies used for immunostainings**

| **Experiment** | **Antibodies** |
| --- | --- |
| Endogenous KCC2a and KCC2 expression in mature neurons | Rabbit anti-KCC2 (1:500, Millipore)  Rabbit anti-KCC2a (1:750: Airaksinen lab)  Donkey anti-Rabbit Cy3 (1:400, Jackson ImmunoResearch)  Donkey anti-Rabbit A647 (1:400, Jackson ImmunoResearch) |
| Expression and relative membrane expression of recombinant KCC2a- and KCC2b-Flag  Effect of KCC2a knockdown on recombinant KCC2b expression and membrane expression  Effect of WNK1 activation on recombinant KCC2b membrane expression (+/- KCC2a) | Mouse anti-Flag M2 (1:1000, Sigma-Aldrich)  Chicken anti-GFP (1:500, Chemicon)  Donkey anti-Mouse Cy3 (1:400, Jackson ImmunoResearch)  Donkey anti-Mouse A647 (1:400, Jackson ImmunoResearch)  Donkey anti-Chicken A488 (1:400, Jackson ImmunoResearch) |
| Efficacy of KCC2a-specific shRNAs (recombinant KCC2a-Flag) | Mouse anti-Flag M2 (1:1000, Sigma-Aldrich)  Donkey anti-Mouse Cy3 (1:400, Jackson ImmunoResearch) |
| Efficacy of KCC2a-specific shRNAs (endogenous KCC2a) | Rabbit anti-KCC2a (1:750: Airaksinen lab)  Donkey anti-Rabbit Cy3 (1:400, Jackson ImmunoResearch) |
| Specificity of KCC2a-specific shRNAs | Mouse anti-Flag M2 (1:1000, Sigma-Aldrich)  Donkey anti-Mouse A647 (1:400, Jackson ImmunoResearch) |
| Effect of KCC2a knockdown on endogenous KCC2 expression | Rabbit anti-KCC2 (1:500, Millipore)  Donkey anti-Rabbit Cy3 (1:400, Jackson ImmunoResearch) |
