# Supplementary material for: Differential isoform-specific control of KCC2 function in developing and mature cortical neurons
Source: EMBO Rep. 2026 Sep 5;27(20):5837–59. doi: 10.1038/s44319-026-00920-3 (PMC13694278; doi:10.1038/s44319-026-00920-3)
Supplement: Supplementary file 5 [file 44319_2026_920_MOESM3_ESM.docx]

**Table EV3. Primers used for the RT-qPCR**

| **Variant** | **Primer pair** |
| --- | --- |
| Variant 1 (*KCC2a*) | F: GCCACTTGTGCGATCCCG  R: TCCGTGCTGTTGATGAAGGG |
| Variant 2 (*KCC2b*) | F: CAGCCACCATGCTCAACAAC  R: TCTCTCCCCTTCTCCGTGTC |
| *Ywhaz* | F: TTGAGCAGAAGACGGAAGGT  R: GAAGCATTGGGGATCAAGAA |
